# Molecular engineering of bistetrafluoropyridine aryl ethers: a general platform for stable and programmable amorphous organic solids

**DOI:** 10.1039/d6ra07696c

**Published:** 2026-09-18

**Authors:** Nathan J. Weeks, Anika A. Speicher, Scott T. Iacono

**Affiliations:** a Department of Chemistry and Chemistry Research Center, Laboratories for Advanced Materials, United States Air Force Academy Colorado Springs Colorado 80840 USA scott.iacono@afacademy.af.edu nathan.weeks@afacademy.af.edu

## Abstract

We report a highly efficient strategy for the synthesis of a novel class of amorphous, small-molecule organic glass formers (MOGs) derived from pentafluoropyridine (PFP). Utilizing a single-step, atom-economical, and highly scalable nucleophilic aromatic substitution S_N_Ar platform, a series of bistetrafluoropyridine aryl ethers (BTFPAEs) were prepared in excellent yields. This orthogonal approach leverages molecular asymmetry and rigid core architecture to effectively suppress crystallization, addressing a historical limitation of traditional MOGs. Thermal analysis revealed two discrete regimes of glassing behavior. Class I systems exhibit exceptional morphological stability, undergoing an irreversible transition to a robust, transparent amorphous glass upon initial melting with glass transition temperatures (*T*_g_) precisely tunable between −15 °C and 53 °C. Exploiting this stability, we demonstrate the fabrication of homogeneous, melt-blended binary glasses with predictable, programmable *T*_g_ profiles that adhere strictly to the Fox equation. Class II systems yield highly versatile, metastable semi-amorphous matrices through controlled cooling dynamics. This work establishes a predictable structure–property relationship governing molecular symmetry, internal flexibility, and phase behavior, demonstrating that these resilient BTFPAE architectures hold significant promise as tailorable host matrices for a range of advanced material applications.

## Abstract 2

A single-step, atom-economical synthesis produces pentafluoropyridine-derived amorphous organic glass formers. Using molecular asymmetry to prevent crystallization, they offer tunable glass transition temperatures for stable advanced materials.

## Graphical abstract

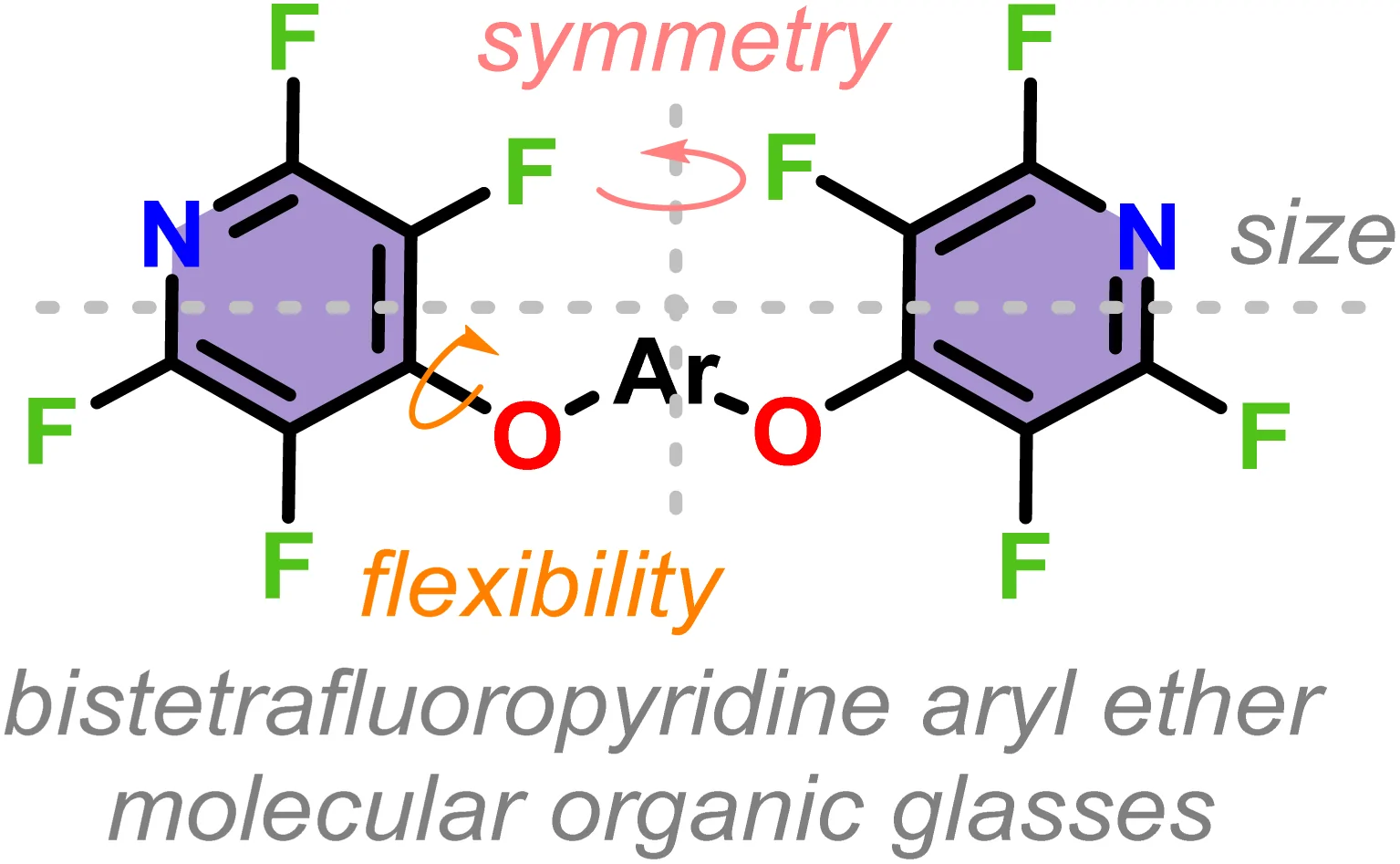

## Introduction

Pentafluoropyridine (PFP) synonymously referred to as perfluoropyridine possesses unique electronic heteroaromatic configuration and its reactivity has been thoroughly studied.^1–4^ PFP has been utilized for highly efficient, regio-selective transformations for nucleophilic aromatic substitution,^5^ photochemical additions^6^ dearylation,^7^ fluorination,^8^ deoxyfluorination,^9^ hydrodefluorination,^10^ halogen exchange,^11^ and phenolic protecting group strategies.^12^ Furthermore, a recent comprehensive review demonstrates PFP as a highly adaptable intermediate for varied polymer architectures and their respective applications.^13^ Use of PFP reactivity has been further explored in expanded areas of broad interest including pharmaceuticals,^14^ crystal engineering,^15^ biomass quantification,^16^ oxy-arylation of carbohydrates,^17^ peptide modification,^18^ cancer therapeutics,^19^ organo-pollutant absorbents,^20^ extended life-cycle electrolytes,^21^ and fluoroelastomers for cryogenic applications.^22^

Organic molecules that possess defined crystalline melt transitions followed by the formation of stable amorphous glassy states can undergo vitrification that allows molecules to become structurally interlocked in random configurations by overcoming intermolecular forces that would normally induce crystallization. This phenomenon was formally introduced and systematically studied by Molaire and Johnson establishing an area room temperature stable, non-crystallizable organic monomeric glasses.^23^ Since then, the development of requirements for small molecules to possess glass-forming properties include a combination of large molecular weight, the presence of aromatic rings, molecular asymmetry, a large number of rotatable bonds, branched carbon skeletons, and/or a combination of electronegative atoms.^24–27^ Vitrification is common in high molecular weight, chain-extended linear or network architectures such as oligomers or polymers and can be quantified by the thermal or mechanical glass transition temperature (*T*_g_). Molecular organic glasses (MOGs) also exhibit a *T*_g_ and have been reported for their promising applications as amorphous solid dispersions to improve important variables of biological drug efficacy such as transport and dissolution.^28–31^ The value of the amorphous molecular organic compounds has also expanded into broader materials applications such as incorporating liquid crystal mesogens for information storage materials,^32^ host matrices for chromophore guests in optoelectronic materials,^33^ molecular resists,^34^ organic eutectics for polymer plastic replacement,^35^ hybrid organic–inorganic perovskites,^36^ fluorescent glasses,^37–40^ non-linear optics,^41^ precursors for facile polymer processing,^42^ charge transporting devices,^43^ fulvene-based dyes,^44,45^ radioluminescence,^46^ active fillers in energy storage devices,^47^ and molecular glass photoresists for lithography.^48^

**Fig. 1:**
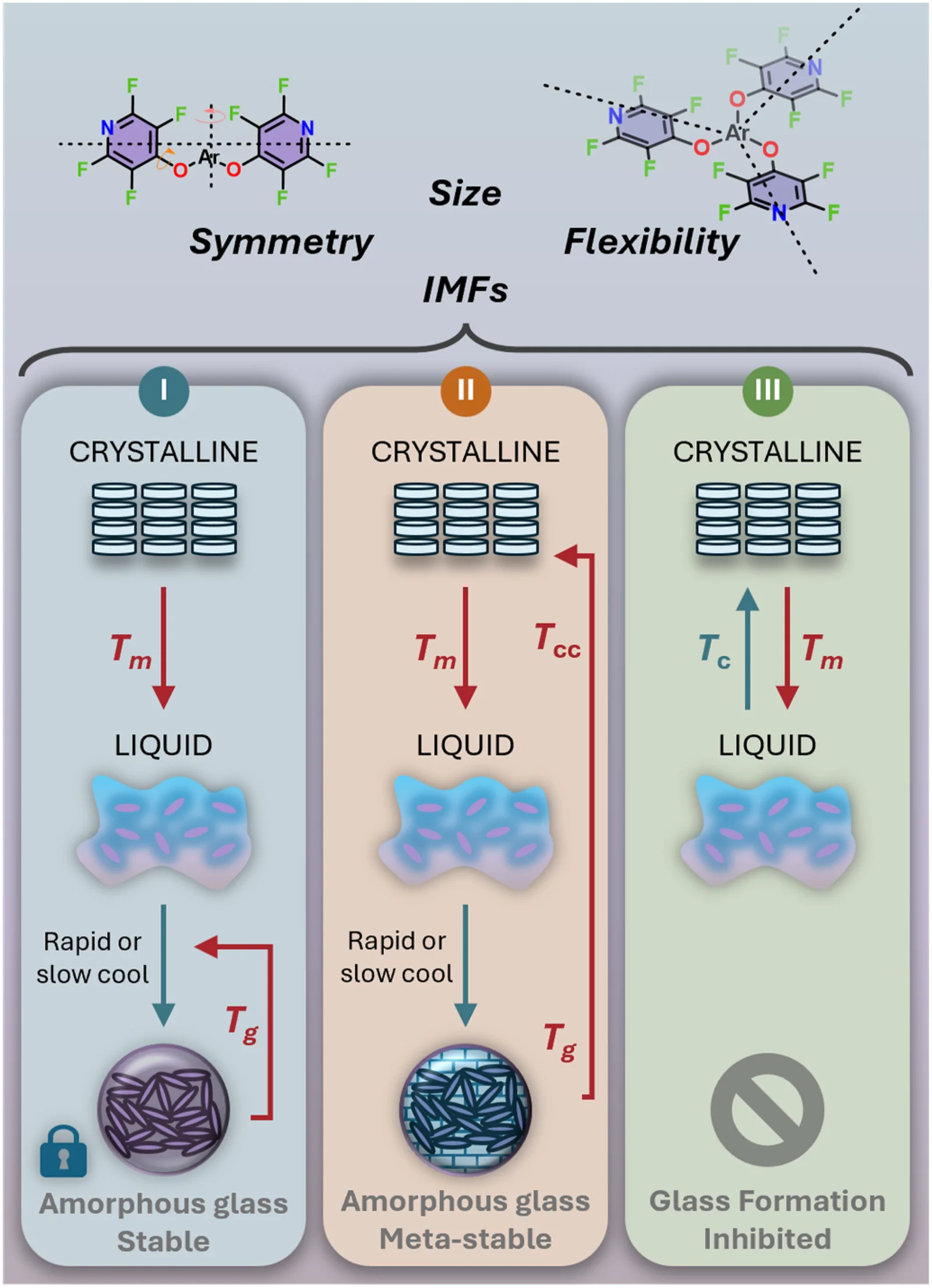
Schematic representation of the complex thermal cycling behavior of the BTFPAE MOGs driven by aryl ether spacer effects leading to defined classes with thermal properties influenced by parameters such as symmetry, size, and flexibility.

**Fig. 2:**
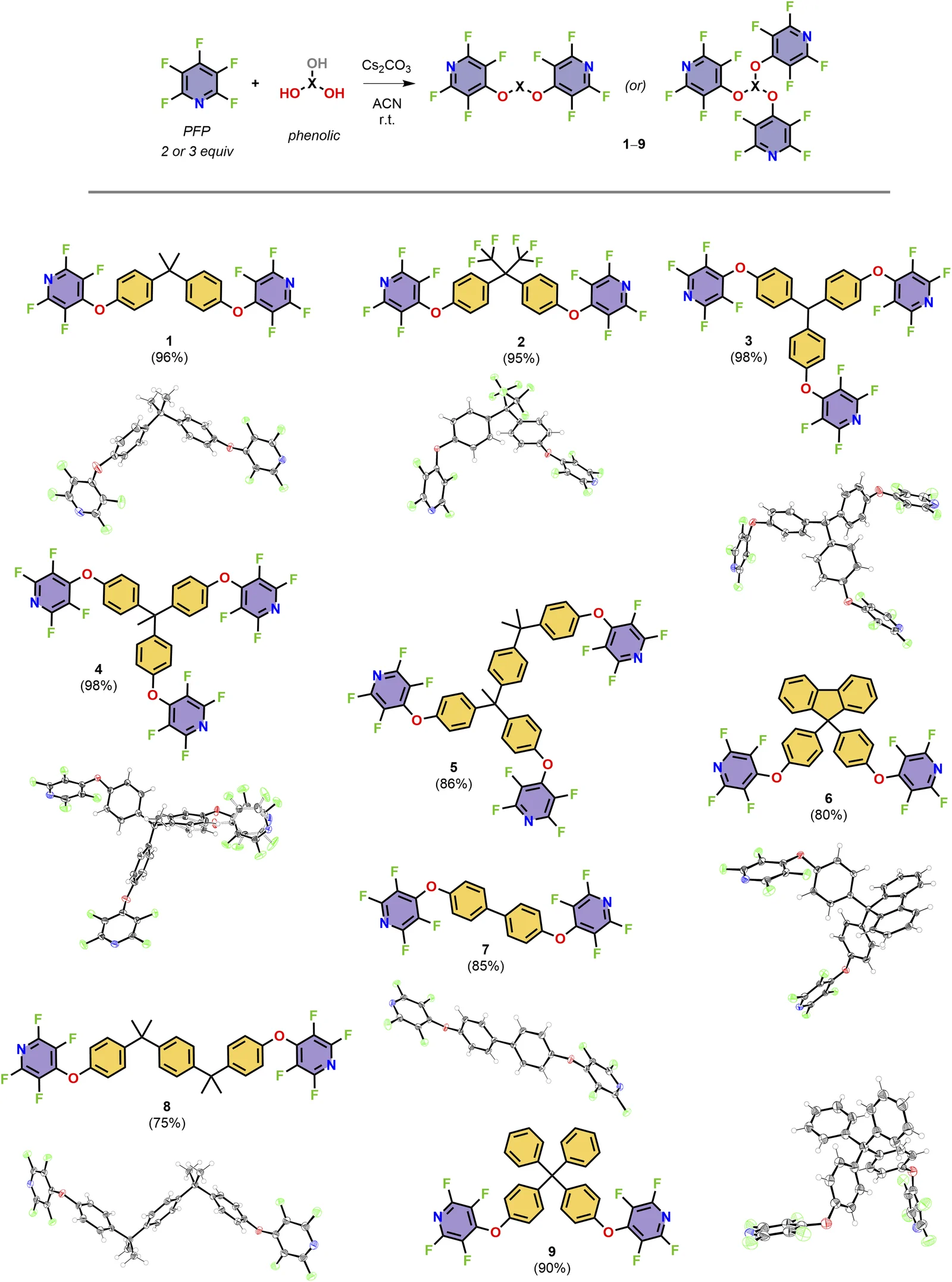
Synthesis of 1–9 from the base-mediated S_N_Ar of biphenols to pentafluoropyridine. ORTEP representation of obtained crystal structures interspersed whereby thermal ellipsoids are shown at 50% probability. X-ray structures of compounds 1, 2 and 4 and have been previously reported.^49^

In our previous study, we established PFP as a core scaffold for nucleophilic aromatic substitution (S_N_Ar) step-growth polymerizations.^49^ That foundational work demonstrated that reacting PFP with bisphenols using a mild cesium carbonate base yields 2,3,5,6-tetrafluoropyridine (TFP) end-capped aryl ether monomers in nearly quantitative yields. When these TFP monomers were subsequently reacted with additional bisphenols, they underwent a second S_N_Ar chain advancement at room temperature to form high-molecular-weight linear and networked fluorinated pyridine aryl ether polymers. Notably, during the characterization of those intermediate TFP monomers, specifically compounds 1, 2, and 4 (Fig. 2) we observed non-reversible melting-crystallization phase behavior upon initial heating. Upon cooling, these species resisted recrystallization to yield clear, amorphous glassy solids with well-defined glass transition temperatures (*T*_g_), as the steric bulk and fluorinated nature of the TFP endcaps disrupted close molecular packing. Similarly, another previous study utilized TFP as well as other aromatic fluorocarbons to drive phase separation in spiro-type molecular glasses.^50^ Building directly upon these observations, the current work shifts focus from using TFP species strictly as reactive intermediates for polymerizations to systematically investigating them as a standalone platform for small-molecule organic glass formers. In this work, we have synthesized a series of bistetrafluoropyridine aryl ethers (BTFPAEs) from PFP in a single, high yielding, multi-gram scalable step from commercial bisphenols that meet the conditions of molecular size, symmetry, and flexibility as molecular organic glass formers (Fig. 1). The irregular high molecular weight structures of BTFPAEs upon initial melting, then controlled cooling, hinder their molecular rearrangement, thus eliminating (class I: glass formers) or subduing crystallization (class II: metastable-glass formers) leading to transparent, glassy structures at room temperature with well-defined, structure dependent glass transition temperature. As a comparison, class III BTFPAE-based non-class formers were also prepared utilizing this strategy demonstrating reversible melt transitions. Insight gained from this study could expand the application of these BTFPAEs as a potential new class of amorphous host matrix materials for optical applications.

## Results and discussion

### Synthesis and characterization of BTFPAEs 1–9

New bistetrafluoropyridine aryl ethers (BTFPAEs) 3 and 5–9 were prepared using the procedure previously reported for 1, 2 and 4 (ref. 49) in overall good to excellent isolated yields (75–95%) by regio-selective, base-mediated nucleophilic aromatic substitution exclusively on the 4-position of pentafluoropyridine (PFP) with selected commercial bis- or trisphenols in acetonitrile (scheme in Fig. 2). Purification and isolation of 1–9 was accomplished simply by removal of carbonate salts using filtration, solvent evaporation, and recrystallization of the crude products from hot ethanol to afford white microcrystalline powders. Repeated synthesis demonstrated that the reactions can be performed at large scale, up to 10–20 g quantities. Characterization using ^1^H, ^13^C, ^19^F NMR spectroscopy in addition to gas chromatography coupled mass spectrometry confirmed purity (>98%) and structure of compounds 1–9. Furthermore, high-quality crystals were obtained from 1–9 (excluding only 5) upon slow recrystallization from hot ethanol and their elucidation using single crystal X-ray diffraction analysis afforded their embedded ORTEP structures as shown in Fig. 2.

### Melt transition analysis of BTFPAEs 1–9

Initially, melting point (*T*_m_) analysis was performed using differential scanning calorimetry (DSC) on 2,3,5,6-tetrafluoropyridine (TFP)-derivatized compounds 1–9 and are listed in Table 1. Overall, the installation of TFP moiety onto respective bis- or triphenols significantly suppressed melt transitions due to the lack of intermolecular H-bonding between the phenol units. For example, 4,4′-(propane-2,2-diyl)diphenol (*i.e.* bisphenol A) has a literature *T*_m_ of 158 °C and upon installation of TFP as end-capped units, the *T*_m_ of 1 is lowered to 77 °C. In the case of 4, the *T*_m_ is observed at 146 °C which is 102 °C lower than the phenol starting material 1,1,1-tris(4-hydroxyphenyl)ethane of 248 °C and possessed the highest *T*_m_ among the bis/trisphenols used in this study.

**Table 1:** Thermal properties of bistetrafluoropyridine aryl ethers 1–9

Entry	*T*_m_ a (°C)	Δ*H*_m_^a^ (J g^−1^)	*T*_g_ b (°C)	*T*_c_ c (°C)	Δ*H*_c_^c^ (J g^−1^)	*T*_cc_ d (°C)	Δ*H*_cc_^d^ (J g^−1^)
1	77^e^	50	−15	—	—	—	—
2	109^e^	53	0	—	—	85	28
3	121^e^	57	17	—	—	—	—
4	146^e^	56	21	—	—	—	—
5	101^e^	37	32	—	—	—	—
6	166^f^	70	53^g^	—	—	151	44
7	205^f^	75	—	82	42	—	—
8	187^f^	107	6^g^	—	—	47	48
9	227^f^	81	34^g^	149	67	75^g^	14^g^

a DSC (first heat cycle 10 °C min^−1^).

b DSC (third heating cycle).

c DSC (first cooling cycle).

d DSC (second cooling cycle).

e DSC (cycle temperature range: −50 to 180 °C).

f DSC (cycle temperature range: −50 to 250 °C).

g DSC (rapid cool 50 °C min^−1^).

While comparing the melt transitions of 1–9 with their analogous bis/trisphenols is beyond the scope of the present work, it is important to demonstrate the melting points (*T*_m_) of the purified TFP-derived compounds ranged from 77 °C to only as high as 227 °C which are ideal for solventless melt processing techniques as demonstrated later in this work. Comparing melt transitions of structures of 1–9, some inherent trends within this substrate pool were observed and noted as follows. The presence of the bulkier hexafluoroisopropylidene moieties of 2 showed a marked increase (32 °C) in *T*_m_ compared with the non-fluorinated analogue of 1. Compared with the symmetrical trifunctionalized TFP compounds 3 and 4, compound 5 possess a lower *T*_m_ due to lack of symmetry. Compounds 6–9 possess the higher *T*_m_ compared with 1–5, the highest being 9*T*_m_ of 227 °C, which is due to a combination of favorable symmetry, molecule packing, high polyaromatic character and strong hydrogen bonding interactions.

### Class I glass formers: heat induced stable glass formation of BTFPAEs 1–6

Thermal analysis using DSC and thermogravimetric analysis (TGA) were performed on 1–6 and selected properties are shown in Tables 1 (DSC) and 2 (TGA). For this substrate pool, the installation of the 2,3,5,6-tetrafluoropyridine moiety promoted enough molecular entanglement in addition to their high molecular weight (ranging from 526 amu for 1 to as high as 872 amu for 5) which mitigated favorable intermolecular forces upon initial heating causing an irreversible *T*_m_. This impaired recrystallization upon cooling, which in turn produced exclusively amorphous molecular glass species that are transparent, brittle films. Additionally, the complex symmetry of some in this class is also thought to be a driving factor in promoting stable glass formation. Upon subsequent heating and slow cooling cycles (10 °C min^−1^) compounds 1–5 produced a glass transition temperature (*T*_g_) along with a reversible vitrification temperature, respectively. Compound 2 required an additional heating and cooling cycle (10 °C min^−1^) to suppress its small cold crystallization temperatures (*T*_cc_) at 85 °C followed by *T*_m_ at 103 °C. Compound 6 has a *T*_cc_ at 151 °C and 
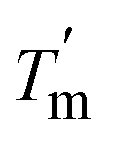
 of 171 °C and required an additional rapid cooling cycle (50 °C min^−1^) to produce a stable glass (see Fig. S26 and S30 respectively for 2 and 6). The presence of this event for 2 and 6 indicates the compounds initially form a vitrified, glassy state, but their respective pendant bulky functional groups, as well as idiosyncratic IMFs, induce favorable stacking and lead to cold crystallization upon heating. Subsequent heating and cooling cycles further suppress this event, rendering them entirely amorphous, stable glasses. Also, compound 6 is much higher *T*_g_ (53 °C) than the other class I compounds. Its *T*_g_ is 21 °C higher than 5 (the bulkiest compound) likely due to its pendant cardo fused ring structure. Cardo-type polymers typically have high glass transition temperatures due to the sterically driven large rotational potential of the pendant ring structure off the backbone.^51,52^ It is likely that in its vitreous form the same effect is at play to some extent, leading to a much higher *T*_g_ of 6.

**Table 2:** Onset decomposition temperatures of 1–6 before and after glass formation

Entry	*T*_d_ a (°C)	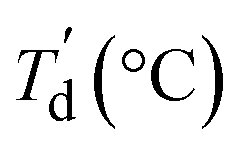 a	Char^a^ (%)
1	270	270	0.3
2	248	252	—
3	350	353	9.0
4	348	364	—
5	389	382	1.0
6	320	350	1.5

a TGA (10 °C min^−1^, 25 °C to 900 °C) in nitrogen.

**Fig. 3:**
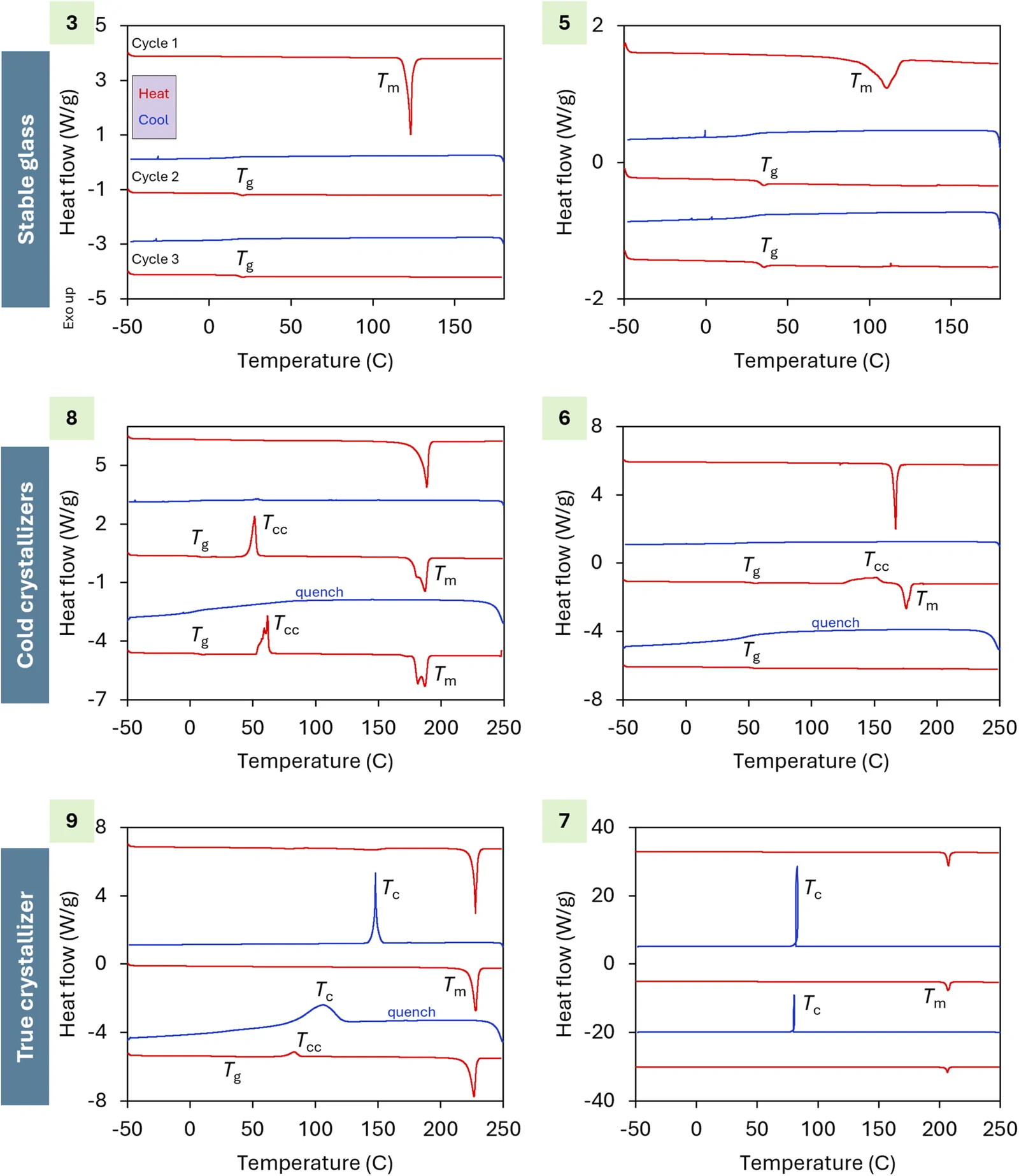
DSC thermograms of representative compounds of each glass forming class type.

Representative DSC thermograms of each class and subclass of compound are shown in Fig. 3. Compounds 3 and 5 are shown as representative of the stable glass formers (class I). Both compounds have a *T*_g_ after one melt/cool cycle at the 10 °C min^−1^ rate, which is then stable for all subsequent cycling. Compound 5 is unique in this class as it is the least symmetric and largest by molecular weight (872 amu) thus possessing all the properties thought to be ideal for amorphous glass formation. In fact, compound 5 is the only compound for which X-ray quality crystals were not obtained. Upon slow solvent evaporation from common organic solvents (THF, CHCl_3_, and EtOAc), 5 formed exclusively a solid, transparent glassy film. The enthalpy of the melt transition (Δ*H*_m_) for 5, from the as-purified microcrystalline powder, is also the lowest out of all nine BTFPAEs compounds at 37 J g^−1^ indicating weak intermolecular interactions (Table 1). Given these observations, substrates 1–6 are considered in this study to be class I glass formers as outlined above in the introduction of this work whereby their molecular size, symmetry, flexibility, and steric considerations lead to a wide range of *T*_g_'s ranging from −15 °C to 53 °C.

The thermal decomposition (*T*_d_) of the bistetrafluoropyridine aryl ether molecular glasses are strictly governed by the structural architecture of their central phenolic cores, establishing a clear link between chemical configuration and degradation resistance. Thermogravimetric analysis as shown in Table 2 demonstrates that transitioning from weaker aliphatic linkages (compounds 1 and 2) to fully conjugated, polyaromatic cores (compounds 3–6) elevates the onset decomposition temperature by nearly 100 °C due to higher bond dissociation energies. Additionally, there are negligible changes in decomposition temperatures among compounds 1–6 in their vitrified state after DSC thermal treatment. Overall, the onset of thermal decomposition is significantly higher than the reported melting points for each respective compound enabling them suitable candidates for bulk melt processing into amorphous glasses.

### Melt blending and composite glass transition temperatures of class I glass formers 1–6

**Fig. 4:**
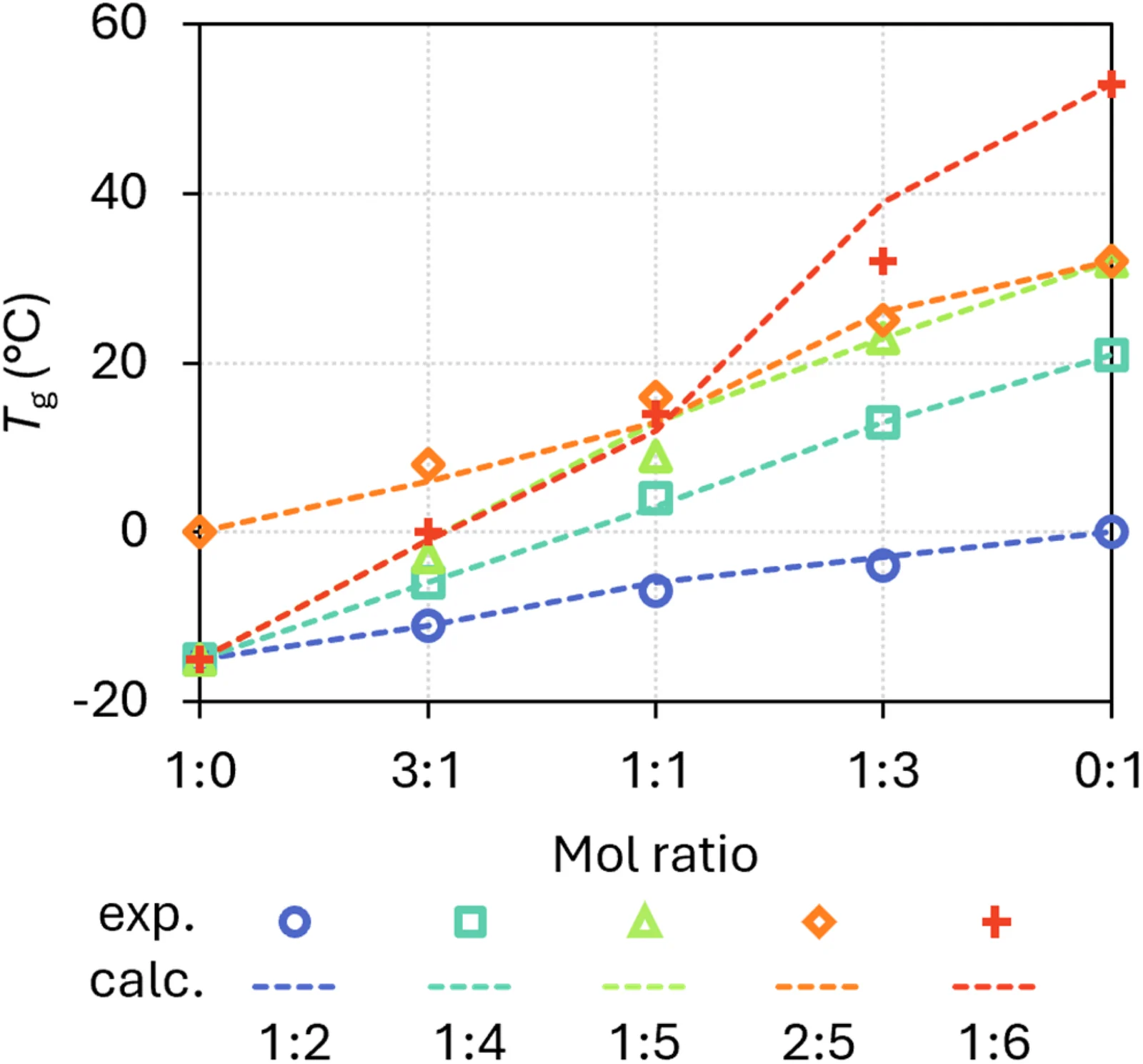
Glass transition temperatures of melt blends of 1, 2, and 4–6. DSC (fourth cycle, heat 10 °C min^−1^). Dashed lines calculated from Fox equation using weight fraction: 1/*T*_g_ = *w*_1_/*T*_g1_ + *w*_2_/*T*_g2_.

The broad range of *T*_g_'s of the class I glass formers 1–6 allowed us to investigate the production of melt-blended glasses at various molar ratios as shown in Fig. 4 (see also expanded data in Table S1). After melt-blending using DSC analysis followed by additional heating and cooling cycles, the *T*_g_'s revealed a linear relationship among the molar ratios of 1 : 0, 3 : 1, 1 : 1, 1 : 3, and 0 : 1 for two sets of selected compounds within the series 1–6 which illustrate the ability to obtain glasses with programmable glass transition temperatures. In addition, using the Fox equation for these melt miscible mixtures at varying weight percent concentration through the conversion of molar ratios, the theoretical *T*_g_'s match with the experimental values as determined by DSC. The ability to prepare these homogeneous composite molecular glasses provides a robust strategy for systematically shifting *T*_g_, which is essential for tailoring the relaxation dynamics of amorphous matrices. This programmatic control over the thermal profile establishes a foundational platform for their eventual application as functional host matrix materials, setting the stage for future investigations into their viscoelastic and mechanical properties.

### Class II glass formers: metastable glass formation of BTFPAEs 8 and 9

**Fig. 5:**
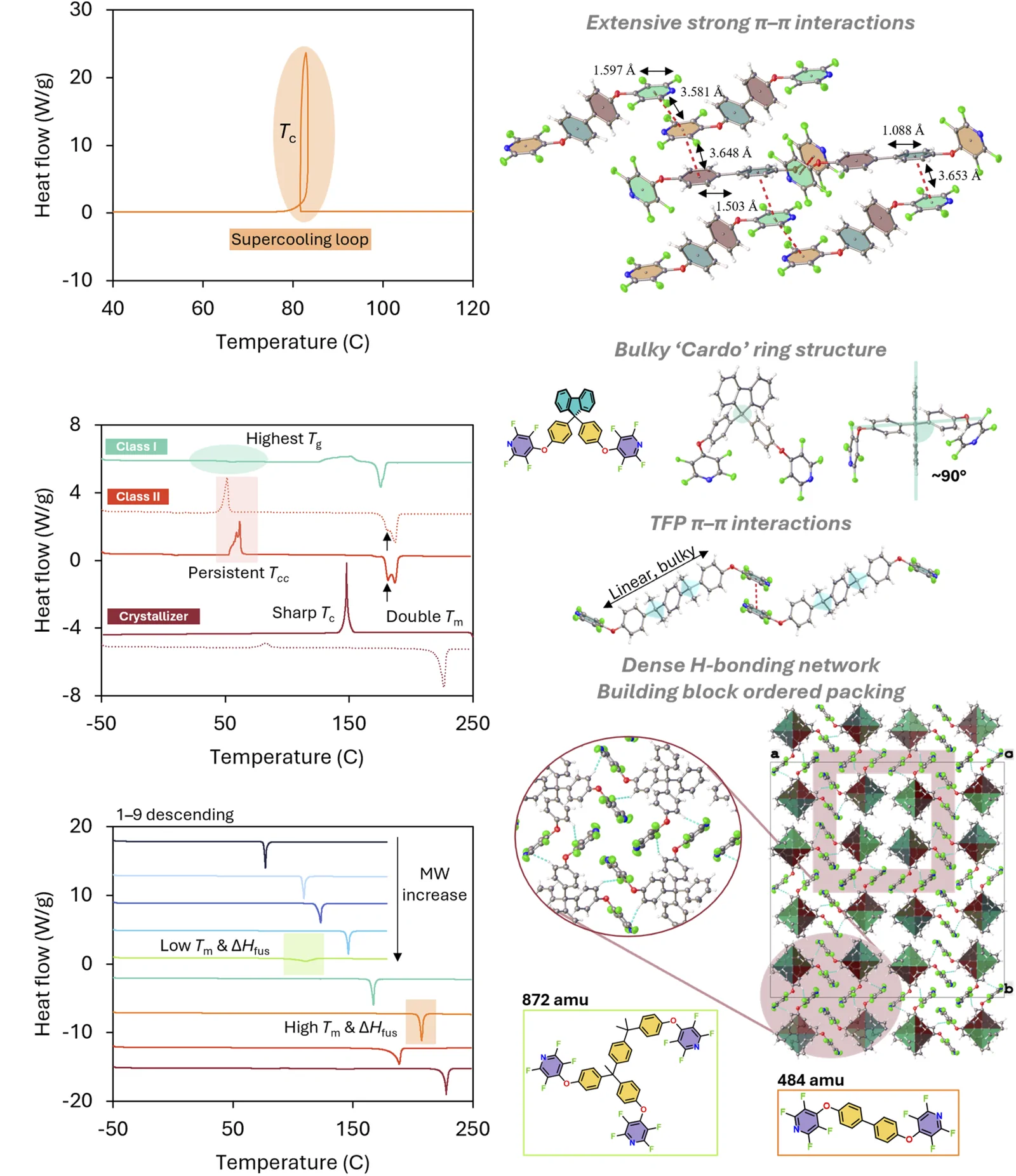
Thermal cycling behavior and correlations to crystal structure properties of selected BTFPAEs. Top DSC thermogram: close view of the crystallization event during cooling cycle of 7. Top right ORTEP: packing of 7 with π–π interactions and measured interaction distances. Middle thermogram: comparison of thermal behavior of 3 compound classes. Middle right: sterics of cardo pendant of 6 (top) and linear structure of 8 with TFP π–π interaction (bottom). Lower thermogram: overlay of melt transitions of 1–9. Lower ORTEP: crystal packing of the building block like structure of 9; zoomed view of dense hydrogen bonding network. Bottom right: color coded structures of 5 and 7 highlighting symmetry and molecular weight. All ORTEP representations with thermal ellipsoids at 50% probability.

As detailed in the previous sections, compounds 1–6 formed stable glasses over repeated heat/cool cycles with some complexity introduced by 2 and 6, which require multiple cycles before the cold-crystallization events disappear. Compound 8 is an ideal example of what we term a class II metastable glass former. Shown in Fig. 3 are three heat–cool cycles of DSC analysis for 8. After a slow cooling the compound glasses but then are followed by a *T*_cc_ and *T*_m_ (Table 1). This same behavior repeated after a rapid cooling cycle. Thus 8 is a glass former but it is found to be metastable because it spontaneously crystallizes when heat is applied and the system reaches the *T*_cc_. This behavior can be rationalized due to the unique linear structure of 8. Its size and rigid quaternized central carbon units add the rigidity necessary for vitrification and frustration of crystallization upon cooling. However, given sufficient molecular motion delivered *via* heating the molecules can spontaneously reorganize into a crystal lattice. This is in part driven by significant π–π stacking between the TFP rings on either end of the long central phenolic unit (Fig. 5). An additional unique feature of 8 is that it appears to have a double melt event after the first cold crystallization which subsequently gets more defined upon subsequent cycling. This suggests that there are two polymorphs of crystallinity. An attempt was made to extract X-ray quality crystals from the DSC pan, after the cold crystallization event but before the melt, to probe the multiple morphologies, but the attempt was unsuccessful due to the crystallization being rapid and affording a homogeneous microcrystalline substance unusable for single crystal X-ray crystallography. Compound 9 also forms a metastable glass, although not as readily as 8, placing it as a class II glass former. Unique among all the glass forming compounds in this work 9 has a true crystallization, *T*_c_, upon cooling. This crystallization is sharp and well defined and only possible to suppress with a rapid cooling rate (50 °C min^−1^).

### Class III non-glass forming: crystallization behavior of BTFPAEs 7

Compound 7 can be thought of as a control compound. Under all heat/cool cycling regimes 7 melted and then crystallized at *T*_c_ suddenly when cooled at any rate (see Fig. 3). The crystallization event is quite energetic and extremely rapid forming a loop trace in the DSC thermogram. This loop trace is indicative of super-cooling phenomenon. Analysis of the crystal structure of 7 sheds some light here as it shows three significant π–π interactions (Fig. 5). These interactions are afforded by the highly symmetrical and planar nature of 7 making rapid crystallization ideal. The small size of the molecule and high planar symmetry also precludes any frustration of these IMFs to trap in an amorphous vitreous form.

### Symmetry, rigidity, steric, and crystallographic property relationships to thermal cycling behavior of BTFPAEs 1–9

The geometry and functional elements of the central phenolic group as well as the overall symmetry of the TFP substitutions play a large role in determining the bulk phase behavior of the studied compounds. This can be readily observed by the diverse behavior of the compounds under various thermal cycling regimes. As detailed in previous sections, the compounds can be divided into three groups: class I, class II, and class III non-glass forming. Here we attempt to shed light on the molecular properties of each compound that lead to it falling into each class.

There are a few broad classes of symmetry in the compounds studied as summarized in Table 3 and expanded upon in Fig. 5. The symmetry of the glass forming compounds revolves around the central carbon of the central phenolic group. Most obviously there are dissymmetric and trisymmetric central phenolic groups as regards the TFP substitution pattern. The three trisymmetric compounds (3, 4, and 5) are all stable thermal glass formers of class I. The thermal data clearly shows a tendency for the trisymmetric compounds to vitrify upon slow cool (cooling rate independent to the limit of the rates tested in this study) resulting in a stable, permanent glass transition temperature. The crystal structure data of 3 and 4 show a visually chaotic packing arrangement compared to the more crystallization prone class II compounds. Both of these compounds have hydrogen bonding present with compound 4 possessing two hydrogen bonds in the lattice that are both involved in the two-part disorder with one of the TFP moieties (see Fig. 5). Compound 4 also has a π–π interaction through the TFP ring involved in the two-part disorder. While these IMFs would potentially be sufficient to induce a crystallization event (*T*_c_ or *T*_cc_), we hypothesize that the very large molecular volume (521 Å^3^Table 3) and the unwieldy trisymmetric nature of the structure contrive to frustrate any crystallization, instead favoring vitrification. Similar effects dominate for 3, which has even less IMFs to drive crystallization. Compound 5 is unique among the compounds in that it does not crystallize under any conditions thermally or from solvent evaporation. This is thought to be driven by the large, complex, bulky and non-symmetrical central phenolic group. Significantly, compound 5, the highest molecular weight, has a low *T*_m_ and a low Δ*H*_m_ (see Table 1 and Fig. 5, respectively), implying weak intermolecular forces in its microcrystalline powder form. This lack of significant IMFs is thought to be a partial driver of the difficulty in growing X-ray quality single crystals of compound 5. Future work could add further credence to these hypotheses through computational modeling of these compounds to obtain quantitative values for the IMFs in question. The other compound that falls completely outside the molecular weight trend is 7, the lowest molecular weight compound, which has the highest *T*_m_. Compound 7 is the most planar of the studied compounds and has highly regular packing arrangement of the linear aromatic ring structure affording a strong network of π–π interactions requiring a high energy input into the system to melt. The smallest two stable glass formers are 1 and 2 and consequently neither have any hydrogen bonding present in the crystal structure. The rigid angle of the central phenolic unit allows for easy disruption of the IMFs promoting crystallization allowing for vitrification at slow cooling rates.

**Table 3:** Selected relevant crystallographic properties for BTFPAEs 1–9

Entry	H-bond	Min. *F*–*F* (Å)	Tri *sym*	π–π	Cardo	mol SA (Å^2^)	mol V (Å^3^)	Mass (amu)
1	0	3.185	—	1	—	445	374	526
2	0	2.907	—	0	—	478	409	634
3	1	2.821	Y	0	—	595	508	739
4	2^a^	2.926	Y	2^b^	—	612	521	754
5	—	—	Y^c^	—	—	—	—	872
6	1^d^	2.714	—	0	Y	544	471	649
7	0	3.118	—	3	—	395	332	484
8	0	2.927	—	1	—	566	484	645
9	2	2.713	—	0	—	548	478	651

a Two additional H-bonds (totaling 4 for the structure) are present due to the minor component of a 2-part disorder in the crystal structure.

b The 2nd π–π interaction is present due to the 2-part disorder.

c Compound 5 has 3 TFP units but is not truly tri-symmetric about any axis.

d HTAB parameters non-standard: *D*–*A* distance 3.2 Å min. Angle 115.

Compound 6, the final class I glass former, is unique in its structure and thermal cycling behavior. The vitrification is driven by the bulky and sterically hindered fused ring cardo group (see ORTEP representation in Fig. 5). This bulky group sits nearly 90° from the axis of the dissymmetric molecule. This feature makes this compound ideal for glass formation (based on reasoning around the free volume theory of glass transitions) and, as such, 6 has by far the highest *T*_g_ (52 °C) of any of the compounds studied.

The designated class II glass formers 8 and 9, are more interesting and highly divergent in their thermal cycling behavior. Compound 8 has a long linear semi-rigid phenolic central unit. This structure is amenable to vitrification rather than crystallization upon cooling. However, a strong π–π interaction between the TFP pendant groups sets up the conditions for cold crystallization wherein the compound becomes semicrystalline. These IMFs along with the mobility of the long, linear, and symmetric central unit are postulated to be conducive to a stable and persistent *T*_cc_ across multiple thermal cycles. Also of note is the steady appearance of a double melt event with repeated thermal cycling of 8 and can be seen clearly in Fig. 5. It is believed that the specific geometry and sterics of the central unit, along with the strong π–π interaction between the TFP groups allow for two polymorphs to be energetically favorable. Future work to probe the distinct stable crystal polymorphs of the compounds will be to perform simultaneous DSC-XRD.

Compound 9 can be induced to glass upon rapid cooling. This vitreous component is, however, minor, as is the subsequent cold crystallization event. The proclivity for crystallization upon cooling displayed by 9 is postulated to be due to the dense network of strong hydrogen bonds the compound forms when crystallized, as well as the compact ‘building block’ like units the central phenolic group in the crystal structure as illustrated in Fig. 5. This postulation fits nicely within the overall theoretical framework revolving around IMFs, symmetry, and molecular volume as a complex set of variables governing the class type of glass former.

While variations in glass transition temperature and crystallization kinetics align with expected trends in molecular symmetry, π–π stacking potential, and internal rotational freedom, these structural mechanisms are proposed interpretations based on thermal behavior. In the absence of solid-state NMR or wide-angle X-ray scattering (WAXS) data to directly quantify molecular packing density and non-covalent interactions, these structural contributions remain speculative and warrant future crystallographic and computational modeling studies.

## Experimental

### General methods

All chemicals and solvents were obtained from commercial sources as reagent-grade and used as received. Glassware used for synthesis was flame- or oven-dried at 100 °C for 24 h. All reactions and solvent transfers were flushed and carried out under an atmosphere of nitrogen.

### Instrumentation

^1^H, ^13^C, and ^19^F NMR spectra were recorded on a JEOL 500 MHz spectrometer. Chemical shifts were reported in parts per million (ppm), and the residual solvent peak was used as an internal reference: proton (chloroform, *δ* 7.26 ppm), carbon (chloroform, C{D} triplet, *δ* 77.0 ppm), and fluorine (CFCl_3_, *δ* 0.00 ppm) was used as a reference. Data are reported as follows: chemical shift, multiplicity (s = singlet, d = doublet, t = triplet, m = multiplet with reported range or at midpoint), coupling constants (Hz) and integration.

Gas chromatography mass spectrometry (GC-MS) analyses were performed on an Agilent 8890 gas chromatography with H_2_ carrier gas coupled to an Agilent 5977C electron impact mass spectrometer with initial injection temperature of 250 °C and a temperature gradient of 100 °C to 325 °C at 20 °C min^−1^.

For thermal analysis, samples were dried in a vacuum oven (20 mm Hg) at 40 °C for 24 h prior to use. Differential scanning calorimetry (DSC) analysis was performed on a TA 2500 DSC utilizing Tzero aluminum pans and lids. Thermal analysis was carried out using a temperature gradient in nitrogen and specified cooling–heating cycles reported in Tables 1, 2 and Fig. 4 footnotes. Glass transition temperatures (*T*_g_) were determined from the midpoint of the inflection tangent of the endothermic step transition, while melting point temperatures (*T*_m_) were identified as the peak maximum of the melting endotherm. All thermal events were integrated and evaluated using the TA Trios software graphical suite.

Thermogravimetric analysis (TGA) was performed on a TA 5500 utilizing platinum pans at 10 °C min^−1^ temperature gradient to 500 °C in nitrogen. Onset of thermal decomposition (*T*_d_) and char yield was reported using TA Trios software package. TGA analysis of compounds 1–6 involved initially glassing them using DSC heating–cooling cycles to induce *T*_g_, then the amorphous samples (tared with Tzero aluminum pan) were then measured using TGA. All reported measurements of *T*_m_ and *T*_g_ have been performed at least in triplicate and are within ± 0.6 °C standard deviation.

Single crystal X-ray diffraction studies were carried out on a Rigaku XtaLAB synergy, Dualflex, HyPix3000 diffractometer equipped with a Cu K_α_ radiation source (*λ* = 1.542 Å). Suitable crystals were selected and mounted on a Cryoloop secured with Paratone-N oil. The crystal of interest was kept at a steady *T* = 100.0(3) K in a nitrogen gas stream during data collection. Crystal-to-detector distance was 40.6 mm using an exposure time of 0.2 seconds with a scan width of 0.50° for all crystals in this work. X-ray quality crystals of all compounds were obtained *via* slow evaporation from an appropriate solvent. The data collection routine, unit cell refinement, and data processing were carried out with the program CrysAlisPro (version 43.91a) and scaled using an empirical absorption correction implemented in the SCALE 3 ABSPACK software program as well as a numerical absorption correction based on Gaussian integration over a multifaceted crystal model. The structure was solved using ShelXT 2018/2 (ref. 53) and refined using ShelXL 2019/3 (ref. 54) by least squares minimization *via* Olex2.^55^ The final refinement model involved anisotropic displacement parameters for non-hydrogen atoms and a riding model for all hydrogen atoms. Olex2, Mercury, and ORTEP3 were used for molecular graphics generation.

### General procedure for the synthesis of BTFPAE compounds 1–9

For 5-gram scale, cesium carbonate (0.5 mol% excess), pentafluoropyridine (0.25 mol% excess), and designated phenolic material (1 equiv.) (4,4′,4″-trihydroxytriphenylmethane for 3; 4,4′-(1-(4-(2-(4-hydroxyphenyl)propan-2-yl)phenyl)ethane-1,1-diyl)diphenol for 5; 4,4′-(9*H*-fluorene-9,9-diyl)diphenol for 6; 4,4′-dihydroxybiphenyl for 7; 4,4′-(1,4-phenylenediisopropylidene)bisphenol for 8; 4,4-dihydroxytetraphenylmethane for 9) were combined in acetonitrile (100 mL) and allowed to stir for 24 h at room temperature. The reaction was monitored by ^19^F NMR until 100% conversion of the desired product was observed. The solution was vacuum filtered to remove carbonate salts and washed copiously with acetonitrile. The filtrate was then concentrated using rotary evaporation and dried under high vacuum manifold (2–5 mm Hg). Recrystallization was accomplished by dissolving crude solids with hot 95% ethanol and upon slow cooling to room temperature afforded the titled compound after vacuum filtration and drying under high vacuum manifold (2–5 mm Hg). Compounds 1, 2, and 4 were synthesized and characterized from previously published work.^49^

#### Tris(4-((perfluoropyridin-4-yl)oxy)phenyl)methane (3)

Yield: 98% (white semi-crystalline solid). ^1^H NMR (CDCl_3_, 500 MHz) *δ* 7.10 (d, *J* = 8.45 Hz, 6H), 7.02 (d, *J* = 8.45 Hz, 6H), 5.57 (s, 1H); ^13^C{^1^H} NMR (CDCl_3_, 126 MHz) *δ* 154.6, 144.2 (dm, *J* = 245 Hz), 144.4 (m), 140.1, 135.6 (dm, *J* = 245 Hz), 130.1, 116.9, 54.4; ^19^F NMR (CDCl_3_, 471 MHz) *δ* −88.3–(−88.4) (m, 6F), −154.0–(−154.2) (m, 6F); GC-MS (70 eV) *m*/*z* (% relative intensity) 739.3 ([M]^+^, 15), 573.2 (60), 497.1 (50), 330.1 (50), 165.1 (100).

#### 4,4′-(((1-(4-(2-(4-((Perfluoropyridin-4-yl)oxy)phenyl)propan-2-yl)phenyl)ethane-1,1-diyl)bis-(4,1-phenylene))bis(oxy))bis(2,3,5,6-tetrafluoropyridine) (5)

Yield: 86% (white semi-crystalline solid). ^1^H NMR (CDCl_3_, 500 MHz) *δ* 7.23 (dm, *J* = 8.88 Hz, 2H), 7.13 (d, *J* = 8.45 Hz, 2H), 7.07 (d, *J* = 8.88 Hz, 4H), 6.94 (dm, *J* = 8.59 Hz, 8H), 2.15 (3H), 1.66 (6H); ^13^C{^1^H} NMR (CDCl_3_, 126 MHz) *δ* 154.1, 148.3, 147.6, 145.8, 145.6, 144.5 (m), 144.5 (dm, *J* = 245 Hz), 136.5 (dm, *J* = 245 Hz), 130.3, 128.4, 128.2, 126.5, 116.2, 116.1, 51.5, 42.4, 30.9, 30.7; ^19^F NMR (CDCl_3_, 471 MHz) *δ* −88.3–(−88.7) (m, 6F), −154.0–(−154.5) (m, 6F); GC-MS (70 eV) *m*/*z* (% relative intensity) 856.3 ([M]^+^, 30), 420.6 (30), 284.1 (75), 256.0 (90), 207.0 (100), 178.0 (80), 133.0 (75), 102.0 (70), 77.1 (75).

#### 4,4′-(((9*H*-Fluorene-9,9-diyl)bis(4,1-phenylene))bis(oxy))bis(2,3,5,6-tetrafluoropyridine) (6)

Yield: 80% (white semi-crystalline solid). ^1^H NMR (CDCl_3_, 500 MHz) *δ* 7.70 (d, *J* = 7.59 Hz, 2H), 7.40–7.34 (overlapping m, 4H), 7.30–7.28 (m, 2H), 7.18 (dm, *J* = 8.88 Hz, 4H), 6.90 (dm, *J* = 8.88, 4H); ^13^C{^1^H} NMR (CDCl_3_, 126 MHz) *δ* 154.7, 150.5, 144.5–135.0 (unresolved multiplicity), 142.6, 140.1, 129.7, 128,09, 128.03, 126.0, 120.5, 116.5, 64.3; ^19^F NMR (CDCl_3_, 471 MHz) *δ* −88.2–(−88.4) (m, 4F), −153.8–(−154.3) (m, 4F); GC-MS (70 eV) *m*/*z* (% relative intensity) 648.3 ([M]^+^, 100), 482.2 (70), 406.2 (80), 239.1 (90).

#### 4,4′-Bis((perfluoropyridin-4-yl)oxy)-1,1′-biphenyl (7)

Yield: 85% (white crystalline solid). ^1^H NMR (THF-d_8_, 500 MHz) *δ* 7.63 (d, *J* = 8.7 Hz, 4H), 7.23 (d, *J* = 8.7 Hz, 4H); ^13^C{^1^H} NMR (THF-d_8_, 126 MHz) *δ* 155.8, 144.1 (m), 144.5 (dm, *J* = 245 Hz), 136.9, 136.4 (dm, *J* = 262), 128.4, 116.8, 124.4; ^19^F NMR (THF-d_8_, 471 MHz) *δ* −91.2–(−91.3) (m, 4F), −156.0–(−156.1) (m, 4F); GC-MS (70 eV) *m*/*z* (% relative intensity) 484.1 ([M]^+^, 80), 334 (50), 306.1, (85), 152.1 (100), 139.1 (90).

#### 1,4-Bis(2-(4-((perfluoropyridin-4-yl)oxy)phenyl)propan-2-yl)benzene (8)

Yield: 75% (white powder). ^1^H NMR (CDCl_3_, 500 MHz) *δ* 7.21 (d, *J* = 8.73 Hz, 4H), 7.09 (s, 4H), 6.93 (d, *J* = 8.73 Hz, 4H), 1.67 (12H); ^13^C{^1^H} NMR (CDCl_3_, 126 MHz) *δ* 153.8, 147.8, 147.6, 144.5–135.0 (unresolved multiplicity), 128.4, 126.4, 115.1, 42.3, 30.9; ^19^F NMR (CDCl_3_, 471 MHz) *δ* −88.3–(−88.7) (m, 4F), −154.1–(−154.6) (m, 4F); GC-MS (70 eV) *m*/*z* (% relative intensity) 644.3 ([M]^+^, 15), 629.2 (100), 307.1, (80), 284.1 (80), 256.1 (75), 178.1 (50), 102.1 (50).

#### Bis(4-((perfluoropyridin-4-yl)oxy)phenyl)diphenylmethane (9)

Yield: 90% (white semi-crystalline solid). ^1^H NMR (DMSO-*d*_6_, 500 MHz) *δ* 7.30 (m, 8H), 7.20 (m, 6H), 6.93 (m, 4H); ^13^C{^1^H} NMR (DMSO-*d*_6_, 126 MHz) *δ* 154.1, 146.4, 147.6, 144.0 (dm, *J* = 245 Hz), 143.0 (m), 136.8 (dm, *J* = 245 Hz), 132.5, 130.8, 128.5, 127.5, 116.2, 64.0; ^19^F NMR (DMSO-*d*_6_, 471 MHz) *δ* −88.3–(−88.7) (m, 4F), −154.1–(−154.6) (m, 4F); GC-MS (70 eV) *m*/*z* (% relative intensity) 650.2 ([M]^+^, 15), 573.1 (75), 408.1 (30), 253.0 (55), 241.1 (60), 207.0 (75), 165.1 (100) 152.0 (30), 73 (30).

## Conclusion

This study successfully demonstrates the synthesis and characterization of a novel class of amorphous molecular organic glass formers derived from pentafluoropyridine (PFP). We prepared a series of bistetrafluoropyridine aryl ethers (BTFPAEs) through a single, high-yielding, and scalable nucleophilic aromatic substitution reaction using PFP and commercially available bisphenols. The resulting compounds exhibit the required structural features: high molecular weight, molecular asymmetry, and numerous aromatic rings, to form stable glassy states upon melting and cooling. By varying the central phenolic architecture, we successfully mapped a highly nuanced structure–property relationship that governs small-molecule vitrification, categorizing the materials into predictable thermodynamic profiles. Thermal analysis revealed two distinct classes of glass-forming behavior among the synthesized BTFPAEs. Compounds 1–6 were identified as class I glass formers. Upon initial melting, these compounds underwent an irreversible transition to a transparent, amorphous glass. Their high molecular weight and molecular entanglement prevent recrystallization, leading to well-defined glass transition temperatures (*T*_g_) ranging from −15 °C to 53 °C. The ability to program the *T*_g_ was further demonstrated by preparing melt-blended glasses from these compounds, with the resulting *T*_g_ values showing a linear relationship with the molar ratios, consistent with the Fox equation. This tunability highlights their potential for various material applications. Conversely, compounds 8 and 9 were classified as class II glass formers or metastable-glass formers. When the class II molecules were induced to glass this state was metastable with a cold crystallization occurring. The specific thermal cycling behavior in class II is complex and correlated to specific intermolecular forces identified in the crystal structures of those compounds. Lastly, only two of the studied compounds (7 and 9) displayed a crystallization event (*T*_c_) which could not be suppressed upon rapid quench cooling. The *T*_c_ was driven in large part by compact stackable geometry and crystal structures patterned by strong IMFs including hydrogen bonding and π–π interaction networks. This work confirms the significant influence of molecular symmetry, flexibility, and the potential intermolecular forces in the crystal structure on the thermal behavior of these materials. The interplay between these properties is complex and leads to a nuanced structure–property relationship determining thermal behavior and molecular glass stability, thus opening the door to a novel molecular glass matrix platform which has a versatility and tunability useful in further materials development.

Future research directions will focus on expanding the monomer library to incorporate non-symmetrical, multi-functionalized phenolic cores to further frustrate packing geometry and suppress crystallization tendencies. Additionally, while this study establishes the necessary thermodynamic and structural foundations for *T*_g_ programmability, future work must evaluate the thin-film mechanics, viscoelastic profiles, and optical clarity of these systems. Characterizing these physical and mechanical properties will be essential to transition these tunable molecular glass matrices into functional host environments for advanced materials applications.

## Author contributions

Nathan J. Weeks: original draft, conceptualization, formal analysis. Anika A. Speicher: investigation, formal analysis. Scott T. Iacono: original draft, review & editing, supervision, conceptualization, funding acquisition. All authors have read and agreed to the published version of the manuscript.

## Conflicts of interest

The authors declare no competing financial interest.

## Supplementary Material

RA-OLF-D6RA07696C-s001

RA-OLF-D6RA07696C-s002

## Data Availability

CCDC 2328011 (for 1), 2254308 (for 2), 2483251 (for 3), 2328412 (for 4), 2486388 (for 6), 2491516 (for 7), 2483195 (for 8) and 2504217 (for 9) contain the supplementary crystallographic data for this paper.^56*a*–*h*^ All data supporting the findings of this study are available within the article and its supplementary information (SI). Supplementary information: NMR spectra, GCMS, DSC, and TGA analysis of all the synthesized compounds. See DOI: https://doi.org/10.1039/d6ra07696c.
