# Supplementary material for: Molecular engineering of bistetrafluoropyridine aryl ethers: a general platform for stable and programmable amorphous organic solids
Source: RSC Adv. 2026 Sep 18;16(44):54589–601. doi: 10.1039/d6ra07696c (PMC13588123; doi:10.1039/d6ra07696c)
Supplement: Supplementary file 1 [file RA-016-D6RA07696C-s001.pdf]

Supplementary Information (SI) for RSC Advances.
This journal is © The Royal Society of Chemistry 2026

SUPPORTING INFORMATION

Molecular Engineering of Bistetrafluoropyridine Aryl Ethers: A General Platform for Stable and Programmable Amorphous Organic Solids

Nathan J. Weeks, Anika A. Speicher, Scott T. Iacono**

[a]Department of Chemistry and Chemistry Research Center, Laboratories for Advanced Materials, United States Air Force Academy, Colorado Springs, Colorado (USA)

*Co-corresponding author: scott.iacono@afacademy.af.edu
*Co-corresponding author: nathan.weeks@afacademy.af.edu

Table of Contents

NMR Data S2
GCMS Data S20
Thermal Analysis (DSC & TGA Data) S23
Crystallographic Data S32

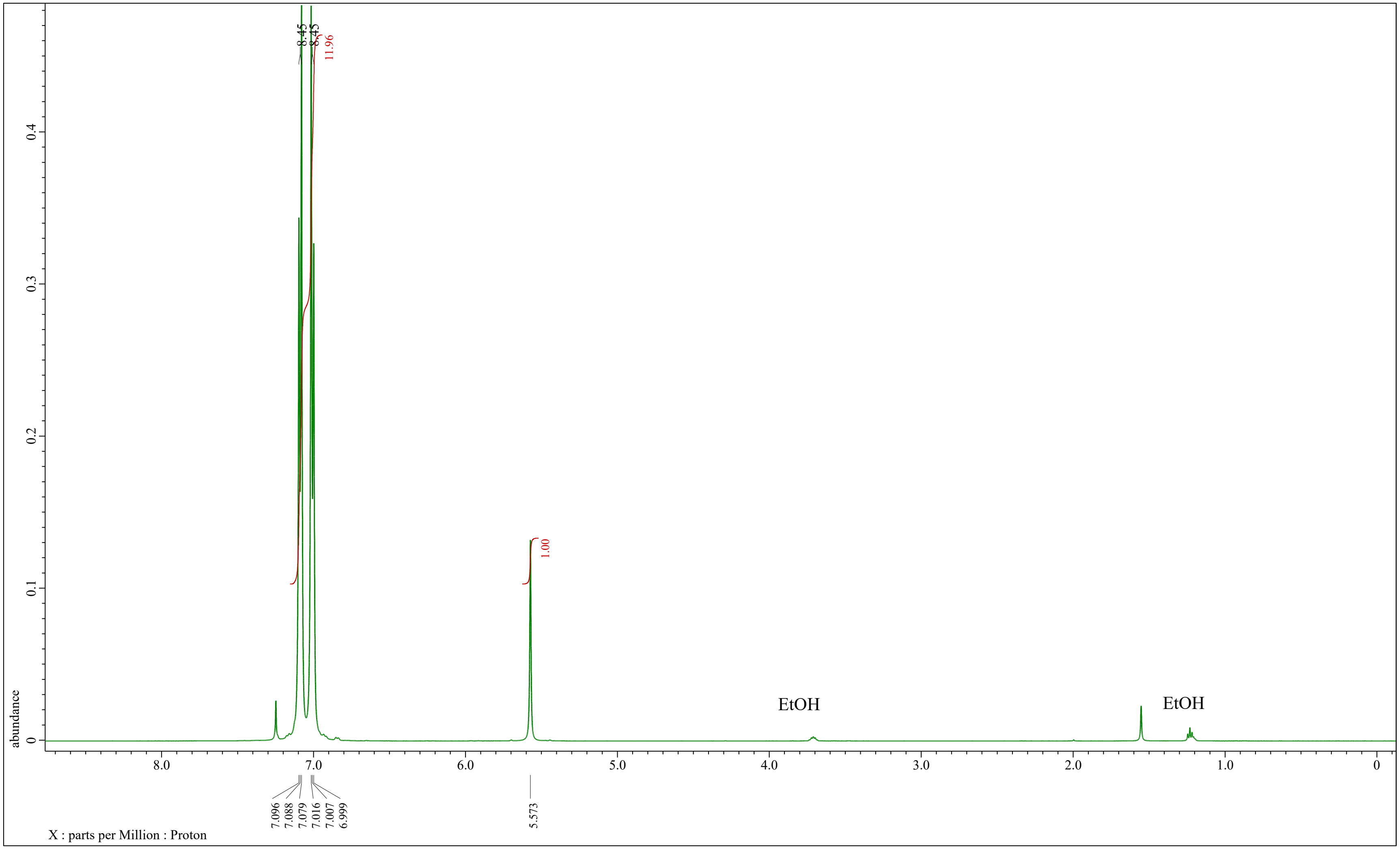

Figure S1. ^{1}H NMR of **3**.

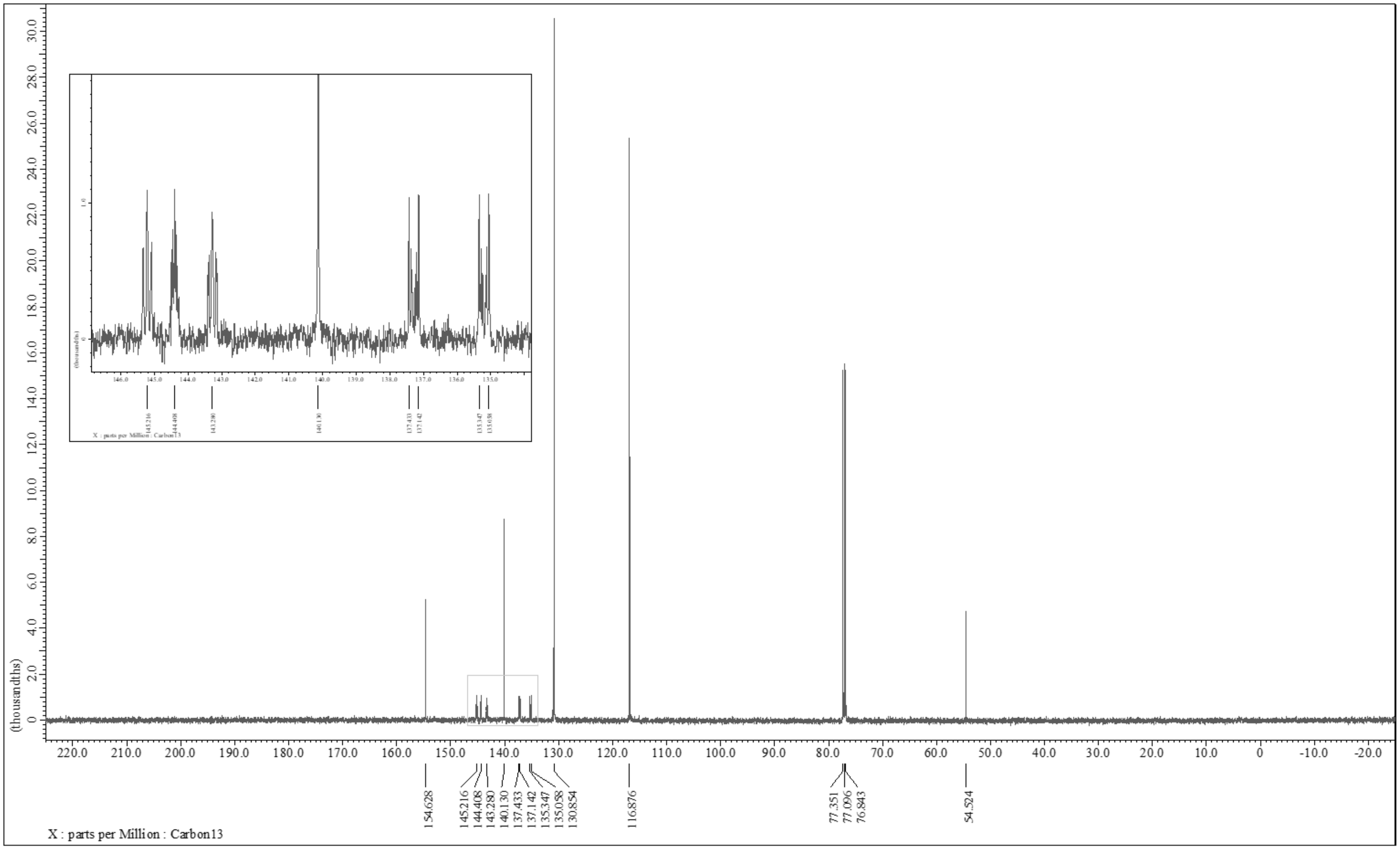

Figure S2. ^{13}C NMR of **3**.

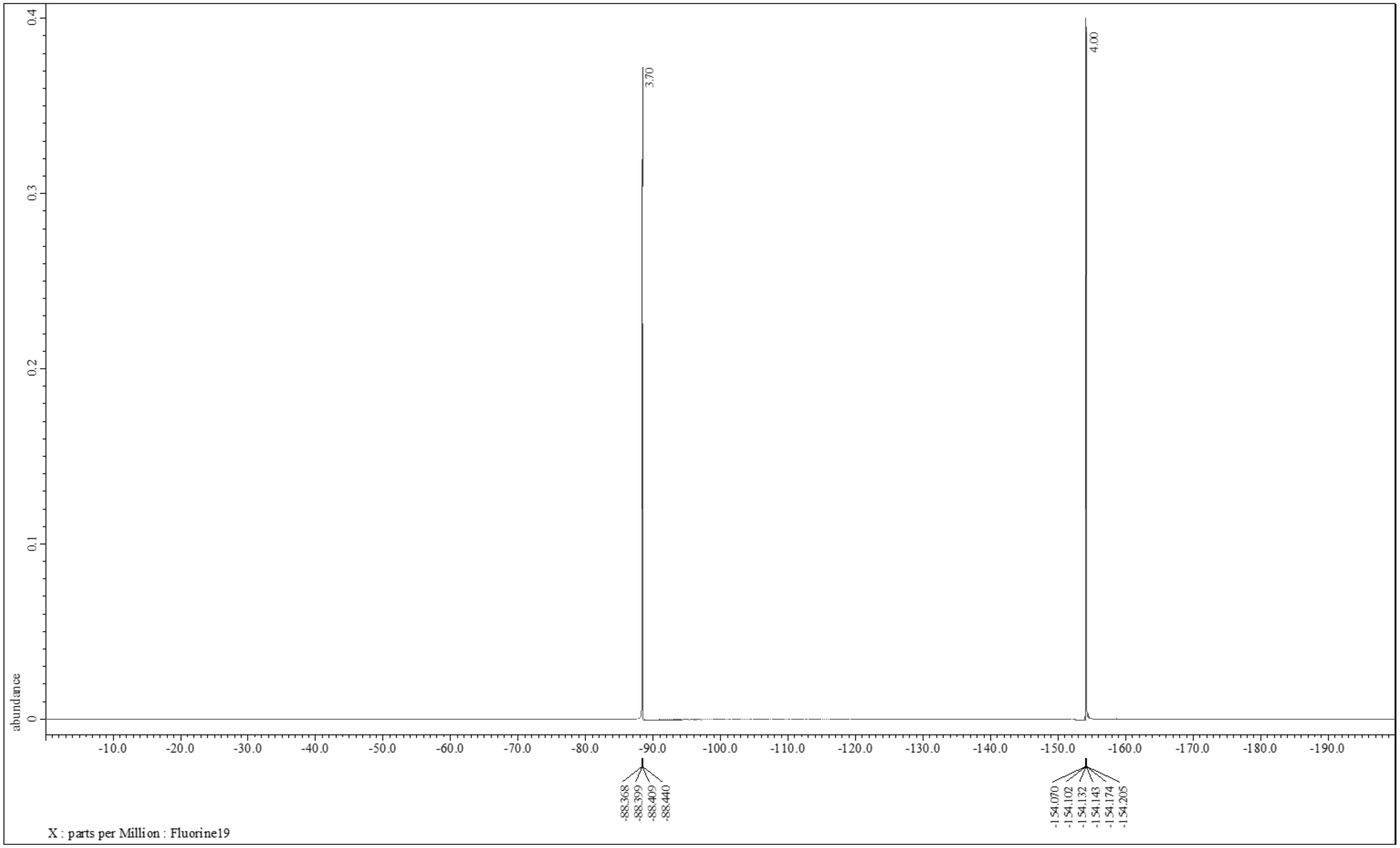

Figure S3. ^{19}F NMR of **3**.

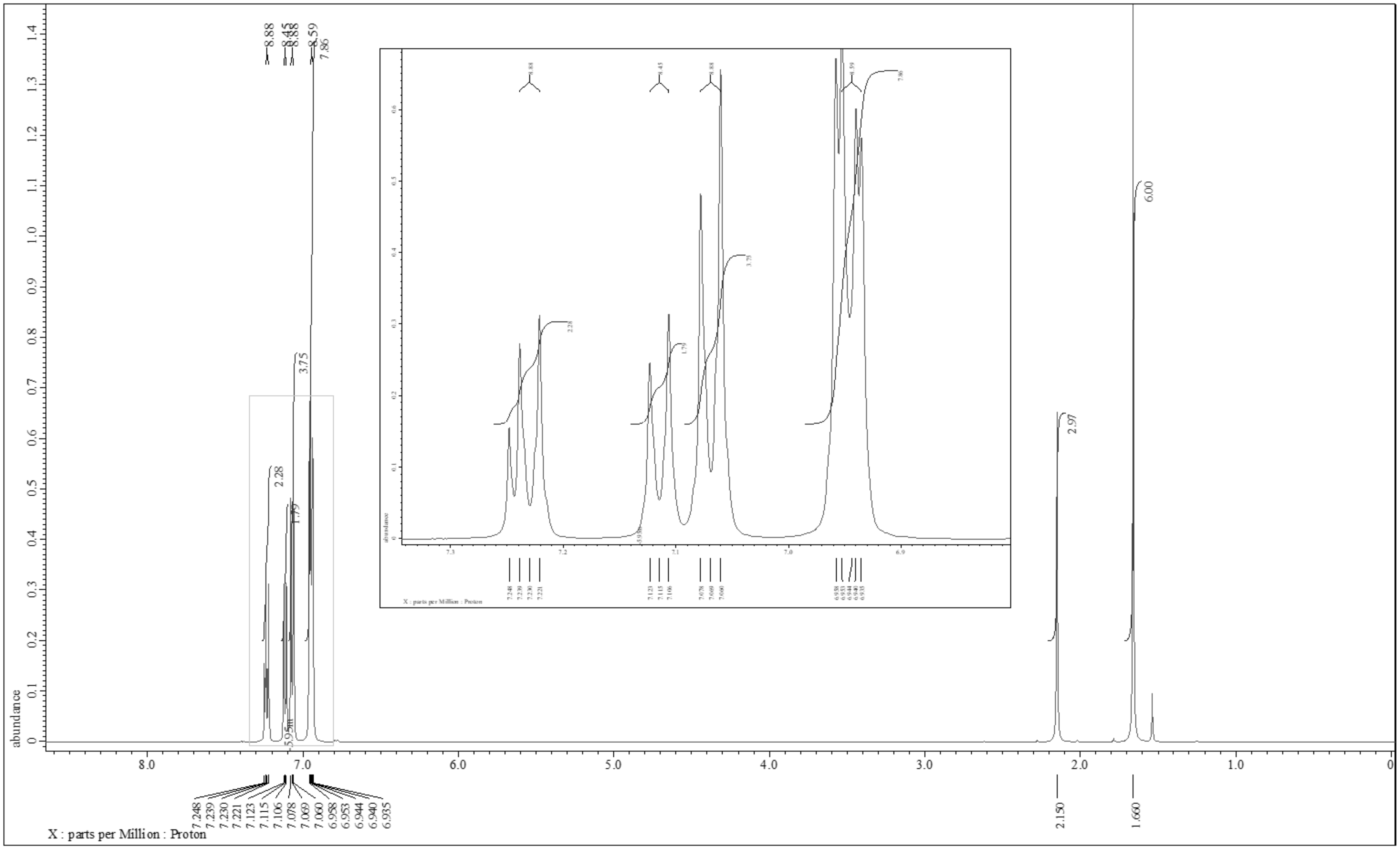

Figure S4. ^{1}H NMR of **5**.

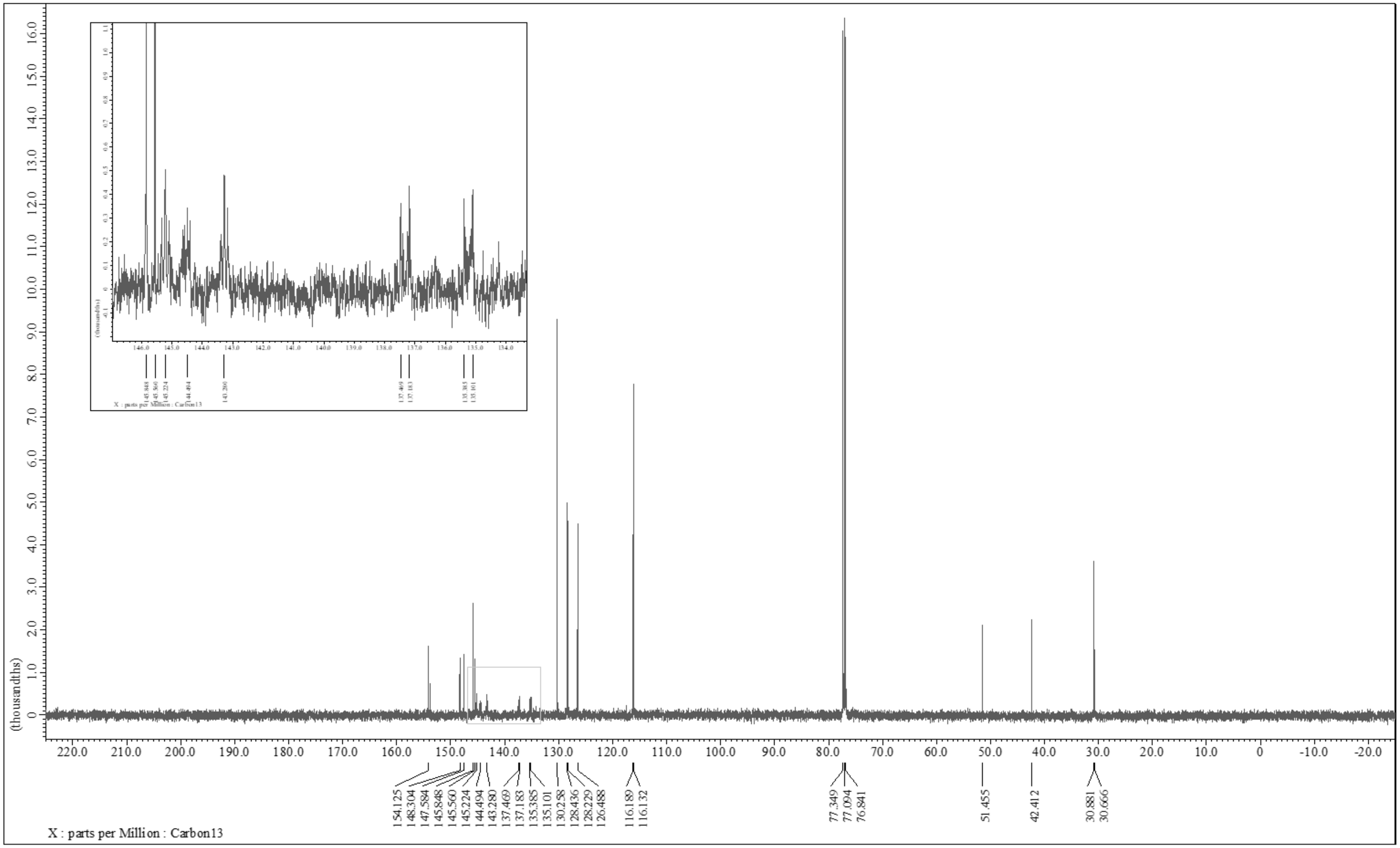

Figure S5. ^{13}C NMR of **5**.

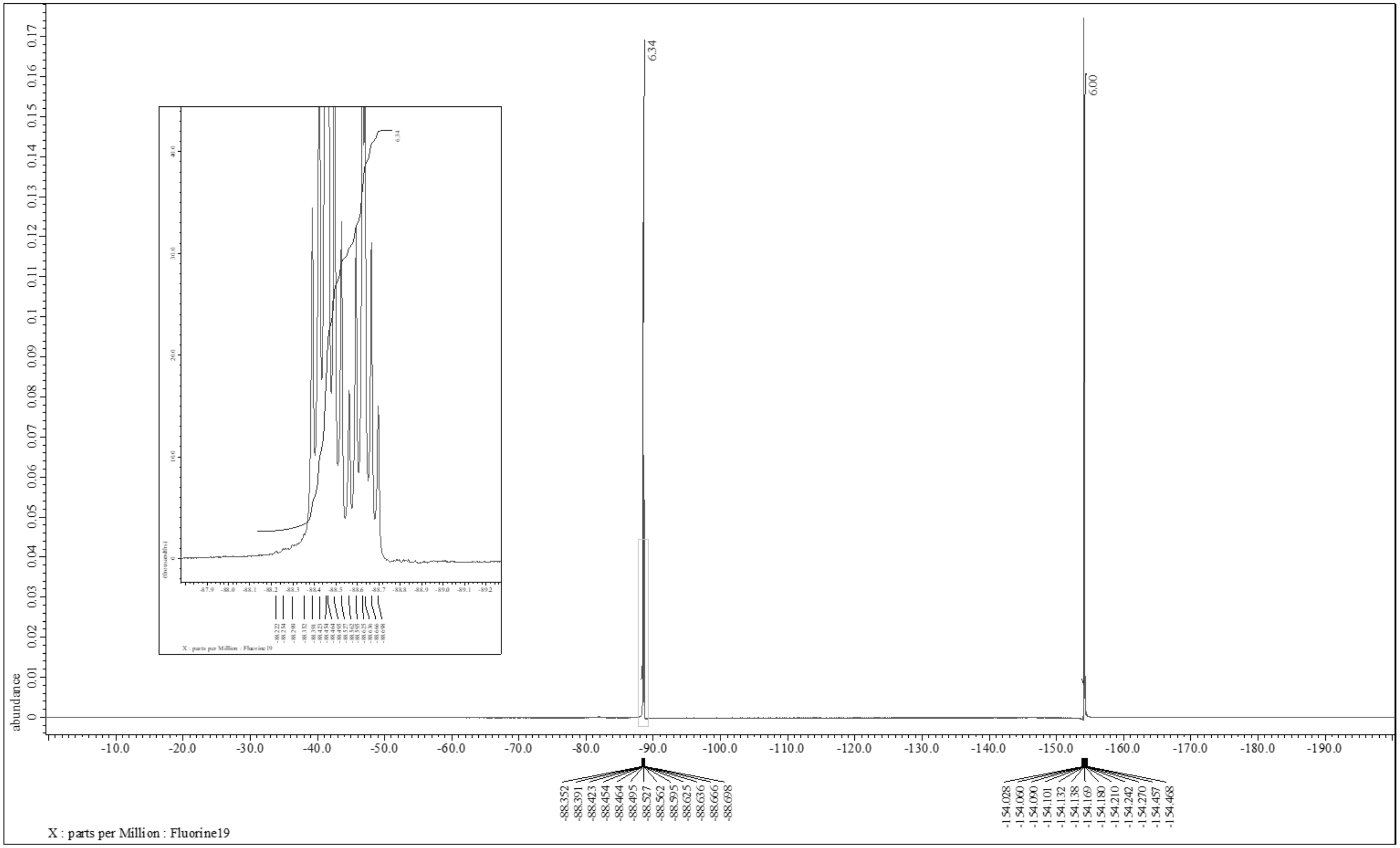

Figure S6. ^{19}F NMR of **5**.

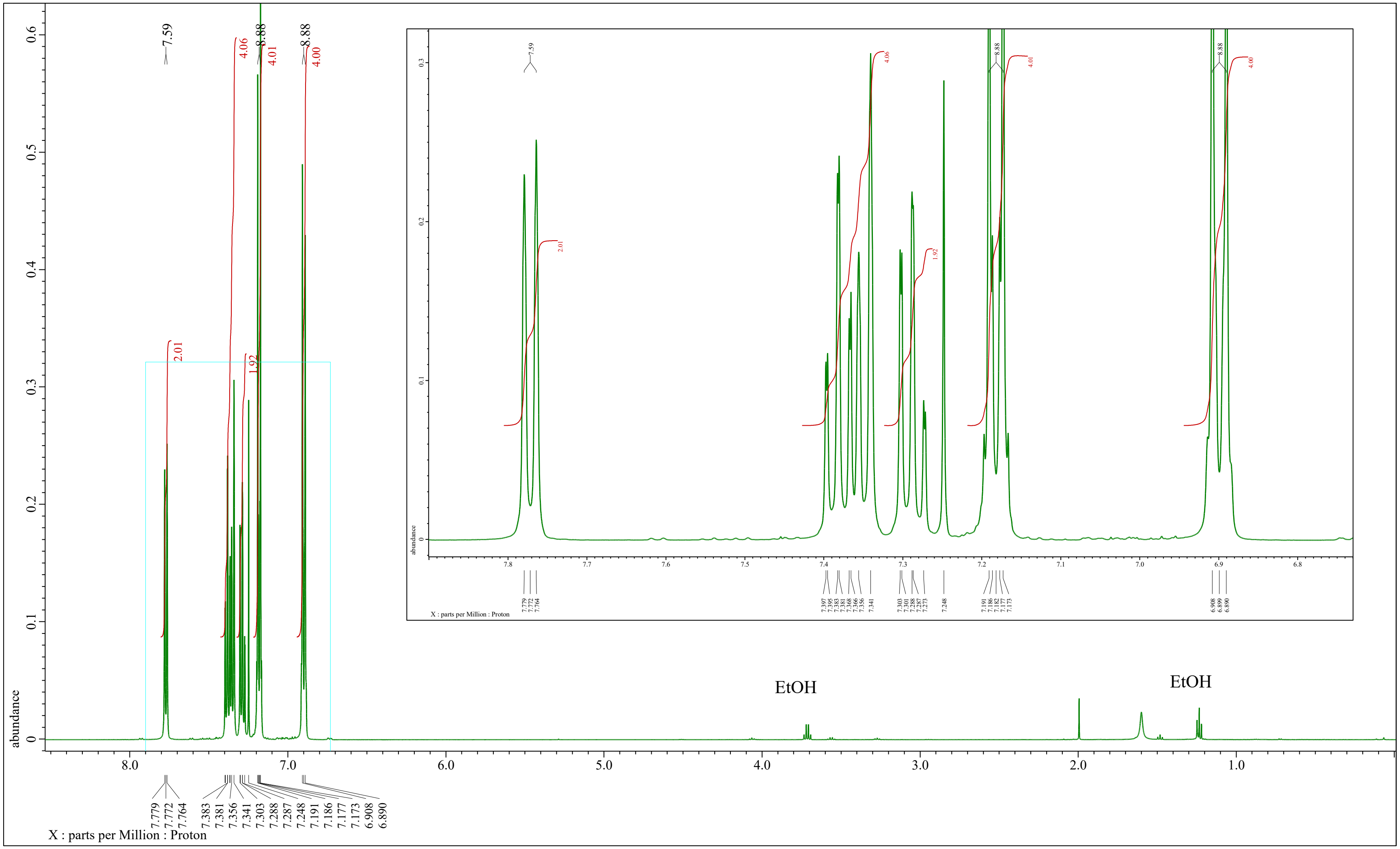

Figure S7. ^{1}H NMR of **6**.

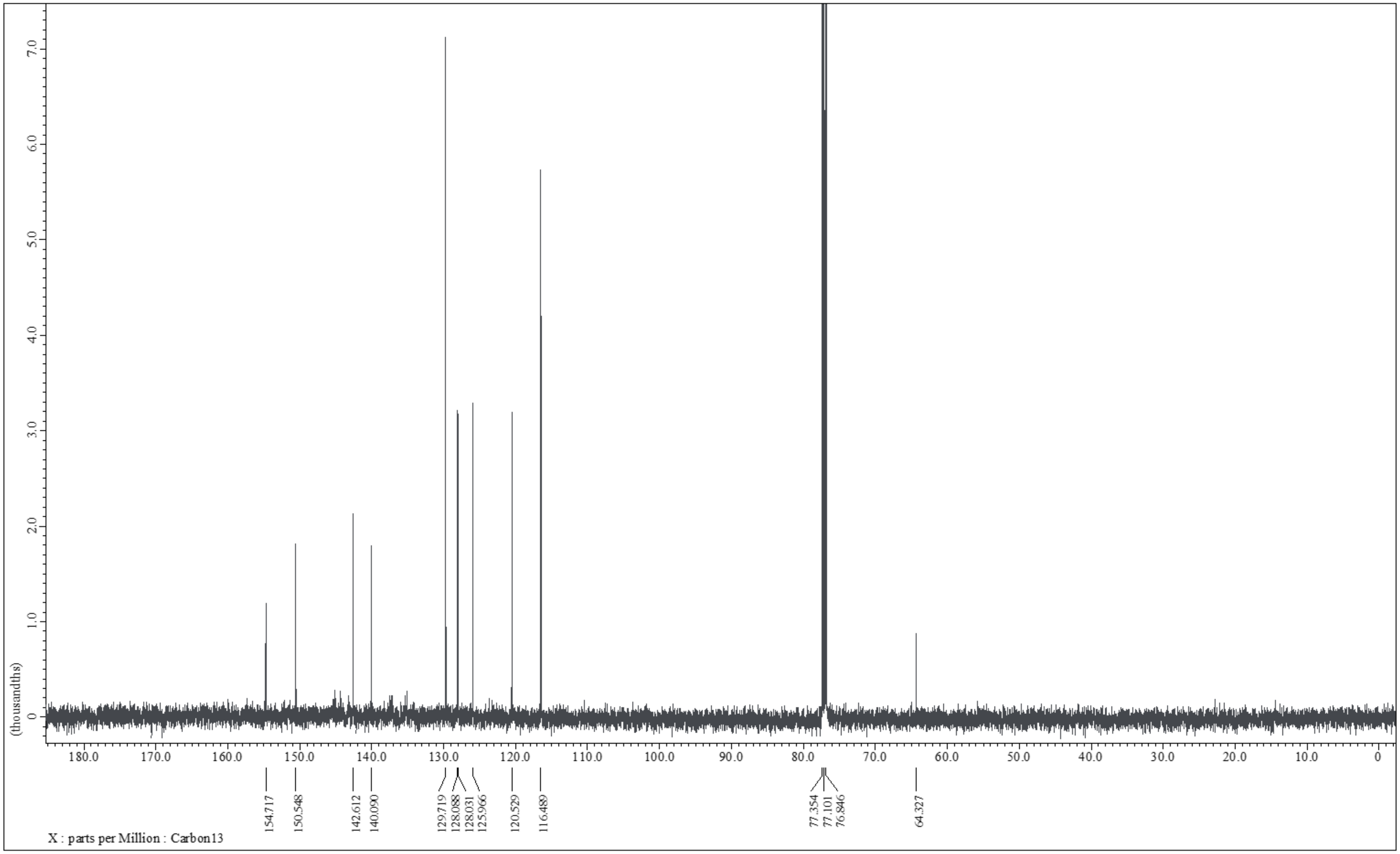

Figure S8. ^{13}C NMR of **6**.

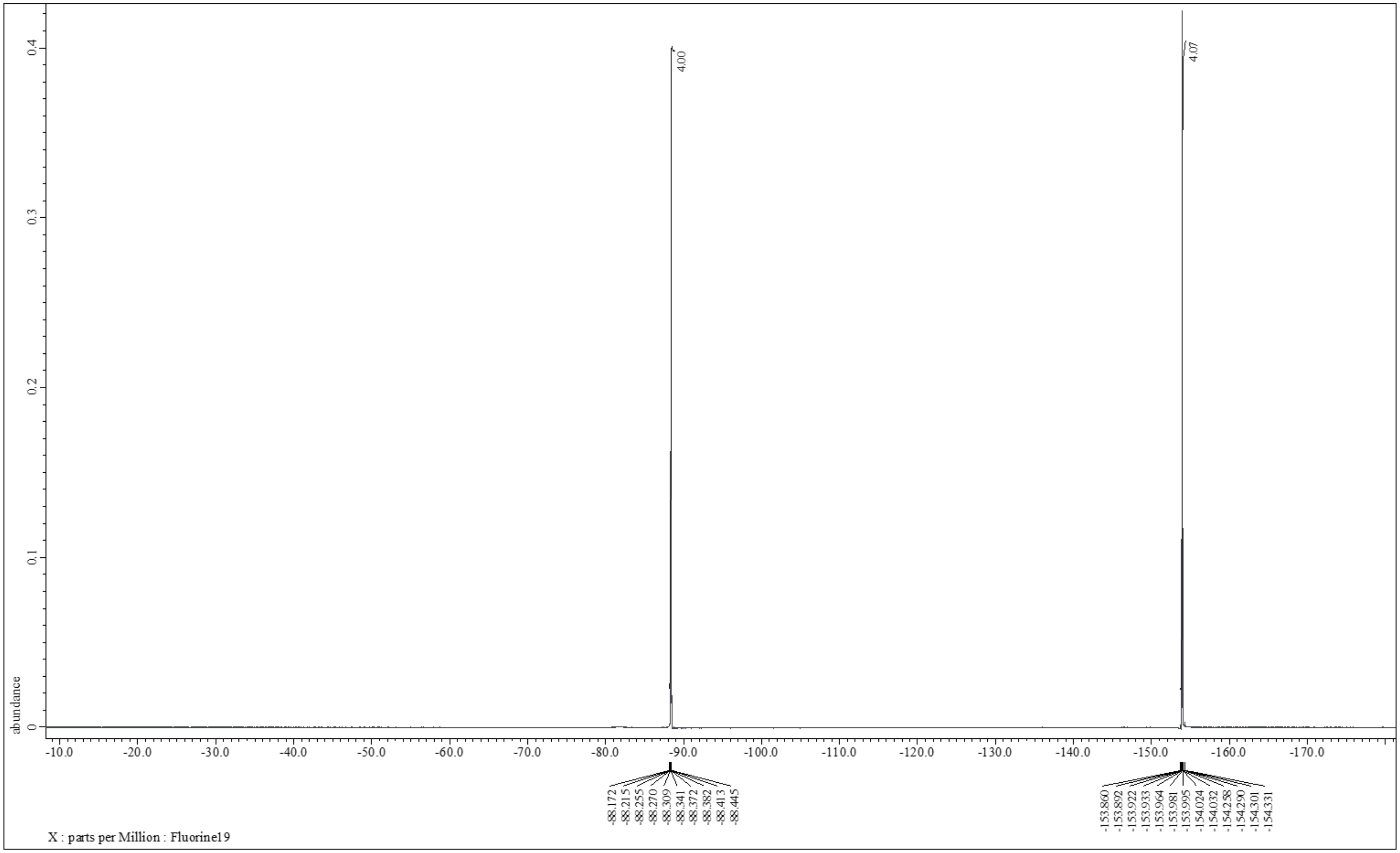

Figure S9. ^{19}F NMR of **6**.

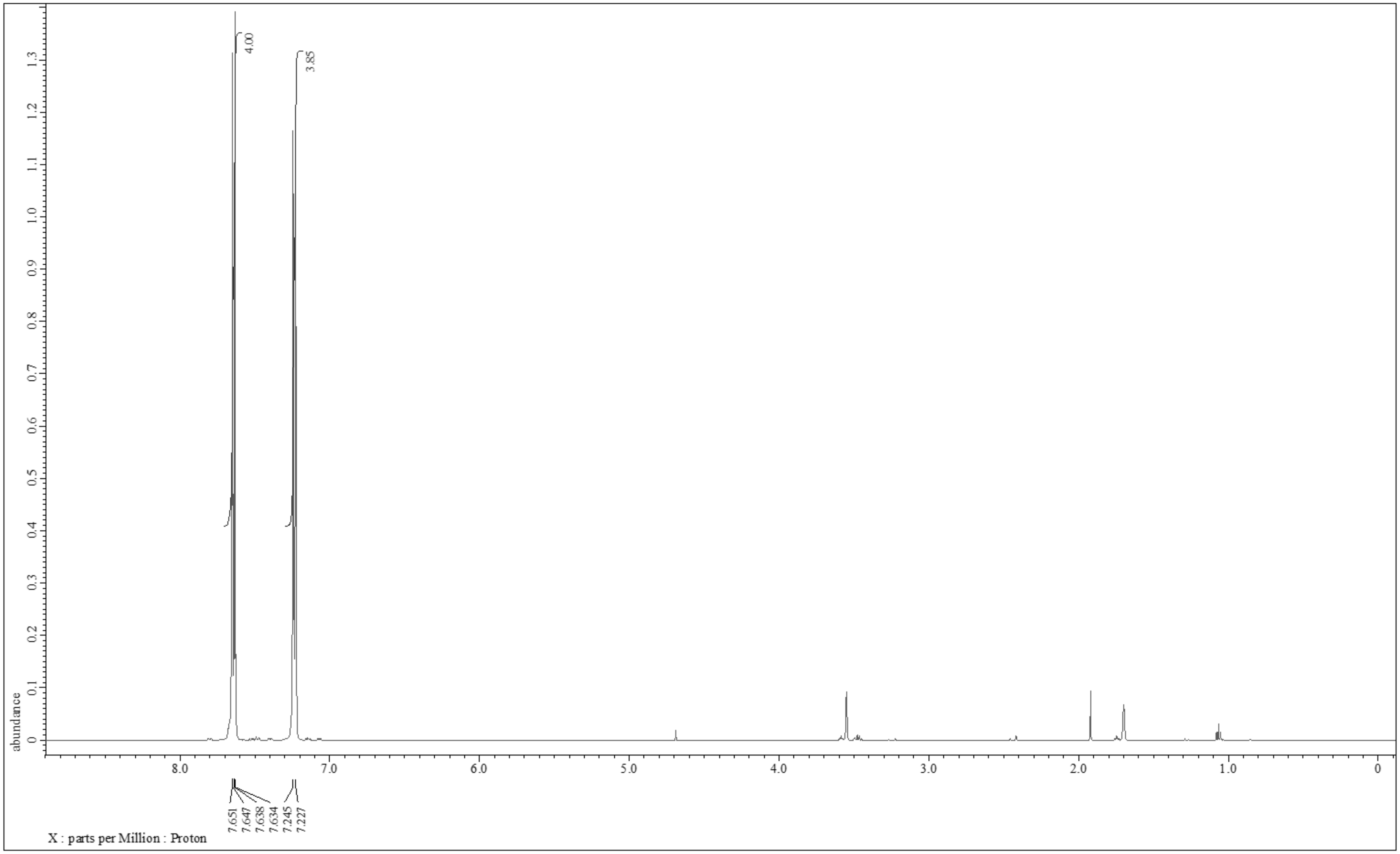

Figure S10. ^{1}H NMR of **7**.

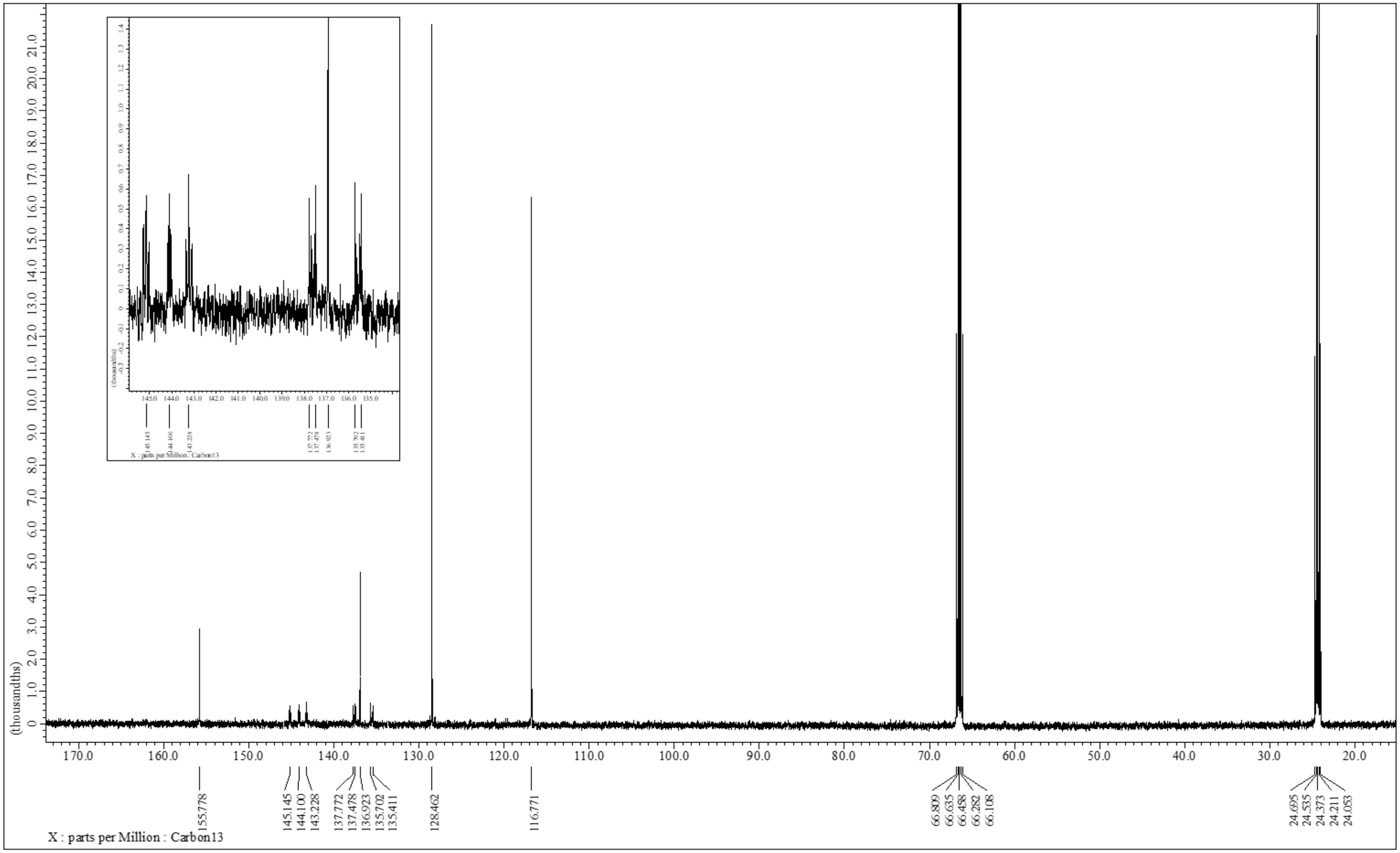

Figure S11. ^{13}C NMR of **7**.

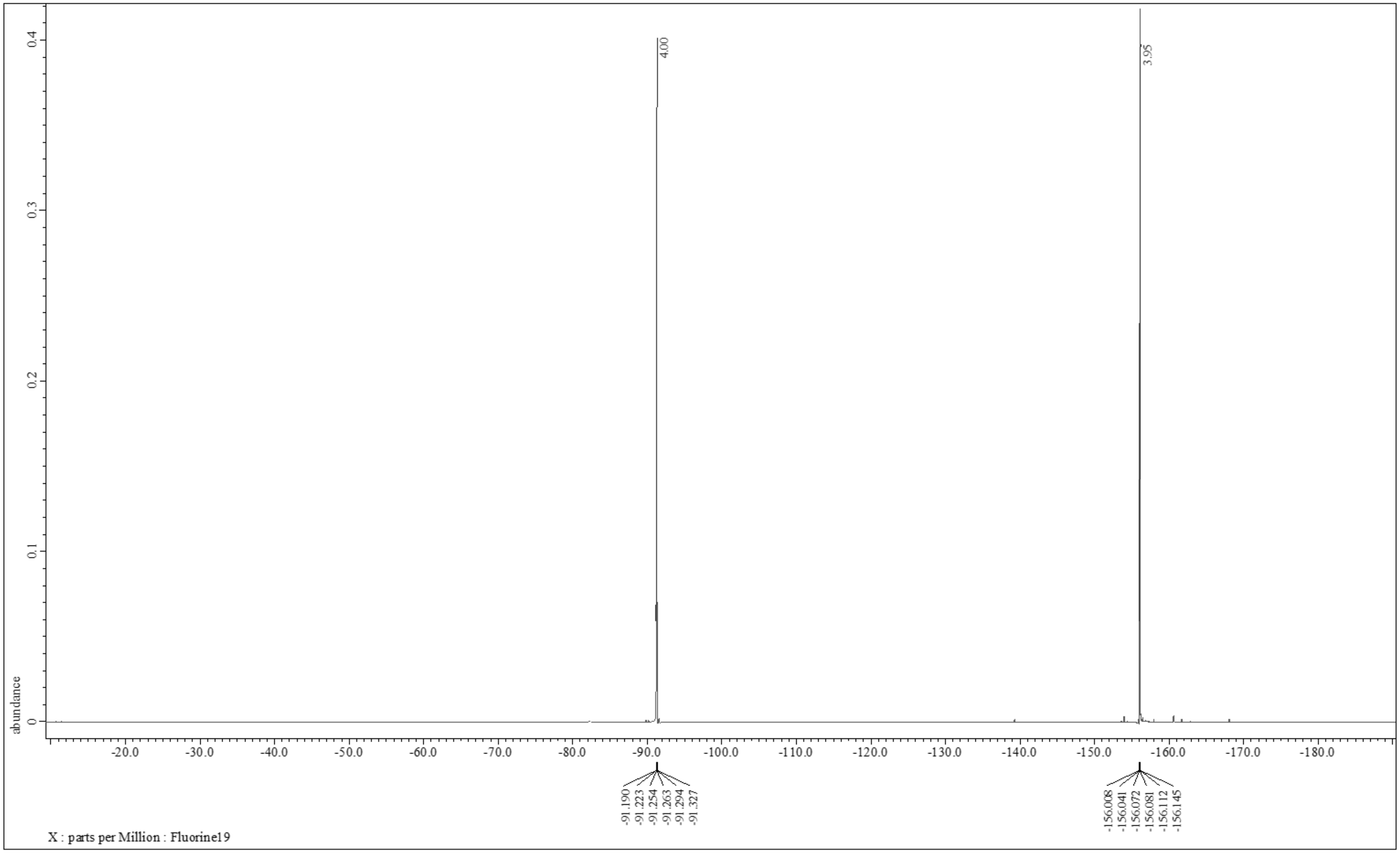

Figure S12. ^{19}F NMR of **7**.

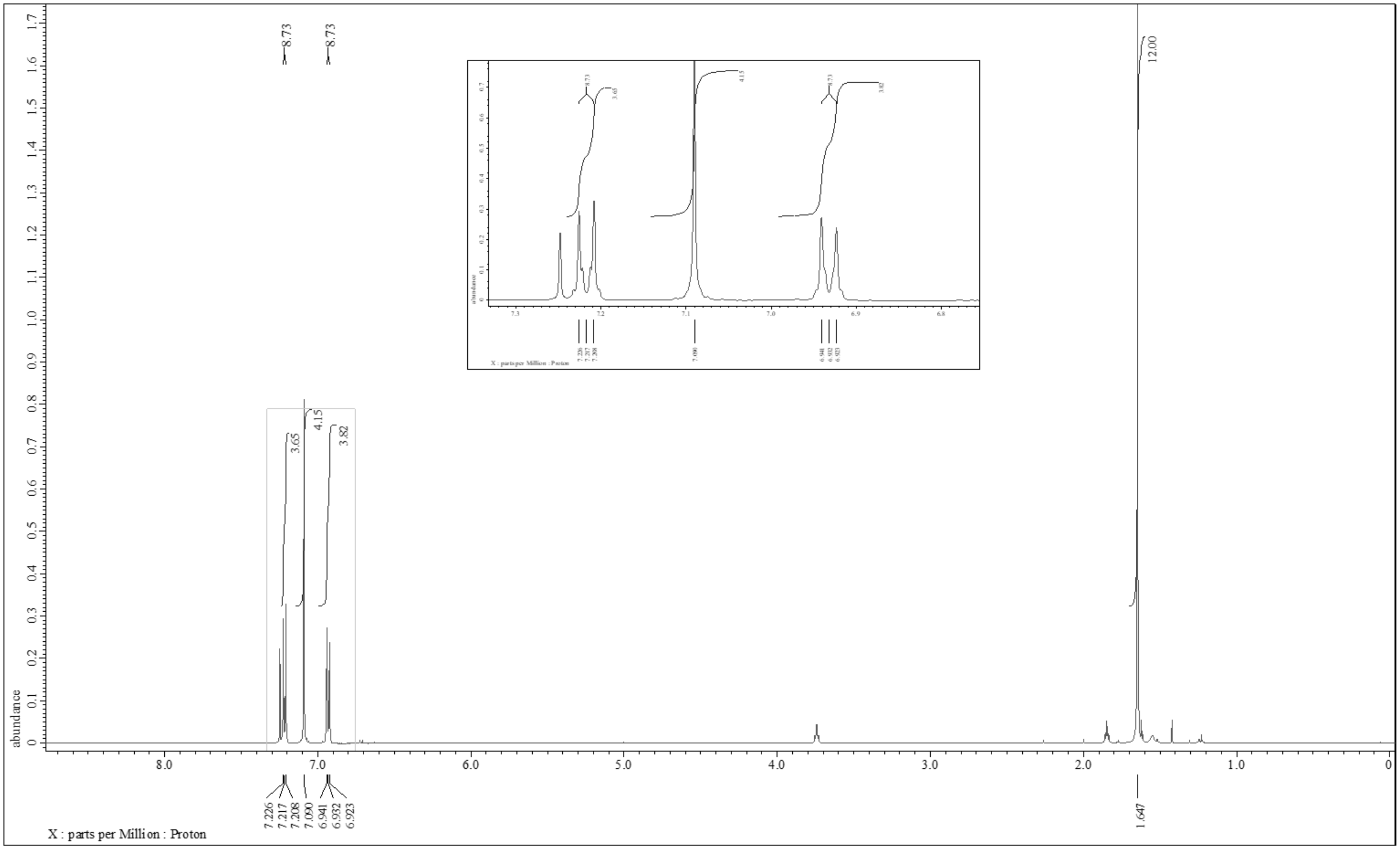

Figure S13. ^{1}H NMR of **8**.

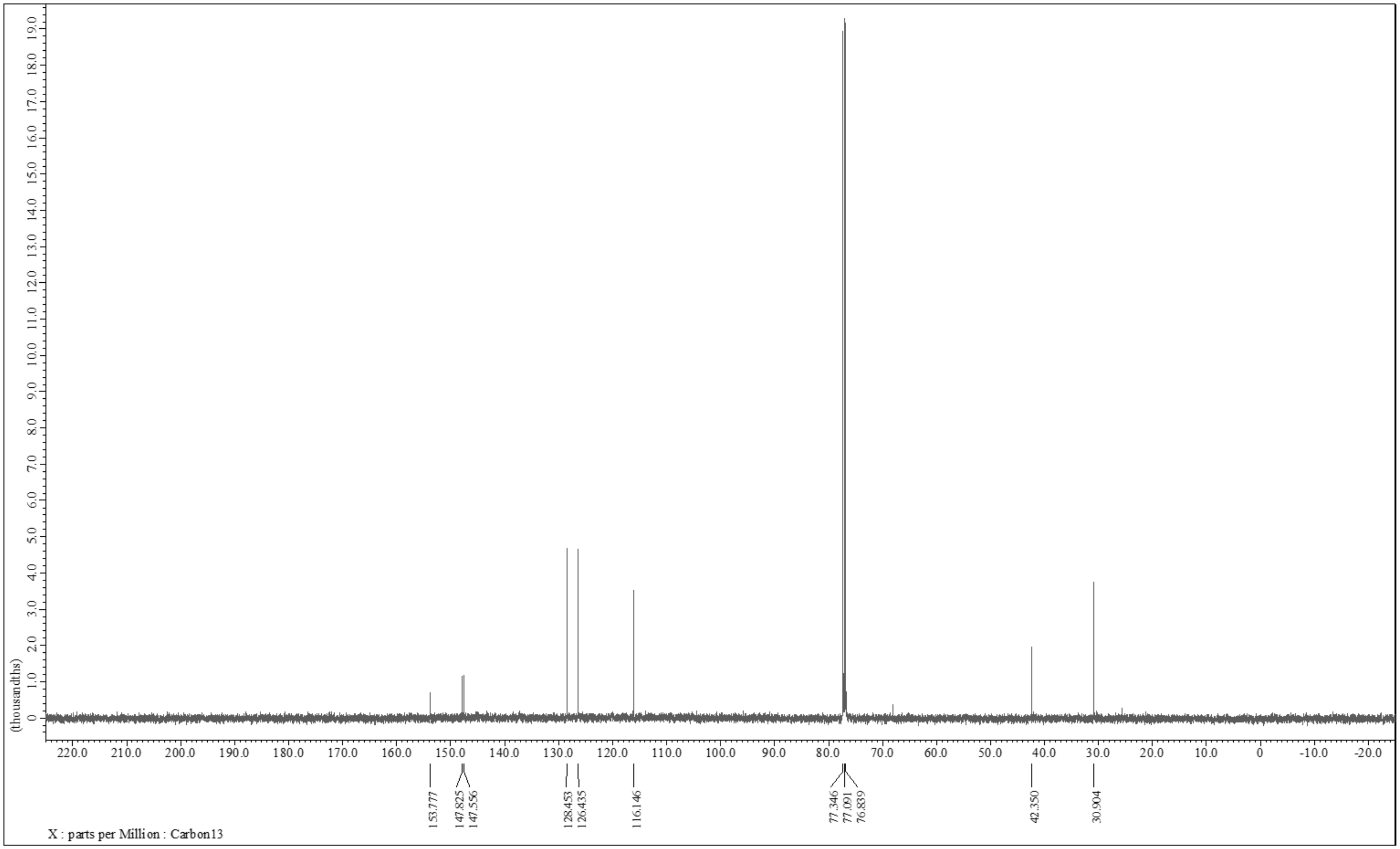

Figure S14. ^{13}C NMR of **8**.

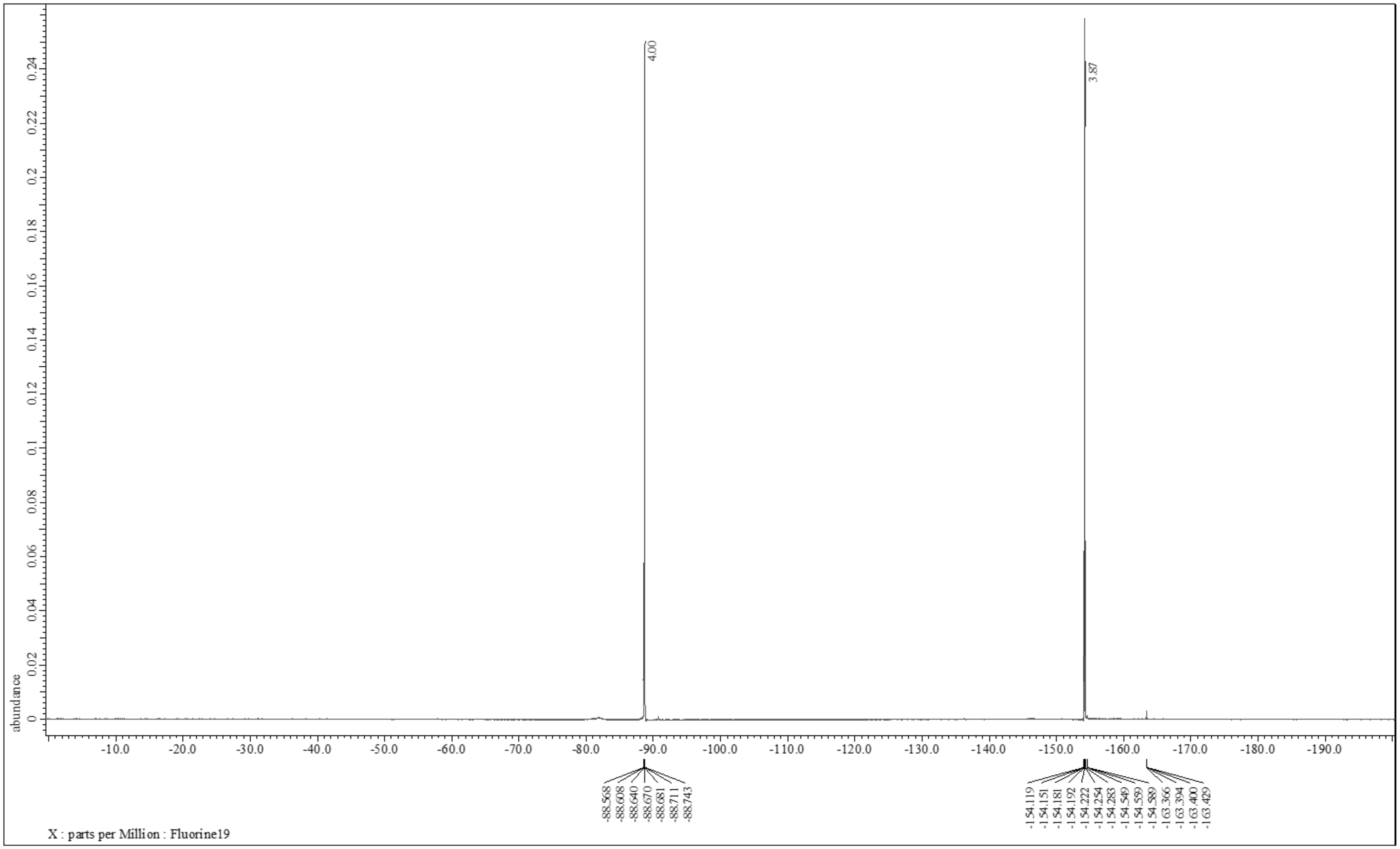

Figure S15. ^{19}F NMR of **8**.

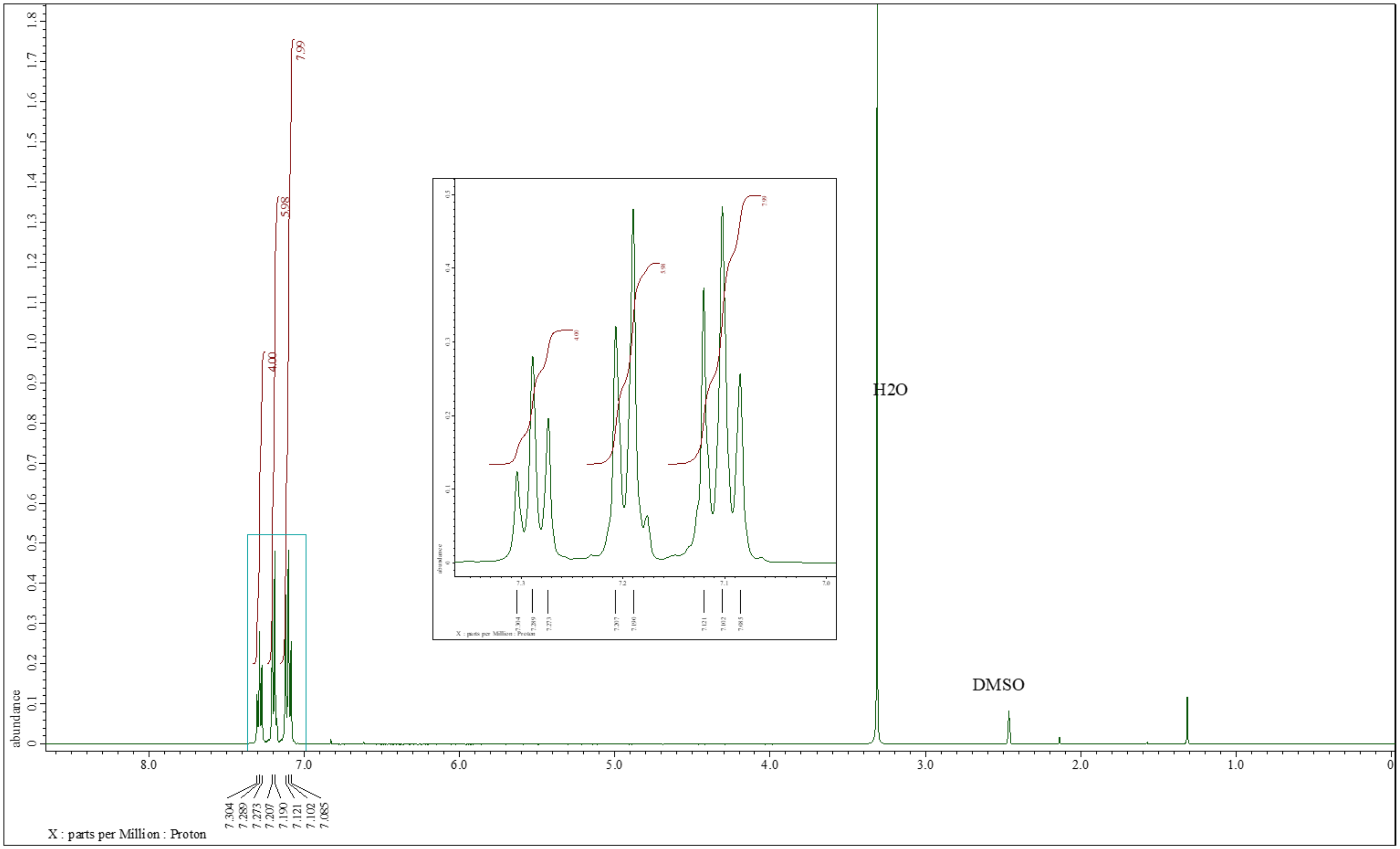

Figure S16. ^{1}H NMR of **9**.

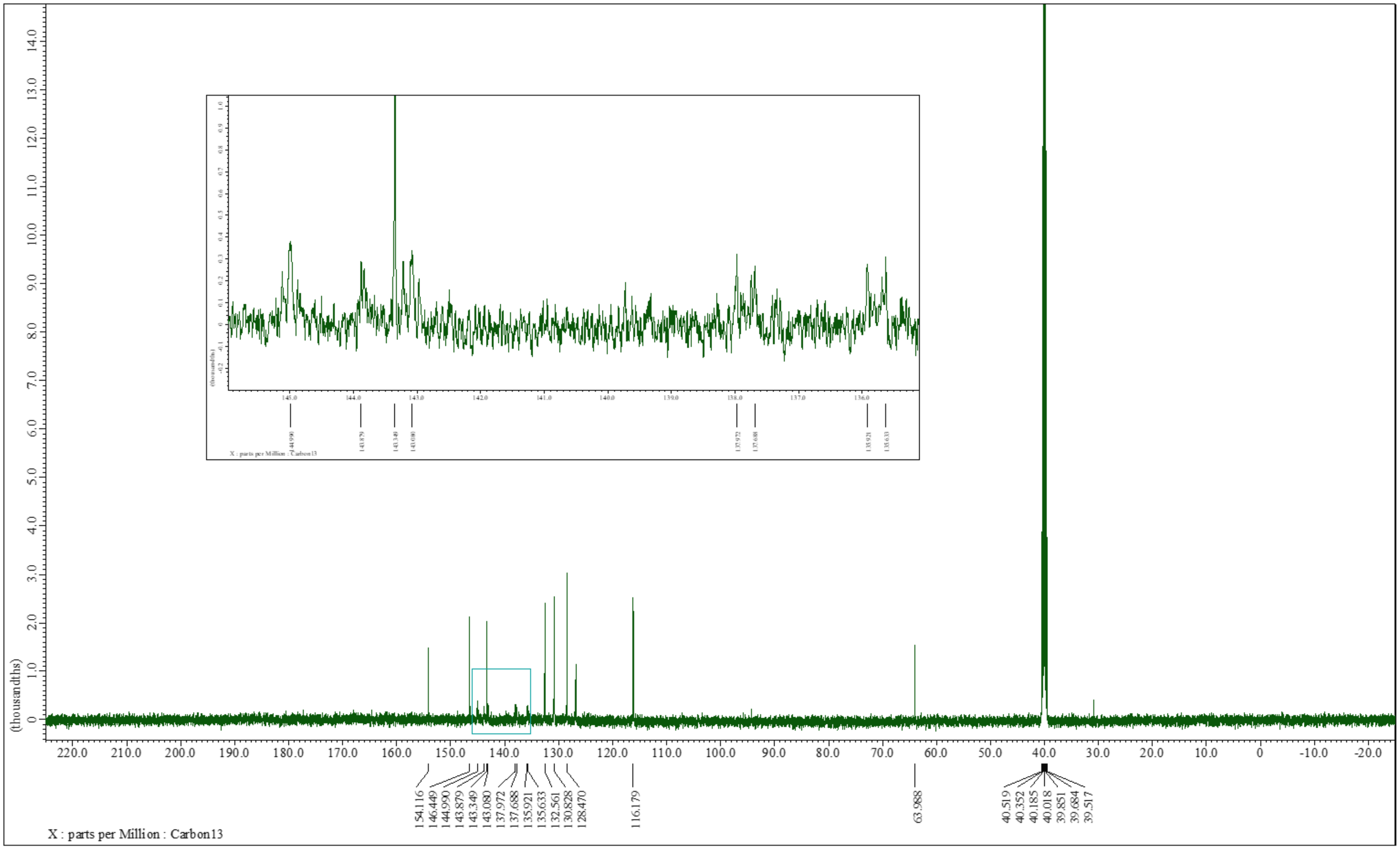

Figure S17. ^{13}C NMR of **9**.

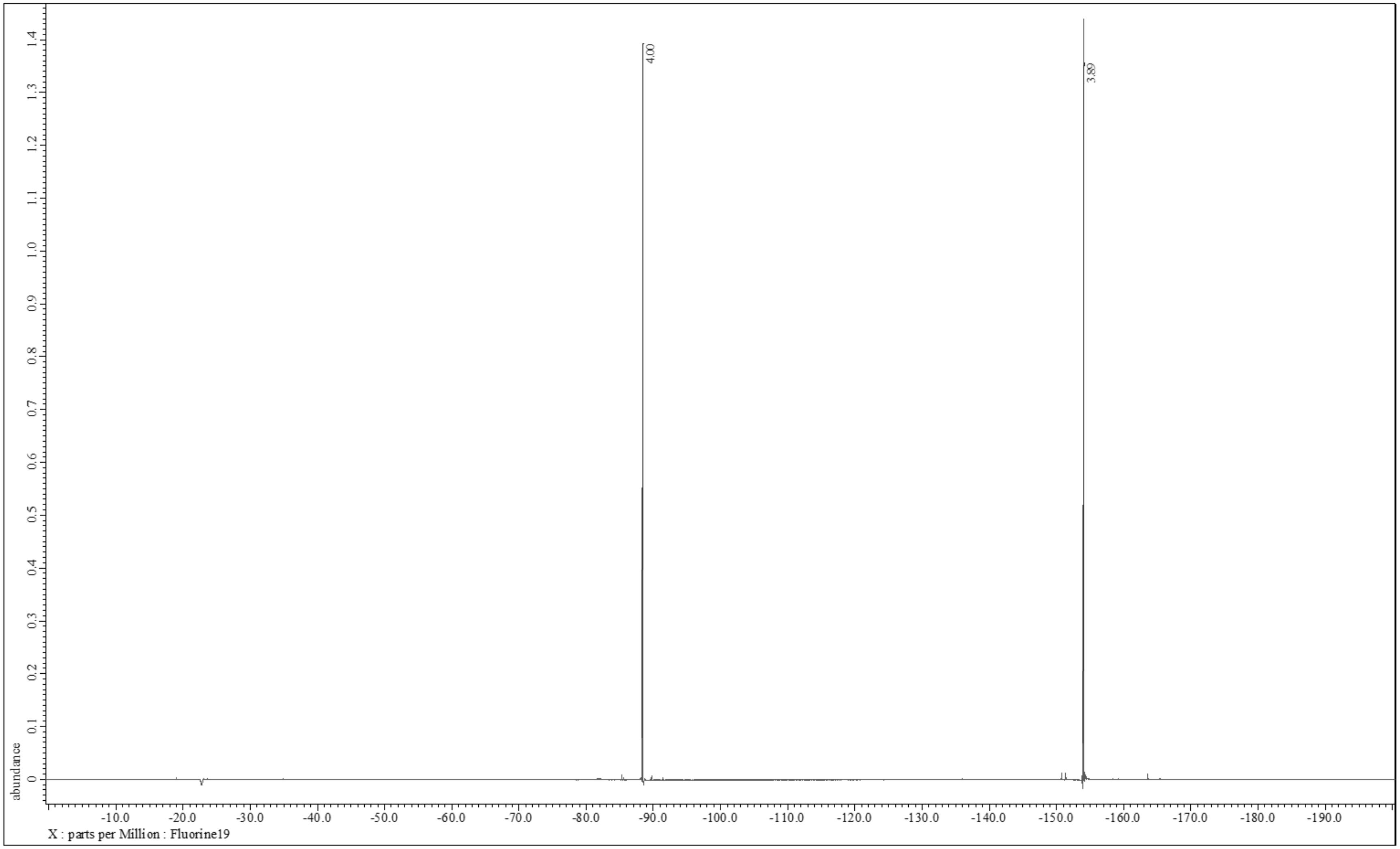

Figure S18. ^{19}F NMR of **9**.

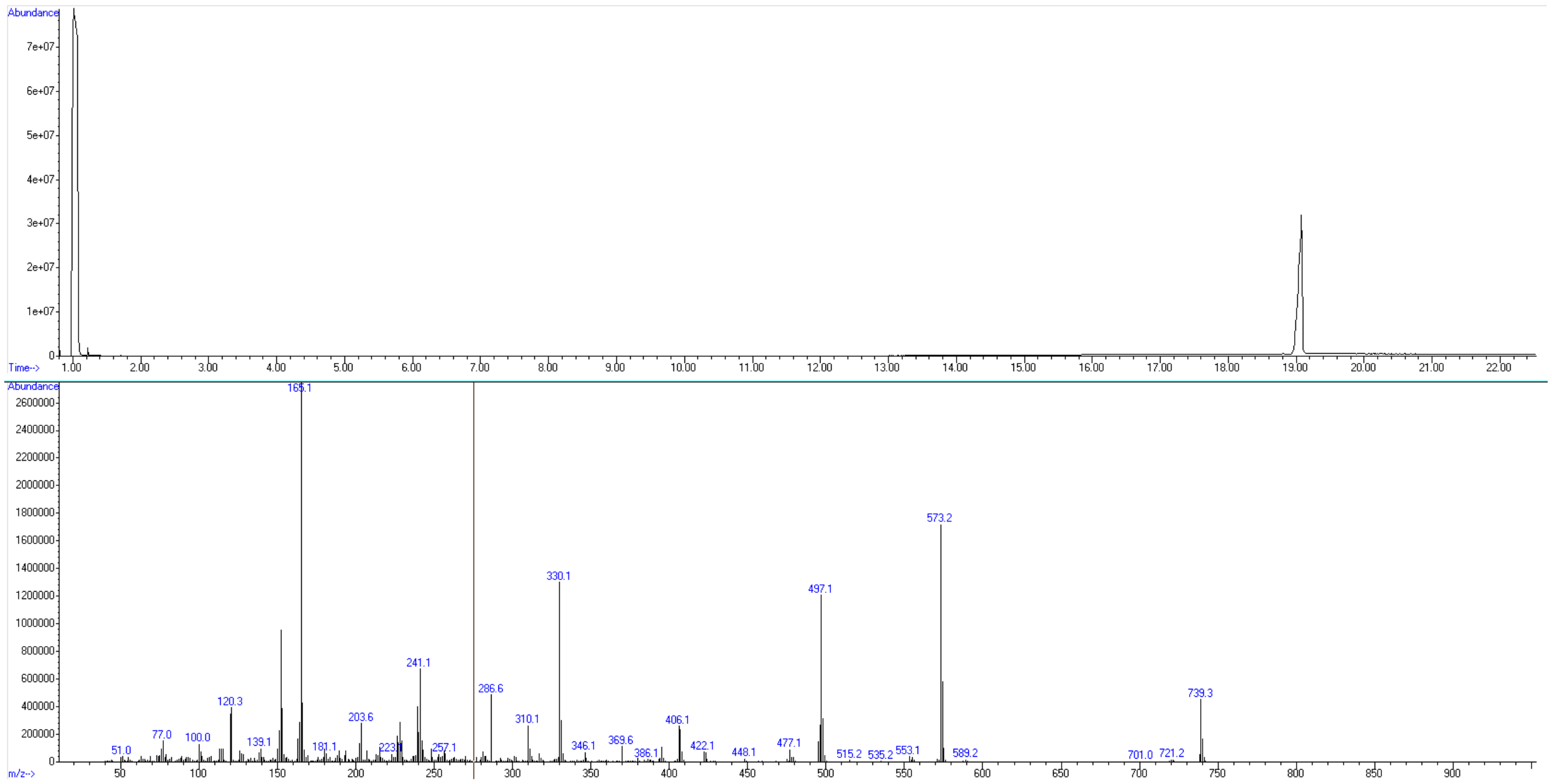

Figure S19. GCMS analysis of **3**.

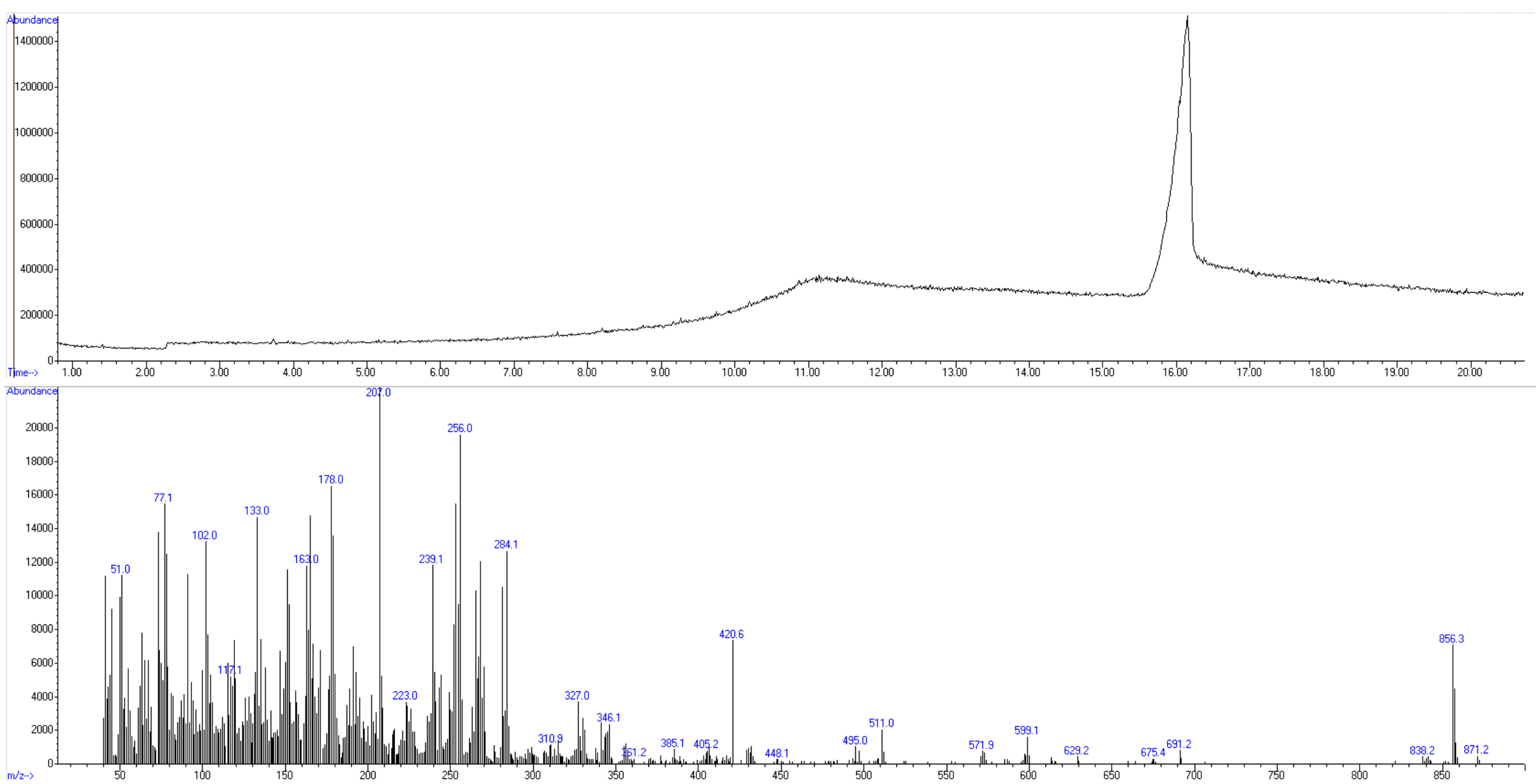

Figure S20. GCMS analysis of **5**.

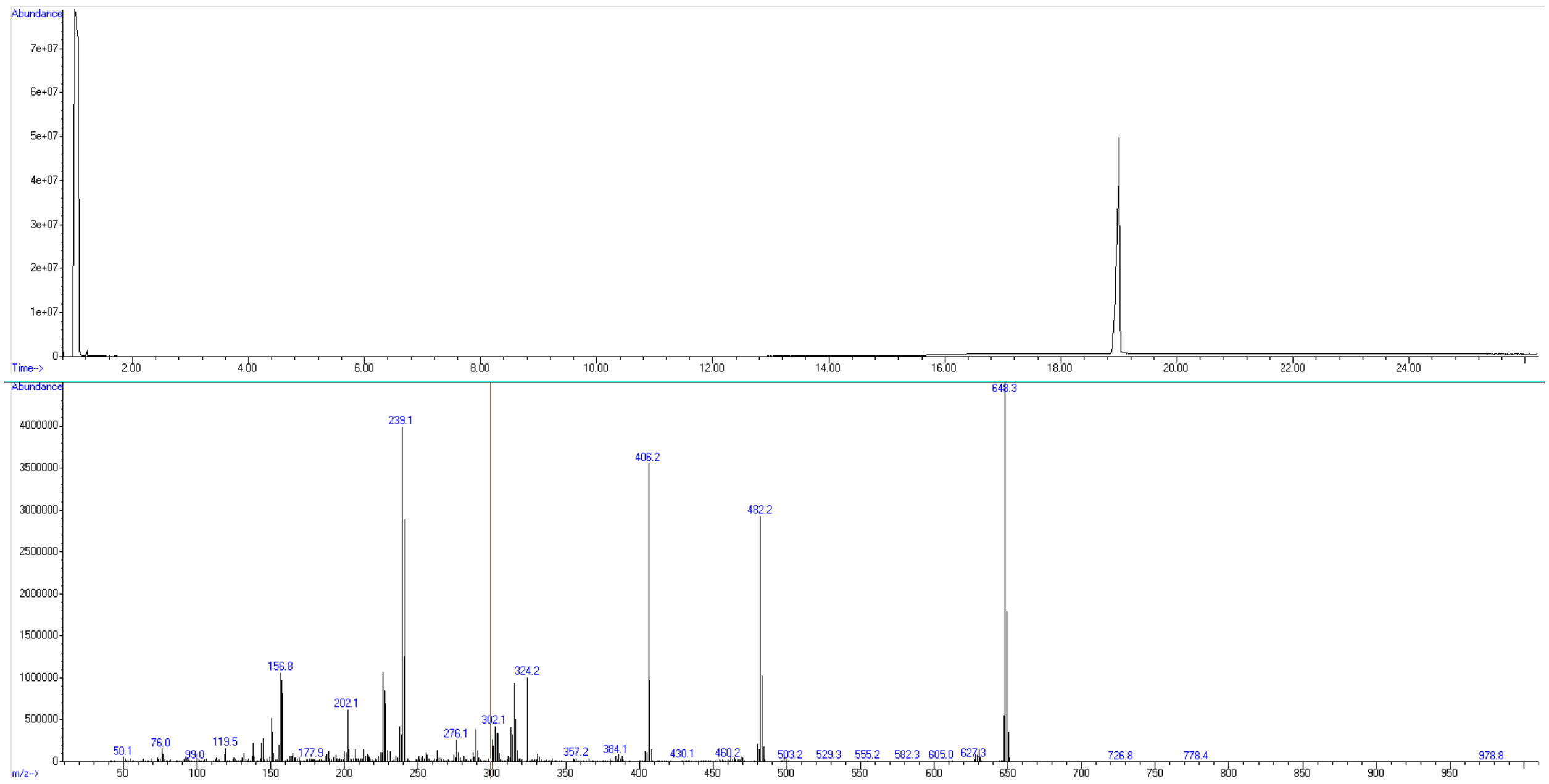

Figure S21. GCMS analysis of **6**.

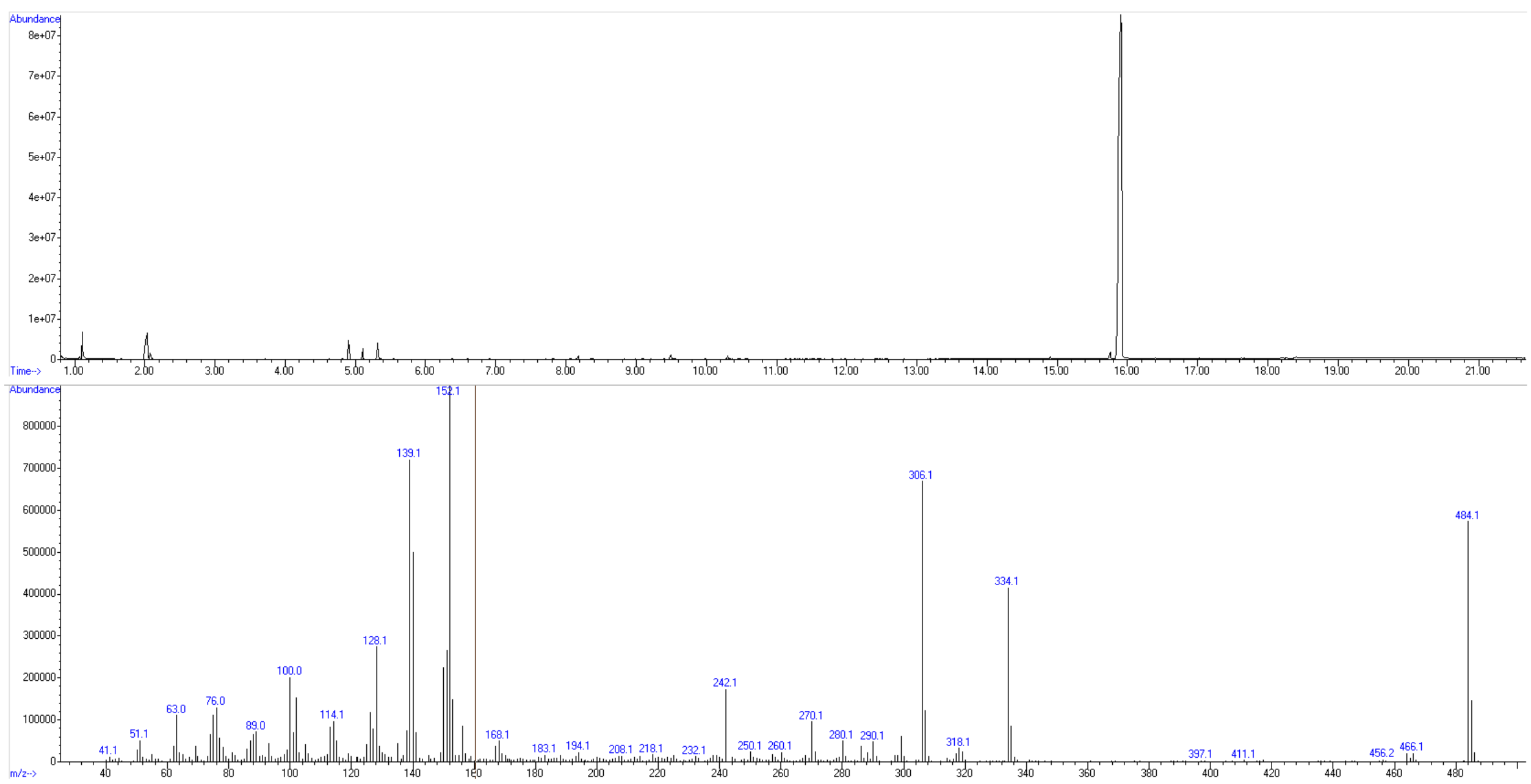

Figure S22. GCMS analysis of **7**.

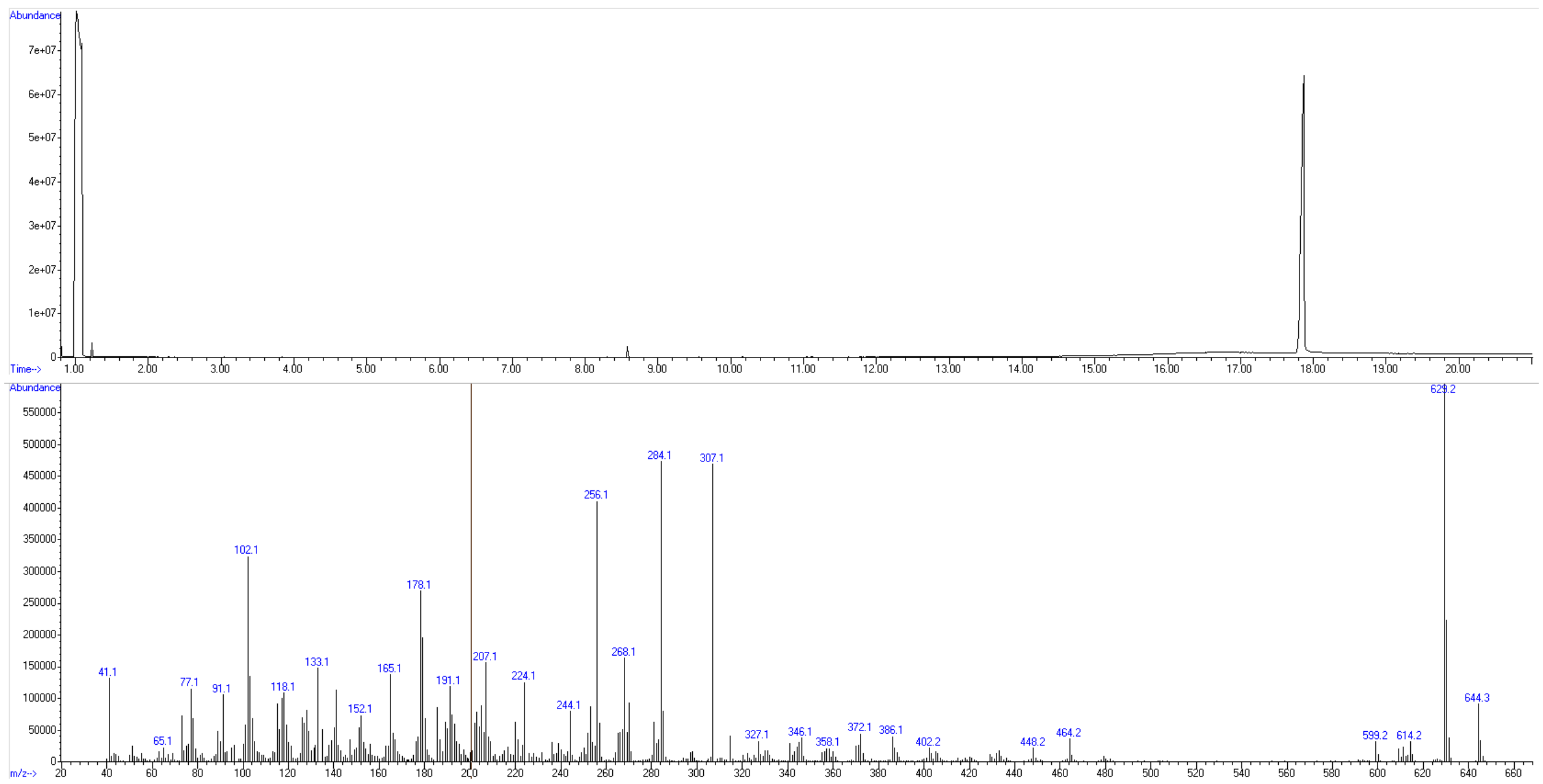

Figure S23. GCMS analysis of **8**.

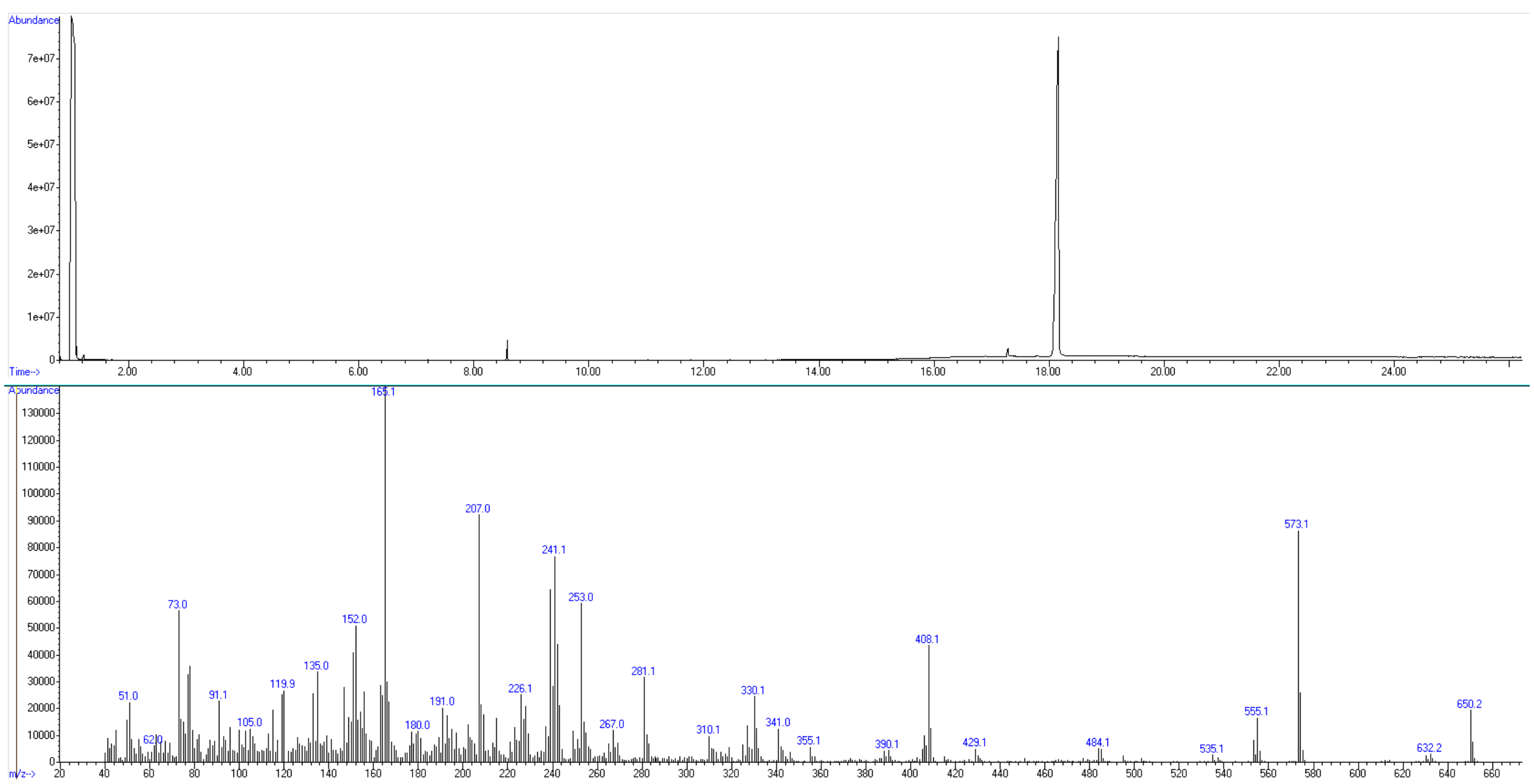

Figure S24. GCMS analysis of **9**.

Table S1. Glass Transition Temperatures of Melt Blends of **1**, **2**, and **4**–**6**

mol ratio	**1**:**2** T_g (°C)[a] [T_g (°C)][b]	**1**:**4** T_g (°C)[a] [T_g (°C)][b]	**1**:**5** T_g (°C)[a] [T_g (°C)][b]	**2**:**5** T_g (°C)[a] [T_g (°C)][b]	**1**:**6** T_g (°C)[a] [T_g (°C)][b]
1:0	−15 [—]	−15 [—]	−15 [—]	0 [—]	−15 [—]
3:1	−11 [−11]	−6 [−6]	−3 [−1]	8 [6]	0 [−1]
1:1	−7 [−6]	4 [3]	9 [13]	16 [13]	14 [12]
1:3	−4 [−3]	13 [13]	23 [23]	25 [26]	32 [39]
0:1	0 [—]	21 [—]	32 [—]	32 [—]	53 [—]

[a]DSC (fourth cycle, heat 10 °C/min). [b]Calculated from Flory-Fox equation using weight fraction: $1/T_g = w_1/T_{g1} + w_2/T_{g2}$.

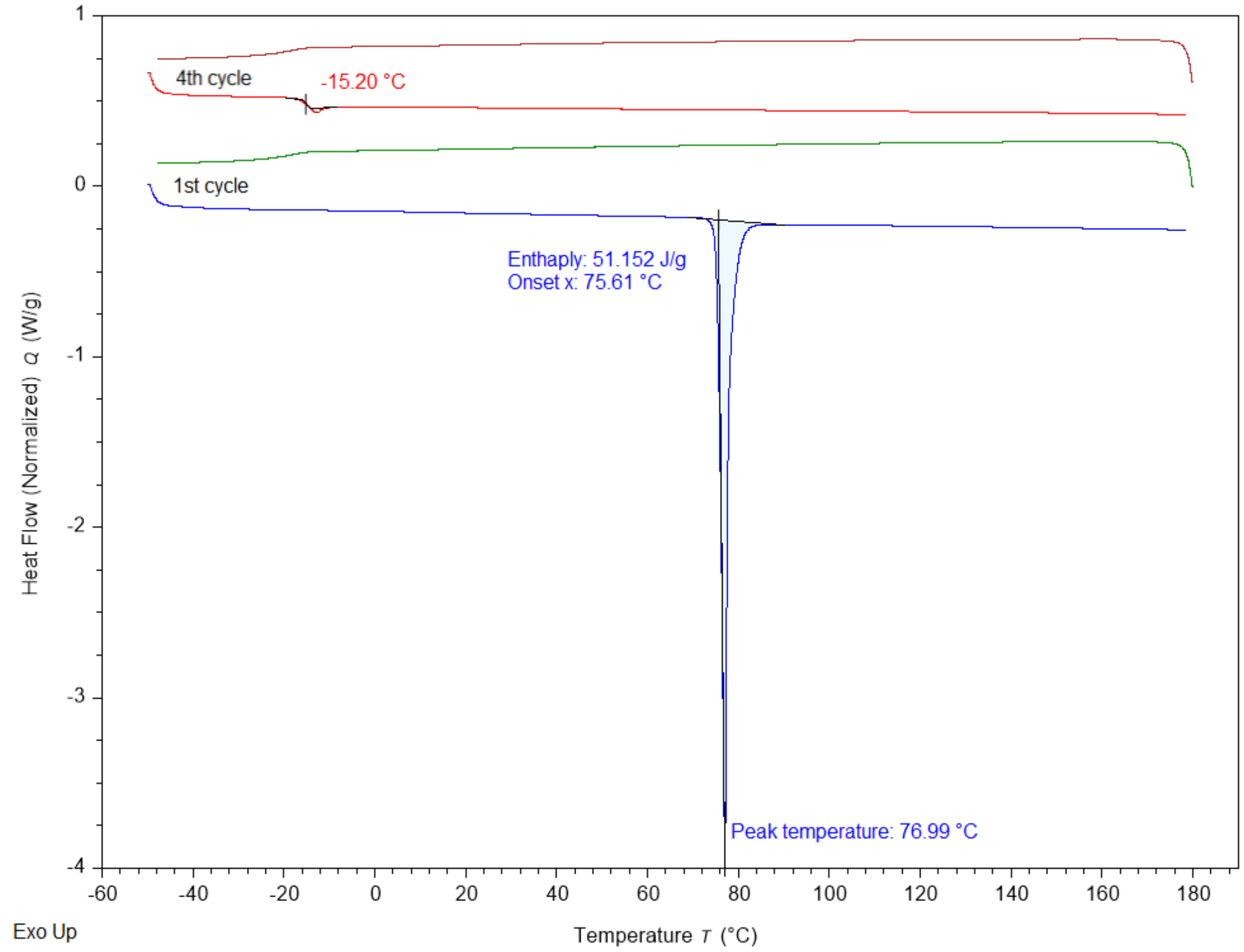

Figure S25. DSC analysis of **1**.

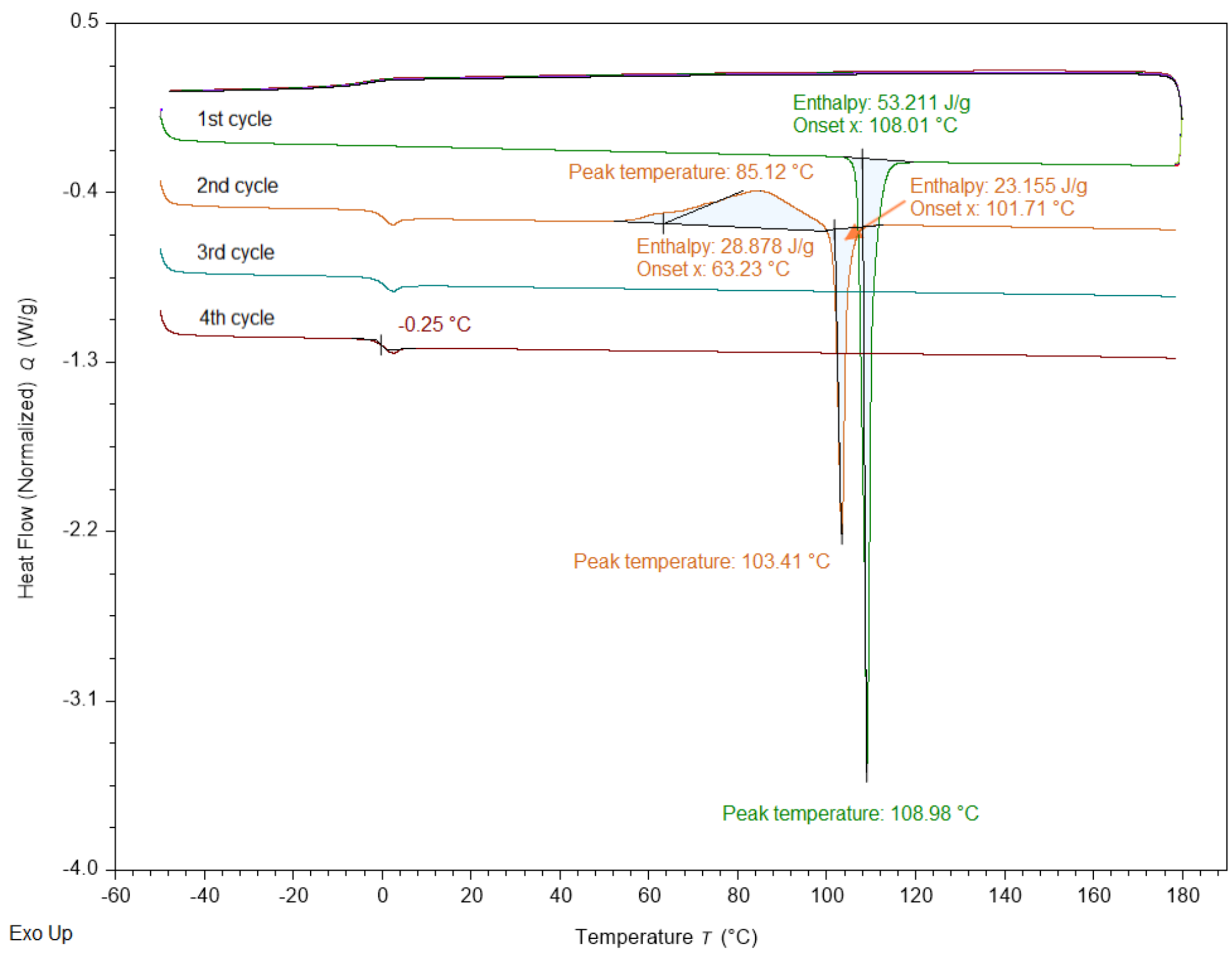

Figure S26. DSC analysis of **2**.

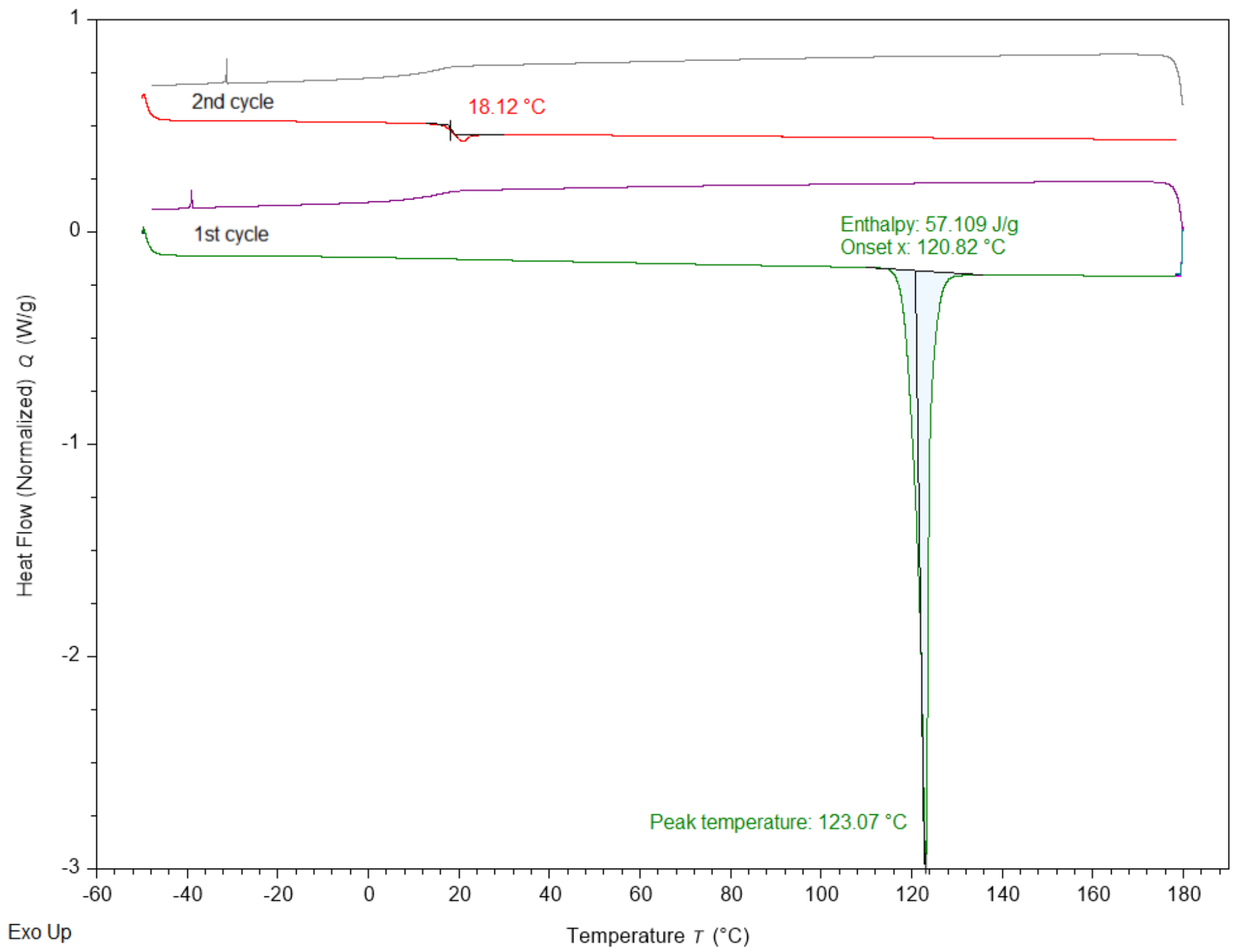

Figure S27. DSC analysis of **3**.

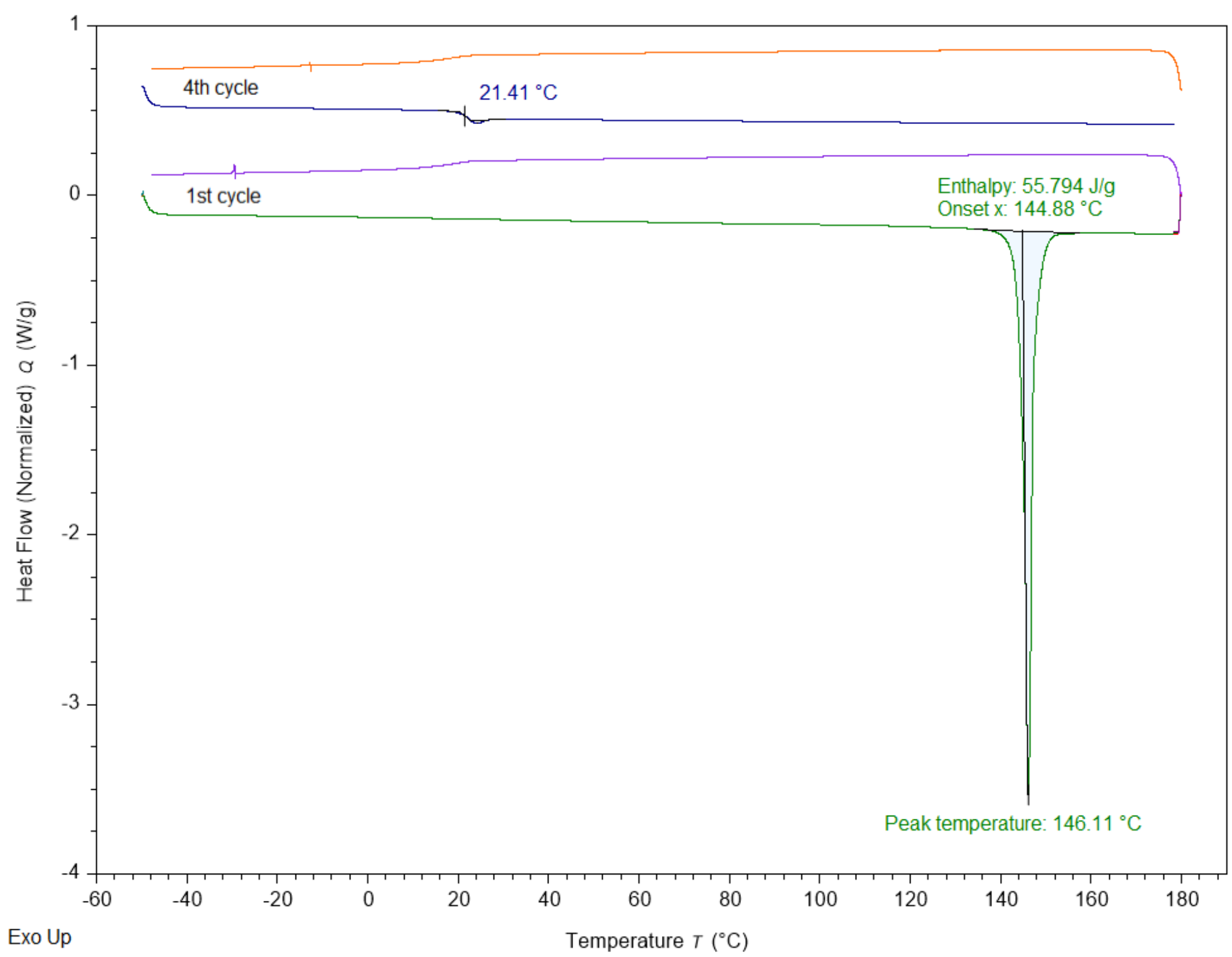

Figure S28. DSC analysis of **4**.

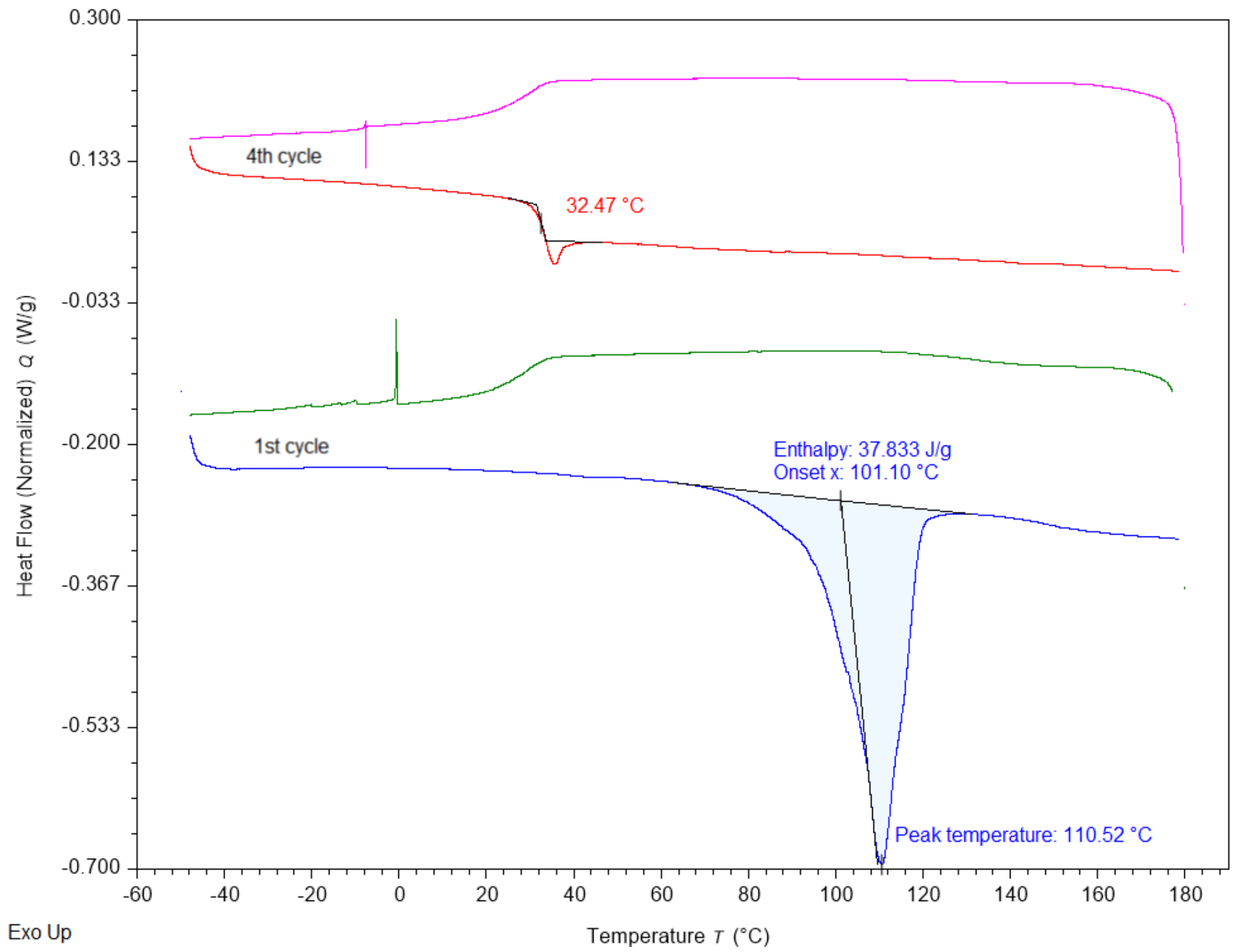

Figure S29. DSC analysis of **5**.

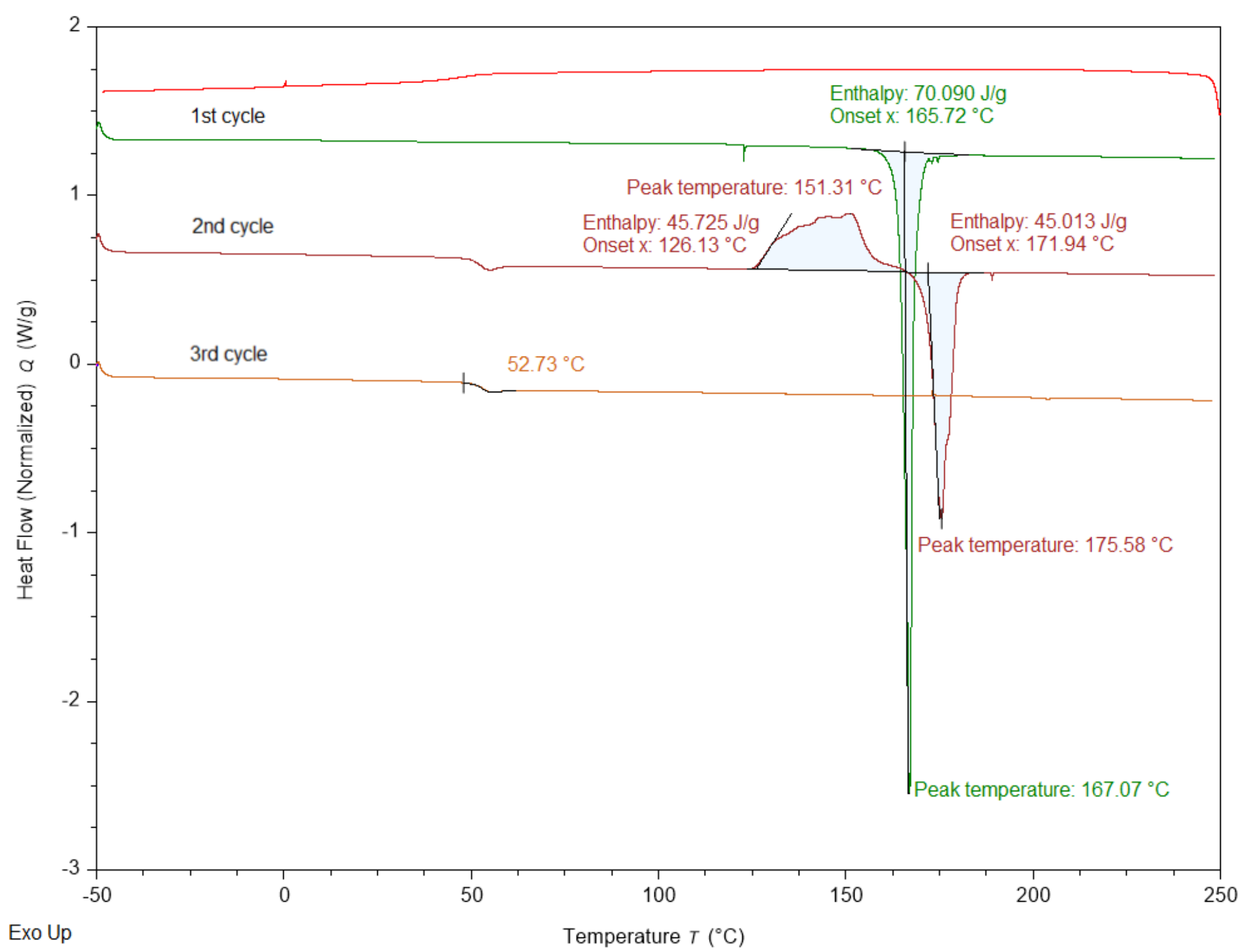

Figure S30. DSC analysis of **6**.

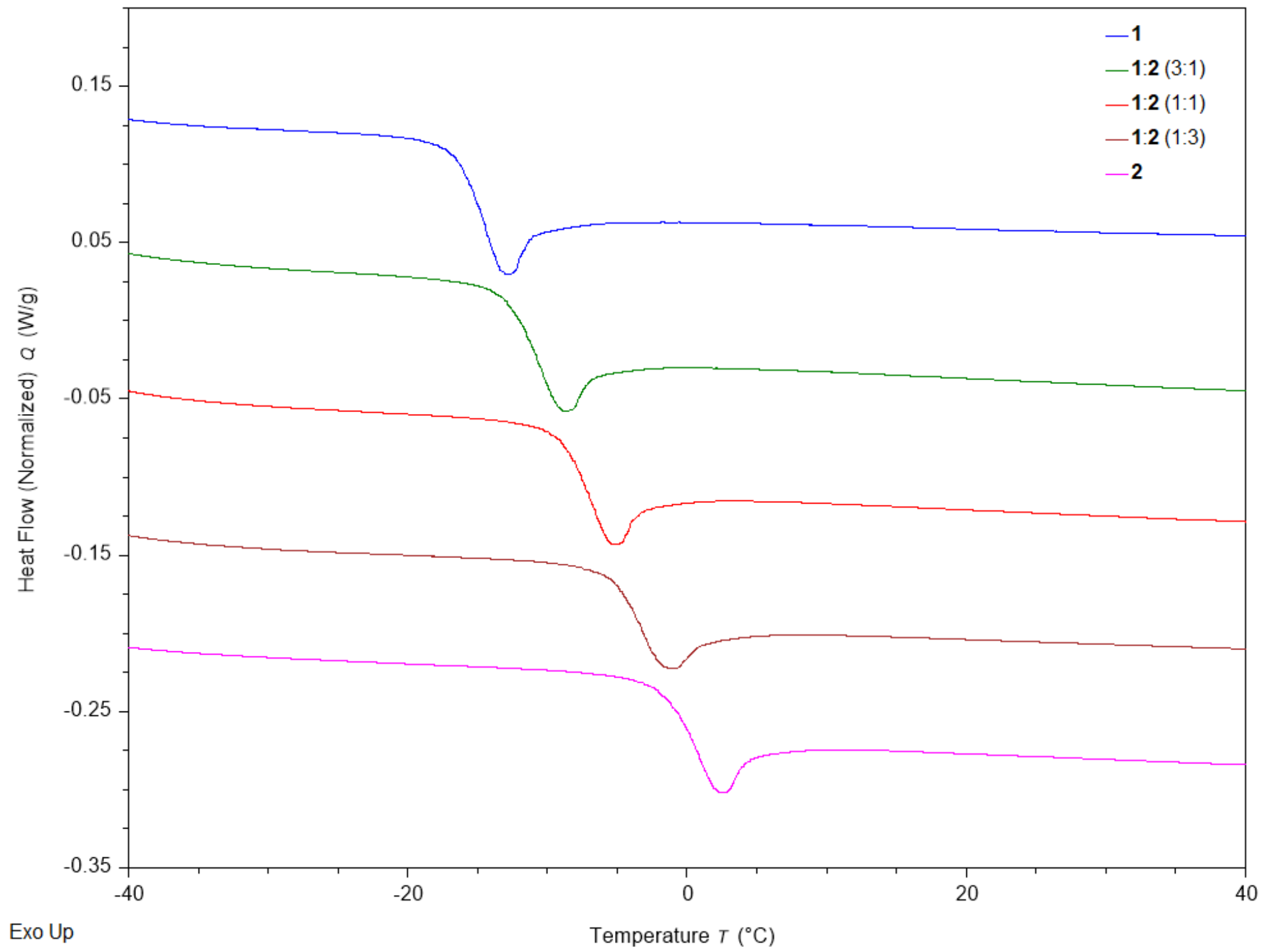

Figure S31. DSC analysis of **1** and **2** formulations.

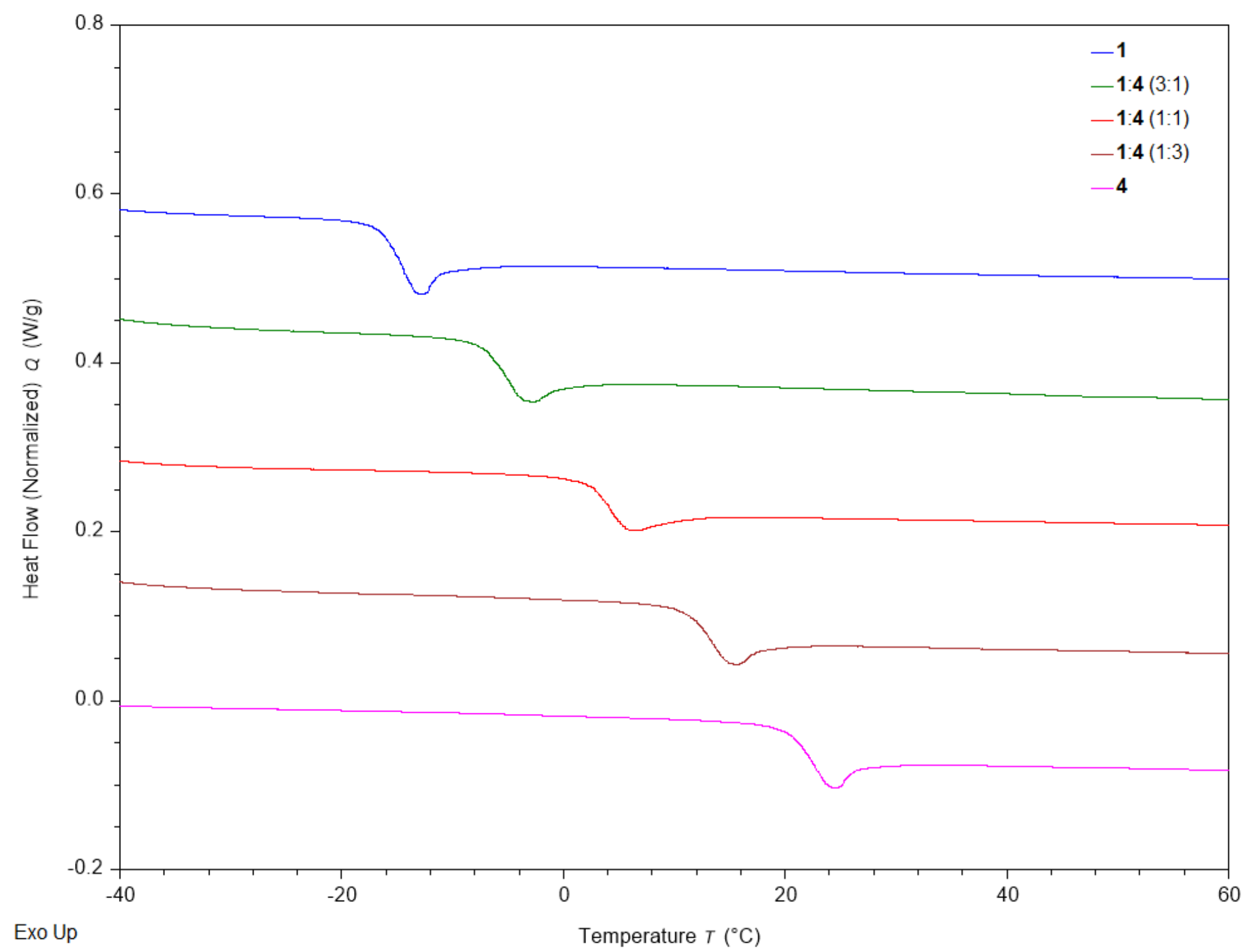

Figure S32. DSC analysis of **1** and **4** formulations.

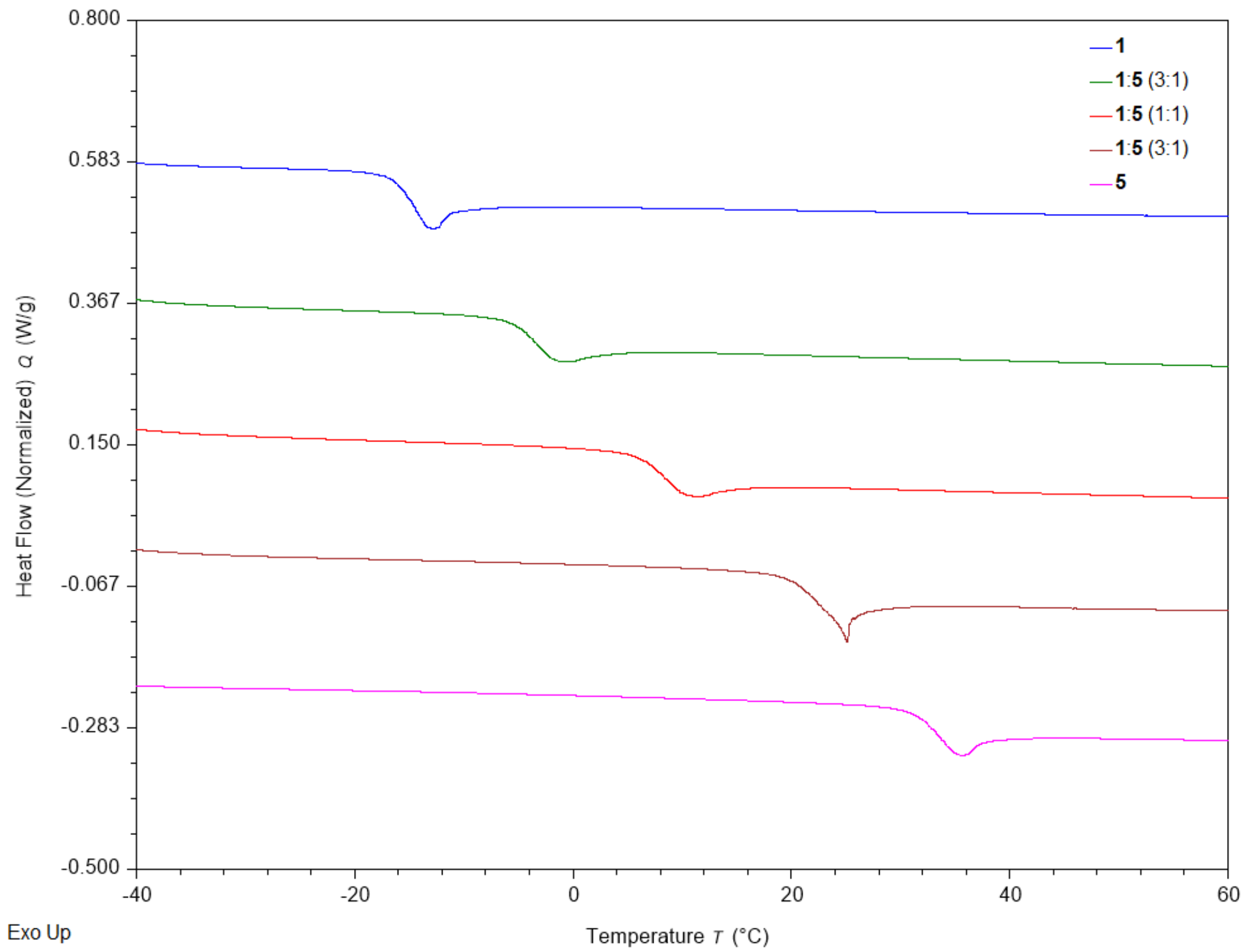

Figure S33. DSC analysis of **1** and **5** formulations.

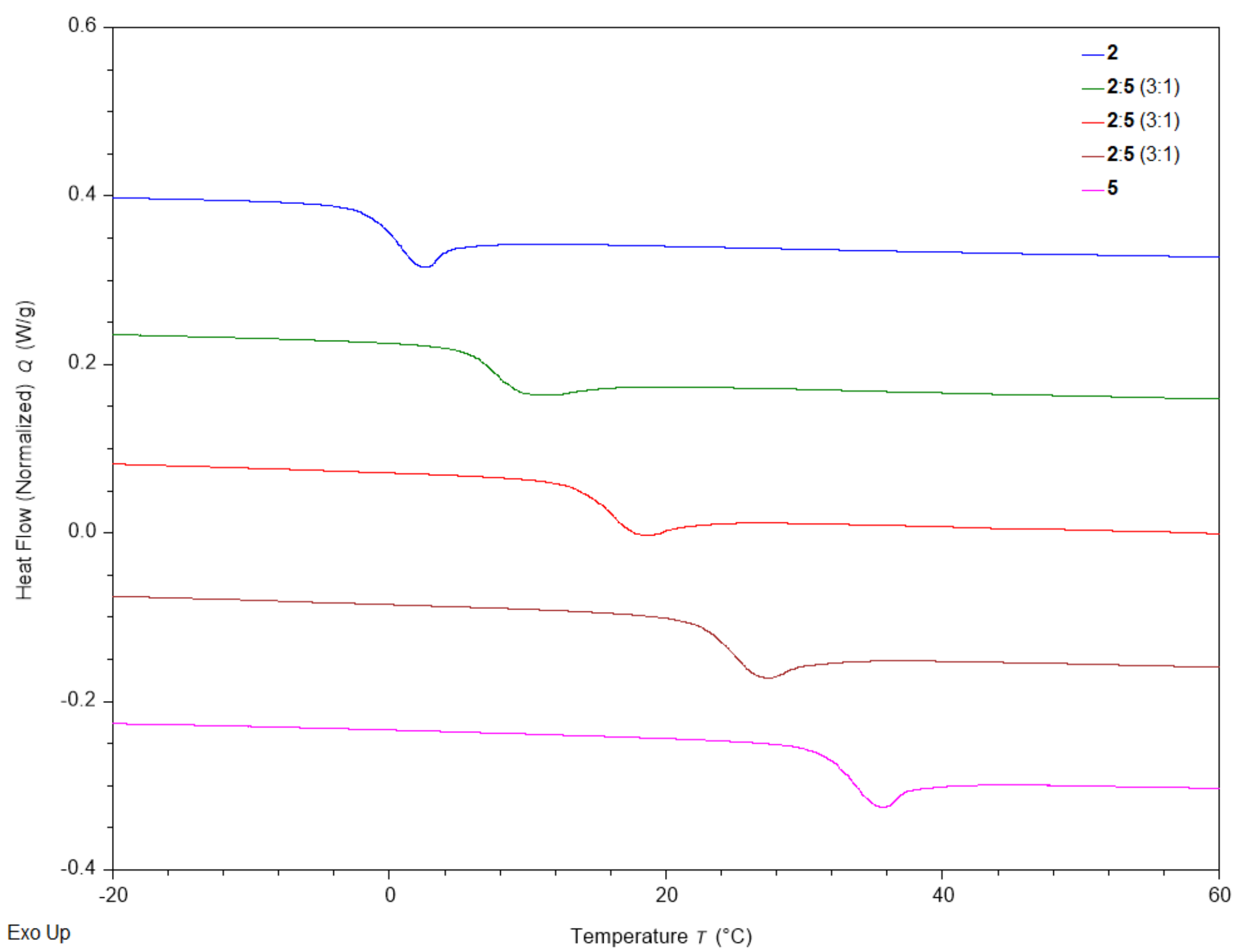

Figure S34. DSC analysis of **2** and **5** formulations.

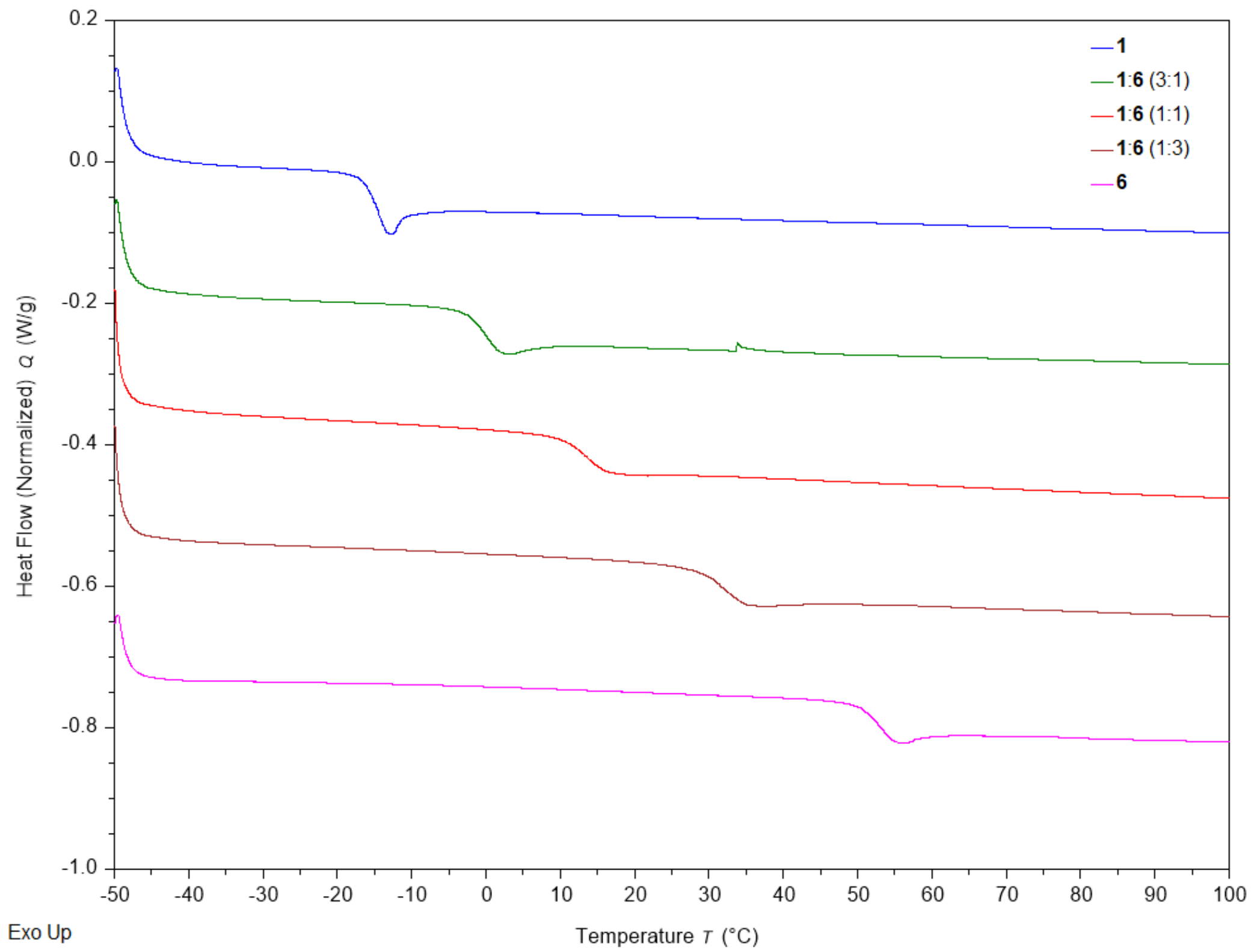

Figure S35. DSC analysis of **1** and **6** formulations.

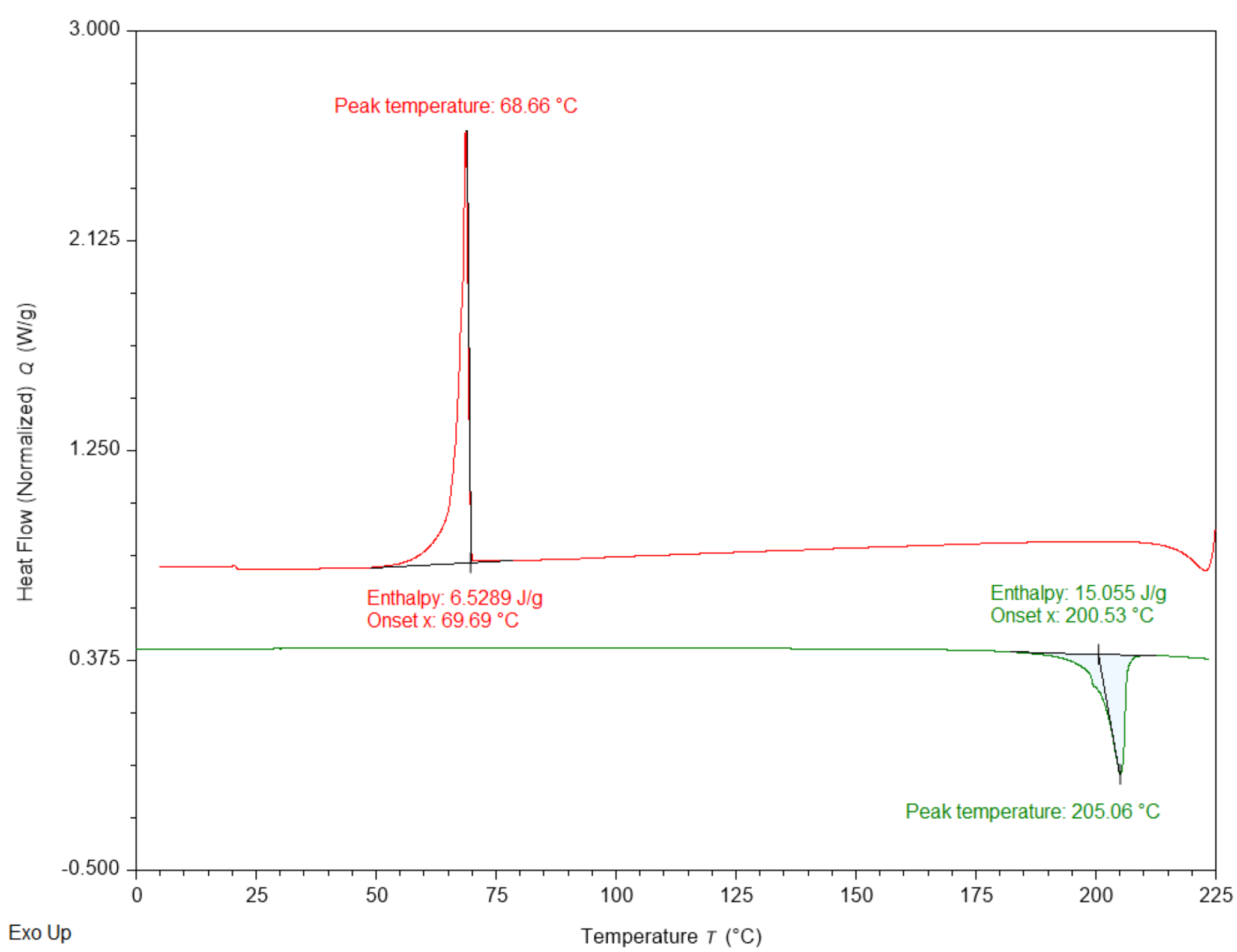

Figure S36. DSC analysis of **7** second heating cycle.

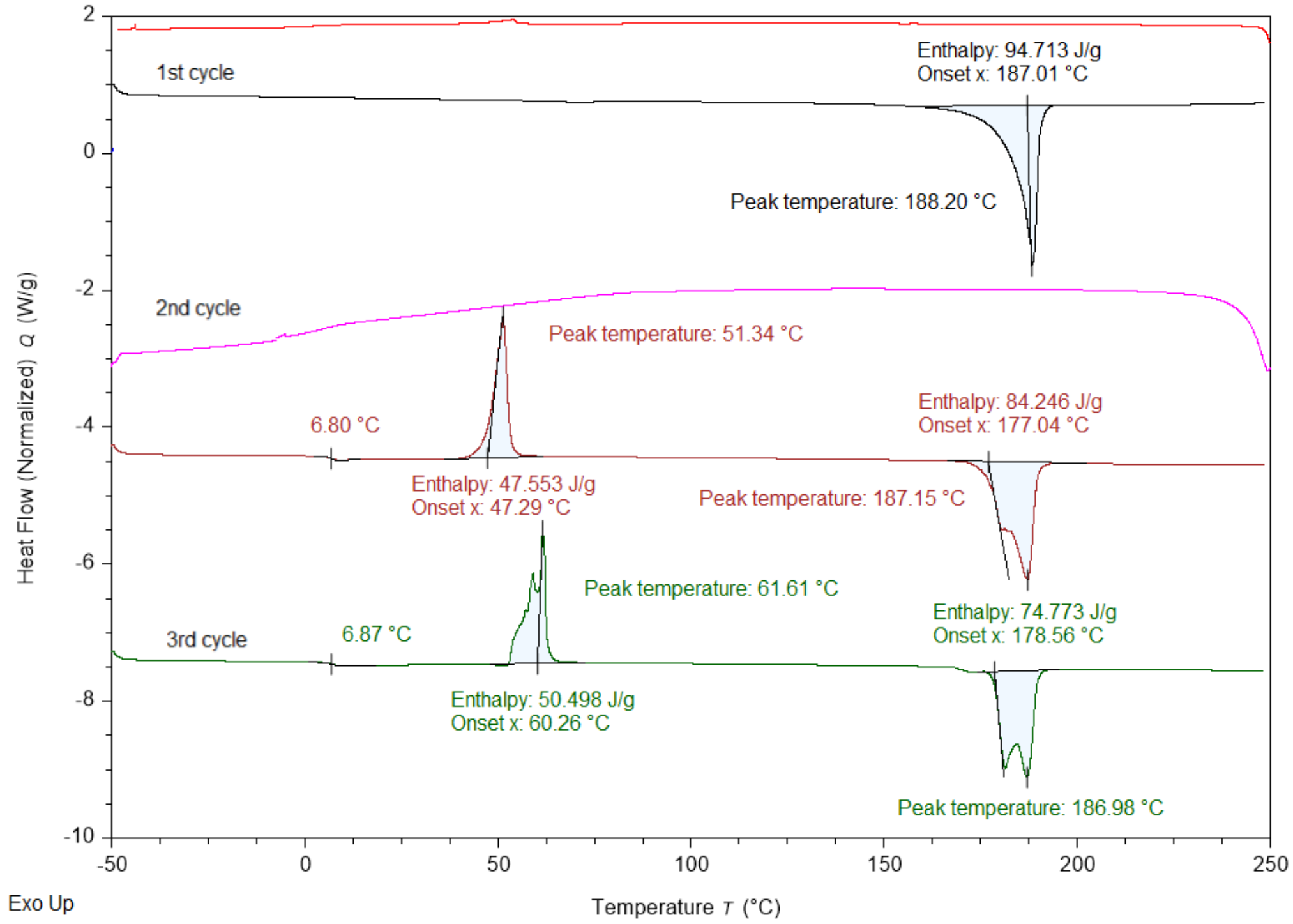

Figure S37. DSC analysis of **8**.

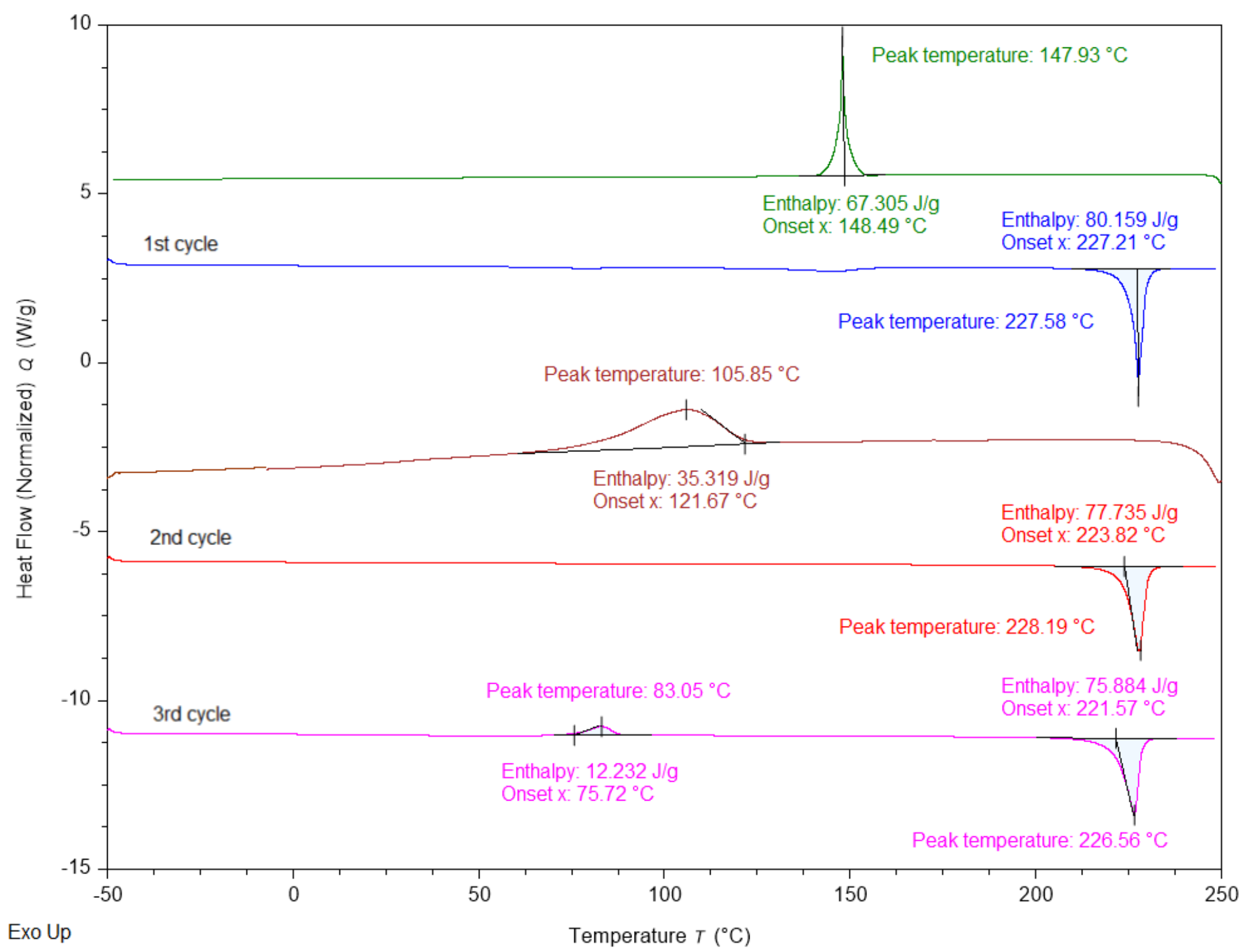

Figure S38. DSC analysis of **9**.

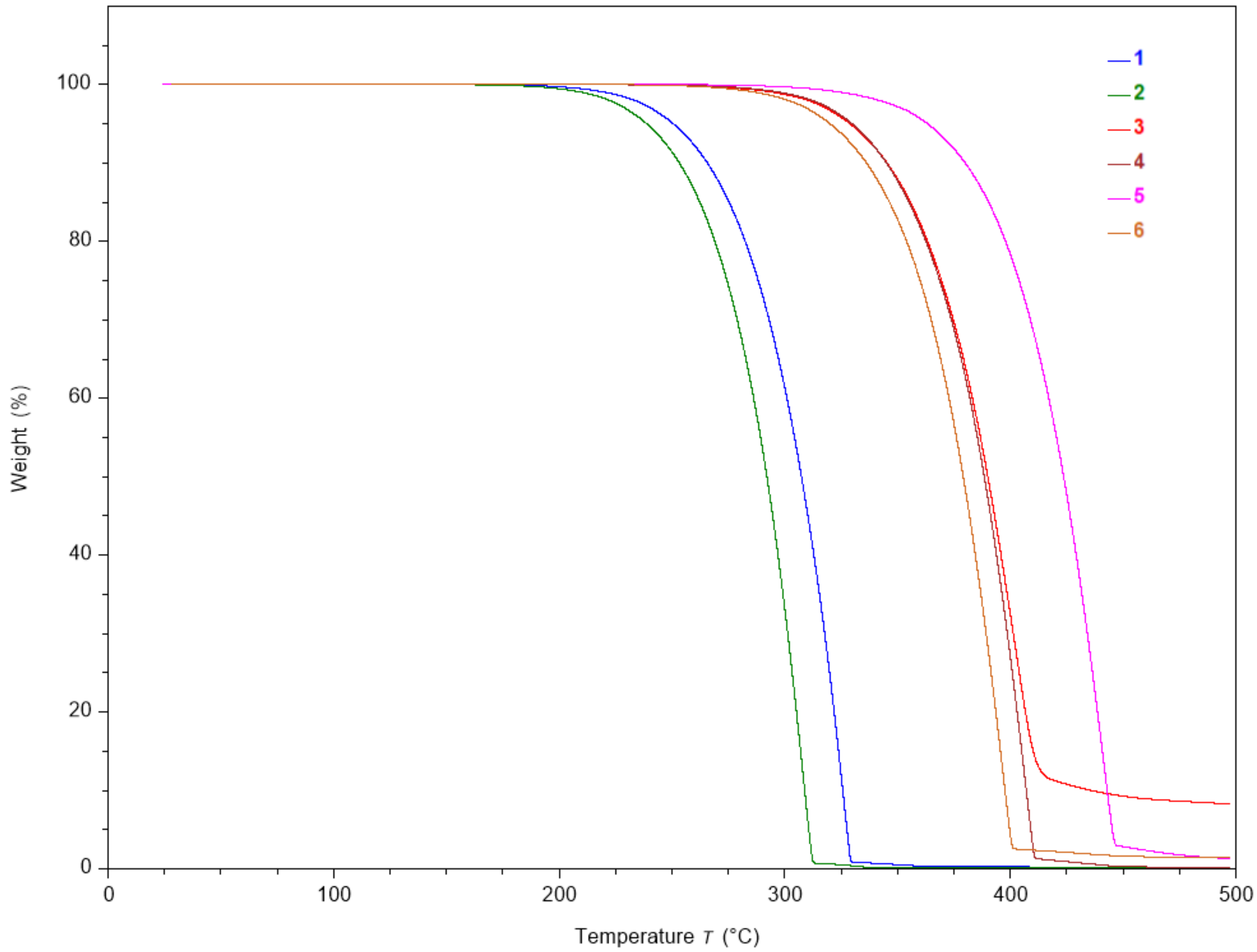

Figure S39. TGA analysis of **1**−**6**.

Table S2. Glass Transition Temperature Deviations ($\Delta T_{calc-exp}$) of Melt Blends of **1**, **2**, and **4**−**6**.

mol ratio	**1:2**	**1:4**	**1:5**	**2:5**	**1:6**
1:0	0	0	0	0	0
3:1	0	0	2	-2	-1
1:1	1	-1	4	-3	-2
1:3	1	0	0	1	7
0:1	0	0	0	0	0

3 (CCDC #: 2483251)

Computing details

Data collection: *CrysAlis PRO* system (CCD 43.105a 64-bit (release 11-01-2024)); cell refinement: *CrysAlis PRO* 1.171.43.90 (Rigaku OD, 2023); data reduction: *CrysAlis PRO* 1.171.43.90 (Rigaku OD, 2023); program(s) used to solve structure: SHELXT 2018/2 (Sheldrick, 2018); program(s) used to refine structure: *SHELXL* 2019/3 (Sheldrick, 2015); molecular graphics: Olex2 1.5 (Dolomanov *et al.*, 2009); software used to prepare material for publication: Olex2 1.5 (Dolomanov *et al.*, 2009).

Crystal data

$C_{34}H_{13}F_{12}N_3O_3$
M_r = 739.47
Monoclinic, $P2_1/n$
a = 15.3643 (2) Å
b = 7.7371 (1) Å
c = 24.4621 (3) Å
β = 93.537 (1)°
V = 2902.40 (6) Å^3
Z = 4
F(000) = 1480
D_x = 1.692 Mg m^{-3}
Melting point: 396 K
Cu $K\alpha$ radiation, λ = 1.54184 Å
Cell parameters from 20786 reflections
θ = 3.3–68.6°
μ = 1.44 mm^{-1}
T = 106 K
Rect. Prism, clear colourless
0.45 × 0.13 × 0.04 mm

Data collection

XtaLAB Synergy, Dualflex, HyPix3000 diffractometer
Radiation source: micro-focus sealed X-ray tube, PhotonJet (Cu) X-ray Source
Mirror monochromator
Detector resolution: 10.0000 pixels mm^{-1}
ω scans
Absorption correction: multi-scan *CrysAlis PRO* 1.171.43.90 (Rigaku Oxford Diffraction, 2023) Numerical absorption correction based on gaussian integration over a multifaceted crystal model Empirical absorption correction using spherical harmonics, implemented in SCALE3 ABSPACK scaling algorithm.
T_{min} = 0.449, T_{max} = 1.000
29621 measured reflections
5349 independent reflections
4781 reflections with $I > 2\sigma(I)$
R_{int} = 0.032
θ_{max} = 68.8°, θ_{min} = 3.3°
h = −17→18
k = −9→8
l = −29→29

Refinement

Refinement on F^2
Least-squares matrix: full
$R[F^2 > 2\sigma(F^2)]$ = 0.033
$wR(F^2)$ = 0.091
S = 1.06
5349 reflections
469 parameters
6 restraints
Primary atom site location: dual
Hydrogen site location: inferred from neighbouring sites
H-atom parameters constrained
$w = 1/[\sigma^2(F_o^2) + (0.043P)^2 + 1.2027P]$ where $P = (F_o^2 + 2F_c^2)/3$
$(\Delta/\sigma)_{max}$ < 0.001
$\Delta\rho_{max}$ = 0.32 e Å^{-3}
$\Delta\rho_{min}$ = −0.39 e Å^{-3}

Special details

Geometry. All esds (except the esd in the dihedral angle between two l.s. planes) are estimated using the full covariance matrix. The cell esds are taken into account individually in the estimation of esds in distances, angles and torsion angles; correlations between esds in cell parameters are only used when they are defined by crystal symmetry. An approximate (isotropic) treatment of cell esds is used for estimating esds involving l.s. planes.

Fractional atomic coordinates and isotropic or equivalent isotropic displacement parameters (Å^2) for ***3***

	x	y	z	U_{iso}*/U_{eq}
C1	0.30957 (10)	0.26822 (19)	0.29807 (6)	0.0269 (3)
H1	0.273804	0.325912	0.267778	0.032*
F1	0.71202 (9)	−0.31400 (14)	0.18642 (4)	0.0574 (3)
N1	0.81892 (10)	0.0887 (2)	0.16597 (6)	0.0452 (4)
C2	0.38138 (10)	0.16809 (19)	0.27169 (6)	0.0278 (3)
F2	0.86743 (8)	−0.18491 (18)	0.16022 (6)	0.0706 (4)
N2	0.70975 (8)	0.90955 (19)	0.46569 (5)	0.0342 (3)
O2	0.44625 (6)	0.81146 (13)	0.43686 (4)	0.0284 (2)
C3	0.37115 (12)	0.1194 (2)	0.21683 (6)	0.0360 (4)
H3	0.318685	0.147613	0.196169	0.043*
F3	0.77201 (7)	0.36204 (13)	0.17142 (4)	0.0475 (3)
N3	0.10604 (9)	−0.2435 (2)	0.57625 (6)	0.0392 (3)
O3	0.07995 (7)	−0.16916 (15)	0.40861 (4)	0.0341 (3)
C4	0.43614 (13)	0.0307 (2)	0.19198 (7)	0.0429 (4)
H4	0.428931	−0.001427	0.154490	0.052*
F4	0.61075 (7)	0.26421 (12)	0.19272 (5)	0.0480 (3)
C5	0.51116 (12)	−0.0099 (2)	0.22249 (7)	0.0396 (4)
F5	0.56972 (6)	0.54019 (11)	0.45537 (4)	0.0360 (2)
C6	0.52356 (11)	0.0324 (2)	0.27729 (7)	0.0348 (4)
H6	0.575463	0.000231	0.297902	0.042*
F6	0.73857 (6)	0.62507 (14)	0.47453 (4)	0.0445 (3)
C7	0.45813 (10)	0.1231 (2)	0.30138 (6)	0.0301 (3)
H7	0.465896	0.155180	0.338852	0.036*
F7	0.68272 (6)	1.19498 (13)	0.45795 (4)	0.0415 (2)
C8	0.65406 (12)	−0.0324 (2)	0.18999 (6)	0.0371 (4)
F8	0.50938 (6)	1.13818 (11)	0.43672 (3)	0.0318 (2)
C9	0.72341 (13)	−0.1435 (2)	0.18203 (7)	0.0420 (4)
F9	0.09827 (6)	0.11117 (12)	0.48190 (4)	0.0374 (2)
C10	0.80186 (13)	−0.0765 (3)	0.16960 (7)	0.0460 (4)
F10	0.11261 (7)	0.04396 (15)	0.59077 (4)	0.0477 (3)
C11	0.75454 (11)	0.1934 (2)	0.17474 (6)	0.0370 (4)
F11	0.09810 (7)	−0.53176 (14)	0.56324 (4)	0.0459 (3)
C12	0.67166 (11)	0.1438 (2)	0.18627 (6)	0.0355 (4)
F12	0.08915 (6)	−0.49314 (12)	0.45273 (4)	0.0402 (2)
C13	0.34664 (9)	0.41190 (18)	0.33614 (6)	0.0255 (3)
C14	0.41636 (10)	0.51093 (19)	0.32047 (6)	0.0275 (3)
H14	0.440737	0.487224	0.286504	0.033*
C15	0.45122 (10)	0.64329 (19)	0.35307 (6)	0.0275 (3)
H15	0.498204	0.711297	0.341552	0.033*
C16	0.41593 (9)	0.67387 (18)	0.40274 (6)	0.0253 (3)
C17	0.34702 (9)	0.57933 (19)	0.41984 (6)	0.0267 (3)
H17	0.323711	0.602693	0.454150	0.032*

C18	0.31189 (9)	0.44893 (19)	0.38618 (6)	0.0266 (3)
H18	0.263571	0.384183	0.397401	0.032*
C19	0.53347 (9)	0.83670 (19)	0.44385 (5)	0.0256 (3)
C20	0.59406 (10)	0.7057 (2)	0.45428 (6)	0.0289 (3)
C21	0.68011 (10)	0.7509 (2)	0.46451 (6)	0.0323 (3)
C22	0.65227 (10)	1.0330 (2)	0.45627 (6)	0.0313 (3)
C23	0.56481 (10)	1.00523 (19)	0.44511 (6)	0.0274 (3)
C24	0.24900 (9)	0.14740 (18)	0.32676 (6)	0.0253 (3)
C25	0.28038 (10)	0.0469 (2)	0.37094 (6)	0.0280 (3)
H25	0.340784	0.049498	0.381867	0.034*
C26	0.22501 (9)	−0.05644 (19)	0.39915 (6)	0.0278 (3)
H26	0.246840	−0.123886	0.429420	0.033*
C27	0.13756 (9)	−0.06004 (19)	0.38261 (6)	0.0271 (3)
C28	0.10429 (10)	0.0353 (2)	0.33876 (6)	0.0304 (3)
H28	0.043965	0.030616	0.327720	0.036*
C29	0.16075 (10)	0.13873 (19)	0.31093 (6)	0.0278 (3)
H29	0.138544	0.204853	0.280453	0.033*
C30	0.09284 (9)	−0.1895 (2)	0.46382 (6)	0.0310 (3)
C31	0.09996 (10)	−0.0517 (2)	0.50026 (7)	0.0329 (3)
C32	0.10658 (10)	−0.0875 (2)	0.55554 (7)	0.0368 (4)
C33	0.09927 (10)	−0.3728 (2)	0.54176 (7)	0.0368 (4)
C34	0.09384 (10)	−0.3548 (2)	0.48570 (7)	0.0335 (3)
O1	0.57627 (9)	−0.10540 (16)	0.19760 (6)	0.0518 (3)

*Atomic displacement parameters (Å^2) for **3***

	U^{11}	U^{22}	U^{33}	U^{12}	U^{13}	U^{23}
C1	0.0327 (8)	0.0243 (7)	0.0228 (7)	−0.0023 (6)	−0.0062 (6)	0.0041 (6)
F1	0.0923 (9)	0.0315 (5)	0.0487 (6)	0.0101 (6)	0.0058 (6)	0.0004 (5)
N1	0.0413 (8)	0.0524 (10)	0.0416 (8)	0.0050 (7)	0.0012 (6)	−0.0015 (7)
C2	0.0381 (8)	0.0230 (7)	0.0223 (7)	−0.0094 (6)	0.0028 (6)	0.0020 (6)
F2	0.0594 (8)	0.0667 (8)	0.0848 (9)	0.0299 (7)	−0.0024 (6)	−0.0045 (7)
N2	0.0287 (7)	0.0430 (8)	0.0310 (7)	−0.0046 (6)	0.0018 (5)	−0.0028 (6)
O2	0.0260 (5)	0.0267 (5)	0.0319 (5)	−0.0007 (4)	−0.0027 (4)	−0.0069 (4)
C3	0.0526 (10)	0.0303 (8)	0.0248 (7)	−0.0165 (7)	0.0002 (7)	0.0003 (6)
F3	0.0474 (6)	0.0396 (6)	0.0564 (6)	−0.0105 (5)	0.0118 (5)	0.0003 (5)
N3	0.0285 (7)	0.0532 (9)	0.0361 (7)	−0.0007 (6)	0.0039 (6)	0.0026 (7)
O3	0.0310 (6)	0.0378 (6)	0.0330 (6)	−0.0117 (5)	−0.0021 (4)	0.0029 (5)
C4	0.0702 (12)	0.0343 (9)	0.0257 (8)	−0.0200 (9)	0.0138 (8)	−0.0079 (7)
F4	0.0525 (6)	0.0276 (5)	0.0672 (7)	−0.0020 (4)	0.0311 (5)	−0.0033 (5)
C5	0.0568 (11)	0.0235 (8)	0.0413 (9)	−0.0128 (7)	0.0256 (8)	−0.0075 (7)
F5	0.0358 (5)	0.0268 (5)	0.0445 (5)	0.0007 (4)	−0.0048 (4)	0.0027 (4)
C6	0.0410 (9)	0.0275 (8)	0.0369 (8)	−0.0045 (7)	0.0113 (7)	0.0000 (7)
F6	0.0305 (5)	0.0487 (6)	0.0538 (6)	0.0090 (4)	−0.0020 (4)	0.0043 (5)
C7	0.0376 (8)	0.0291 (8)	0.0239 (7)	−0.0022 (7)	0.0060 (6)	−0.0003 (6)
F7	0.0437 (5)	0.0368 (5)	0.0441 (5)	−0.0151 (4)	0.0033 (4)	−0.0048 (4)
C8	0.0529 (10)	0.0328 (9)	0.0273 (8)	−0.0044 (8)	0.0162 (7)	−0.0038 (7)
F8	0.0392 (5)	0.0257 (4)	0.0299 (4)	0.0013 (4)	−0.0016 (4)	−0.0023 (3)
C9	0.0670 (12)	0.0303 (8)	0.0290 (8)	0.0073 (8)	0.0033 (8)	−0.0003 (7)
F9	0.0314 (5)	0.0335 (5)	0.0474 (5)	−0.0009 (4)	0.0032 (4)	−0.0015 (4)
C10	0.0479 (11)	0.0489 (11)	0.0407 (10)	0.0160 (9)	−0.0008 (8)	−0.0003 (8)

F10	0.0441 (6)	0.0571 (7)	0.0425 (6)	−0.0008 (5)	0.0076 (4)	−0.0135 (5)
C11	0.0442 (9)	0.0371 (9)	0.0298 (8)	−0.0038 (8)	0.0040 (7)	0.0000 (7)
F11	0.0439 (6)	0.0478 (6)	0.0462 (6)	−0.0004 (5)	0.0045 (4)	0.0155 (5)
C12	0.0470 (10)	0.0304 (8)	0.0304 (8)	0.0010 (7)	0.0133 (7)	−0.0025 (7)
F12	0.0433 (5)	0.0335 (5)	0.0437 (5)	−0.0058 (4)	0.0030 (4)	0.0008 (4)
C13	0.0294 (7)	0.0207 (7)	0.0253 (7)	0.0000 (6)	−0.0059 (5)	0.0034 (6)
C14	0.0338 (8)	0.0265 (7)	0.0220 (7)	−0.0017 (6)	−0.0005 (6)	0.0021 (6)
C15	0.0298 (7)	0.0244 (7)	0.0281 (7)	−0.0038 (6)	−0.0004 (6)	0.0032 (6)
C16	0.0265 (7)	0.0207 (7)	0.0276 (7)	0.0011 (6)	−0.0056 (5)	−0.0010 (6)
C17	0.0275 (7)	0.0256 (7)	0.0267 (7)	0.0032 (6)	0.0004 (6)	0.0003 (6)
C18	0.0256 (7)	0.0238 (7)	0.0300 (7)	0.0004 (6)	−0.0010 (6)	0.0028 (6)
C19	0.0269 (7)	0.0300 (8)	0.0197 (6)	−0.0015 (6)	−0.0011 (5)	−0.0030 (6)
C20	0.0326 (8)	0.0276 (8)	0.0261 (7)	−0.0008 (6)	−0.0007 (6)	−0.0011 (6)
C21	0.0278 (8)	0.0399 (9)	0.0290 (7)	0.0049 (7)	0.0002 (6)	0.0002 (7)
C22	0.0364 (8)	0.0331 (8)	0.0245 (7)	−0.0089 (7)	0.0025 (6)	−0.0035 (6)
C23	0.0341 (8)	0.0277 (7)	0.0202 (7)	0.0001 (6)	0.0005 (6)	−0.0019 (6)
C24	0.0298 (7)	0.0199 (7)	0.0256 (7)	−0.0008 (6)	−0.0029 (6)	−0.0015 (6)
C25	0.0245 (7)	0.0290 (8)	0.0300 (7)	0.0005 (6)	−0.0028 (6)	0.0038 (6)
C26	0.0279 (7)	0.0258 (7)	0.0295 (7)	0.0024 (6)	−0.0011 (6)	0.0040 (6)
C27	0.0277 (7)	0.0234 (7)	0.0301 (7)	−0.0034 (6)	0.0018 (6)	−0.0014 (6)
C28	0.0264 (7)	0.0311 (8)	0.0326 (8)	−0.0011 (6)	−0.0064 (6)	−0.0028 (6)
C29	0.0329 (8)	0.0229 (7)	0.0267 (7)	0.0018 (6)	−0.0067 (6)	−0.0005 (6)
C30	0.0206 (7)	0.0388 (9)	0.0335 (8)	−0.0038 (6)	0.0014 (6)	0.0019 (7)
C31	0.0222 (7)	0.0354 (9)	0.0412 (9)	−0.0014 (6)	0.0025 (6)	0.0007 (7)
C32	0.0251 (8)	0.0474 (10)	0.0383 (9)	−0.0016 (7)	0.0045 (6)	−0.0060 (8)
C33	0.0263 (8)	0.0430 (9)	0.0413 (9)	−0.0009 (7)	0.0038 (6)	0.0098 (8)
C34	0.0262 (7)	0.0369 (9)	0.0373 (8)	−0.0049 (7)	0.0028 (6)	0.0005 (7)
O1	0.0657 (7)	0.0360 (6)	0.0576 (6)	−0.0084 (5)	0.0352 (6)	−0.0116 (5)

*Geometric parameters (Å, º) for **3***

C1—H1	1.0000	F8—C23	1.3431 (17)
C1—C2	1.524 (2)	C9—C10	1.364 (3)
C1—C13	1.537 (2)	F9—C31	1.3373 (19)
C1—C24	1.521 (2)	F10—C32	1.333 (2)
F1—C9	1.336 (2)	C11—C12	1.376 (2)
N1—C10	1.309 (3)	F11—C33	1.338 (2)
N1—C11	1.306 (2)	F12—C34	1.3393 (19)
C2—C3	1.393 (2)	C13—C14	1.390 (2)
C2—C7	1.390 (2)	C13—C18	1.395 (2)
F2—C10	1.342 (2)	C14—H14	0.9500
N2—C21	1.309 (2)	C14—C15	1.385 (2)
N2—C22	1.311 (2)	C15—H15	0.9500
O2—C16	1.4139 (17)	C15—C16	1.381 (2)
O2—C19	1.3546 (17)	C16—C17	1.373 (2)
C3—H3	0.9500	C17—H17	0.9500
C3—C4	1.383 (3)	C17—C18	1.390 (2)
F3—C11	1.335 (2)	C18—H18	0.9500
N3—C32	1.309 (2)	C19—C20	1.389 (2)
N3—C33	1.309 (2)	C19—C23	1.390 (2)
O3—C27	1.4037 (18)	C20—C21	1.375 (2)

O3—C30	1.3622 (18)	C22—C23	1.371 (2)
C4—H4	0.9500	C24—C25	1.393 (2)
C4—C5	1.371 (3)	C24—C29	1.389 (2)
F4—C12	1.3367 (19)	C25—H25	0.9500
C5—C6	1.382 (2)	C25—C26	1.383 (2)
C5—O1	1.412 (2)	C26—H26	0.9500
F5—C20	1.3347 (18)	C26—C27	1.379 (2)
C6—H6	0.9500	C27—C28	1.374 (2)
C6—C7	1.387 (2)	C28—H28	0.9500
F6—C21	1.3368 (18)	C28—C29	1.389 (2)
C7—H7	0.9500	C29—H29	0.9500
F7—C22	1.3377 (18)	C30—C31	1.390 (2)
C8—C9	1.392 (3)	C30—C34	1.386 (2)
C8—C12	1.394 (2)	C31—C32	1.378 (2)
C8—O1	1.345 (2)	C33—C34	1.376 (2)

C2—C1—H1	107.0	C16—C17—H17	120.5
C2—C1—C13	111.93 (12)	C16—C17—C18	119.00 (14)
C13—C1—H1	107.0	C18—C17—H17	120.5
C24—C1—H1	107.0	C13—C18—H18	119.6
C24—C1—C2	111.26 (12)	C17—C18—C13	120.87 (14)
C24—C1—C13	112.29 (12)	C17—C18—H18	119.6
C11—N1—C10	115.94 (17)	O2—C19—C20	124.37 (14)
C3—C2—C1	120.25 (14)	O2—C19—C23	118.47 (13)
C7—C2—C1	121.26 (13)	C20—C19—C23	116.95 (13)
C7—C2—C3	118.50 (15)	F5—C20—C19	121.21 (13)
C21—N2—C22	116.71 (13)	F5—C20—C21	120.51 (14)
C19—O2—C16	118.06 (11)	C21—C20—C19	118.27 (14)
C2—C3—H3	119.5	N2—C21—F6	116.73 (14)
C4—C3—C2	121.06 (16)	N2—C21—C20	124.86 (15)
C4—C3—H3	119.5	F6—C21—C20	118.40 (15)
C33—N3—C32	117.22 (14)	N2—C22—F7	116.52 (14)
C30—O3—C27	117.49 (11)	N2—C22—C23	124.19 (15)
C3—C4—H4	120.7	F7—C22—C23	119.29 (15)
C5—C4—C3	118.70 (15)	F8—C23—C19	119.98 (13)
C5—C4—H4	120.7	F8—C23—C22	120.98 (14)
C4—C5—C6	122.35 (16)	C22—C23—C19	119.02 (14)
C4—C5—O1	118.66 (15)	C25—C24—C1	120.57 (13)
C6—C5—O1	118.93 (18)	C29—C24—C1	121.05 (13)
C5—C6—H6	120.9	C29—C24—C25	118.35 (14)
C5—C6—C7	118.16 (16)	C24—C25—H25	119.5
C7—C6—H6	120.9	C26—C25—C24	121.05 (13)
C2—C7—H7	119.4	C26—C25—H25	119.5
C6—C7—C2	121.22 (14)	C25—C26—H26	120.5
C6—C7—H7	119.4	C27—C26—C25	118.91 (13)
C9—C8—C12	116.20 (16)	C27—C26—H26	120.5
O1—C8—C9	117.04 (15)	C26—C27—O3	120.62 (13)
O1—C8—C12	126.66 (16)	C28—C27—O3	117.51 (13)
F1—C9—C8	119.55 (18)	C28—C27—C26	121.79 (14)
F1—C9—C10	121.04 (17)	C27—C28—H28	120.7
C10—C9—C8	119.41 (16)	C27—C28—C29	118.58 (13)

N1—C10—F2	116.32 (18)	C29—C28—H28	120.7
N1—C10—C9	124.75 (17)	C24—C29—C28	121.31 (13)
F2—C10—C9	118.92 (18)	C24—C29—H29	119.3
N1—C11—F3	116.02 (16)	C28—C29—H29	119.3
N1—C11—C12	125.48 (17)	O3—C30—C31	123.22 (15)
F3—C11—C12	118.49 (16)	O3—C30—C34	119.15 (15)
F4—C12—C8	122.28 (15)	C34—C30—C31	117.51 (14)
F4—C12—C11	119.55 (15)	F9—C31—C30	120.57 (14)
C11—C12—C8	118.17 (16)	F9—C31—C32	121.19 (15)
C14—C13—C1	119.64 (13)	C32—C31—C30	118.22 (16)
C14—C13—C18	118.17 (13)	N3—C32—F10	117.09 (15)
C18—C13—C1	122.19 (13)	N3—C32—C31	124.30 (16)
C13—C14—H14	119.1	F10—C32—C31	118.61 (16)
C15—C14—C13	121.72 (14)	N3—C33—F11	116.87 (15)
C15—C14—H14	119.1	N3—C33—C34	124.24 (16)
C14—C15—H15	120.8	F11—C33—C34	118.89 (16)
C16—C15—C14	118.32 (14)	F12—C34—C30	120.39 (14)
C16—C15—H15	120.8	F12—C34—C33	121.13 (15)
C15—C16—O2	121.02 (13)	C33—C34—C30	118.48 (16)
C17—C16—O2	116.97 (13)	C8—O1—C5	119.96 (13)
C17—C16—C15	121.91 (13)		

C1—C2—C3—C4	179.04 (14)	C12—C8—C9—C10	−2.1 (2)
C1—C2—C7—C6	−179.82 (14)	C12—C8—O1—C5	−23.7 (3)
C1—C13—C14—C15	179.13 (13)	C13—C1—C2—C3	−139.20 (14)
C1—C13—C18—C17	179.78 (13)	C13—C1—C2—C7	40.83 (18)
C1—C24—C25—C26	176.55 (14)	C13—C1—C24—C25	−63.37 (18)
C1—C24—C29—C28	−176.66 (14)	C13—C1—C24—C29	114.39 (15)
F1—C9—C10—N1	−178.13 (16)	C13—C14—C15—C16	1.1 (2)
F1—C9—C10—F2	1.9 (3)	C14—C13—C18—C17	−1.1 (2)
N1—C11—C12—F4	−177.89 (15)	C14—C15—C16—O2	−177.42 (12)
N1—C11—C12—C8	1.2 (3)	C14—C15—C16—C17	−1.2 (2)
C2—C1—C13—C14	40.65 (17)	C15—C16—C17—C18	0.1 (2)
C2—C1—C13—C18	−140.23 (14)	C16—O2—C19—C20	−45.14 (19)
C2—C1—C24—C25	62.98 (17)	C16—O2—C19—C23	140.26 (13)
C2—C1—C24—C29	−119.27 (15)	C16—C17—C18—C13	1.1 (2)
C2—C3—C4—C5	0.4 (2)	C18—C13—C14—C15	0.0 (2)
N2—C22—C23—F8	178.92 (13)	C19—O2—C16—C15	−43.81 (18)
N2—C22—C23—C19	0.4 (2)	C19—O2—C16—C17	139.76 (13)
O2—C16—C17—C18	176.49 (12)	C19—C20—C21—N2	0.6 (2)
O2—C19—C20—F5	3.6 (2)	C19—C20—C21—F6	−179.95 (13)
O2—C19—C20—C21	−175.30 (13)	C20—C19—C23—F8	−178.36 (12)
O2—C19—C23—F8	−3.4 (2)	C20—C19—C23—C22	0.1 (2)
O2—C19—C23—C22	175.14 (12)	C21—N2—C22—F7	179.48 (13)
C3—C2—C7—C6	0.2 (2)	C21—N2—C22—C23	−0.5 (2)
C3—C4—C5—C6	0.9 (2)	C22—N2—C21—F6	−179.51 (13)
C3—C4—C5—O1	177.94 (14)	C22—N2—C21—C20	−0.1 (2)
F3—C11—C12—F4	1.3 (2)	C23—C19—C20—F5	178.23 (13)
F3—C11—C12—C8	−179.62 (15)	C23—C19—C20—C21	−0.6 (2)
N3—C33—C34—F12	178.36 (14)	C24—C1—C2—C3	94.26 (16)
N3—C33—C34—C30	−2.0 (2)	C24—C1—C2—C7	−85.71 (16)

O3—C27—C28—C29	−177.33 (13)	C24—C1—C13—C14	166.63 (13)
O3—C30—C31—F9	−3.1 (2)	C24—C1—C13—C18	−14.25 (19)
O3—C30—C31—C32	175.35 (13)	C24—C25—C26—C27	0.5 (2)
O3—C30—C34—F12	5.3 (2)	C25—C24—C29—C28	1.1 (2)
O3—C30—C34—C33	−174.39 (13)	C25—C26—C27—O3	177.12 (13)
C4—C5—C6—C7	−1.7 (2)	C25—C26—C27—C28	0.4 (2)
C4—C5—O1—C8	114.35 (18)	C26—C27—C28—C29	−0.5 (2)
C5—C6—C7—C2	1.1 (2)	C27—O3—C30—C31	52.6 (2)
F5—C20—C21—N2	−178.22 (14)	C27—O3—C30—C34	−131.44 (15)
F5—C20—C21—F6	1.2 (2)	C27—C28—C29—C24	−0.3 (2)
C6—C5—O1—C8	−68.5 (2)	C29—C24—C25—C26	−1.3 (2)
C7—C2—C3—C4	−1.0 (2)	C30—O3—C27—C26	37.8 (2)
F7—C22—C23—F8	−1.0 (2)	C30—O3—C27—C28	−145.35 (14)
F7—C22—C23—C19	−179.50 (13)	C30—C31—C32—N3	−0.5 (2)
C8—C9—C10—N1	2.2 (3)	C30—C31—C32—F10	−179.38 (13)
C8—C9—C10—F2	−177.81 (16)	C31—C30—C34—F12	−178.53 (13)
C9—C8—C12—F4	179.59 (15)	C31—C30—C34—C33	1.8 (2)
C9—C8—C12—C11	0.6 (2)	C32—N3—C33—F11	−179.50 (14)
C9—C8—O1—C5	160.07 (16)	C32—N3—C33—C34	0.8 (2)
F9—C31—C32—N3	177.93 (14)	C33—N3—C32—F10	179.35 (14)
F9—C31—C32—F10	−1.0 (2)	C33—N3—C32—C31	0.4 (2)
C10—N1—C11—F3	179.55 (15)	C34—C30—C31—F9	−179.10 (13)
C10—N1—C11—C12	−1.2 (3)	C34—C30—C31—C32	−0.7 (2)
C11—N1—C10—F2	179.49 (15)	O1—C5—C6—C7	−178.69 (14)
C11—N1—C10—C9	−0.5 (3)	O1—C8—C9—F1	−5.1 (2)
F11—C33—C34—F12	−1.3 (2)	O1—C8—C9—C10	174.56 (16)
F11—C33—C34—C30	178.36 (14)	O1—C8—C12—F4	3.3 (3)
C12—C8—C9—F1	178.24 (15)	O1—C8—C12—C11	−175.72 (16)

Hydrogen-bond geometry (Å, º) for ***3***

D—H···*A*	*D*—H	H···*A*	*D*···*A*	*D*—H···*A*
C4—H4···F12^{i}	0.95	2.62	3.5420 (19)	163

Symmetry code: (i) −*x*+1/2, *y*+1/2, −*z*+1/2.

6 (CCDC #: 2486388)

Computing details

Data collection: *CrysAlis PRO* system (CCD 43.144a 64-bit (release 23-01-2025)); cell refinement: *CrysAlis PRO* 1.171.43.144a (Rigaku OD, 2024); data reduction: *CrysAlis PRO* 1.171.43.144a (Rigaku OD, 2024); program(s) used to solve structure: SHELXT 2018/2 (Sheldrick, 2018); program(s) used to refine structure: *SHELXL* 2018/3 (Sheldrick, 2015); molecular graphics: Olex2 1.5-ac5-023 (Dolomanov *et al.*, 2009); software used to prepare material for publication: Olex2 1.5-ac5-023 (Dolomanov *et al.*, 2009).

Crystal data

$C_{35}H_{16}F_8N_2O_2$
M_r = 648.50
Triclinic, $P\overline{1}$
a = 11.2042 (1) Å
b = 14.6898 (2) Å
c = 18.0437 (2) Å
α = 68.121 (1)°
β = 89.738 (1)°
γ = 78.704 (1)°
V = 2694.83 (6) Å^3
Z = 4
$F(000)$ = 1312
D_x = 1.598 Mg m^{-3}
Melting point: 440 K
Cu $K\alpha$ radiation, λ = 1.54184 Å
Cell parameters from 35165 reflections
θ = 2.6–68.3°
μ = 1.20 mm^{-1}
T = 105 K
Rect. Prism, clear yellow
0.48 × 0.15 × 0.13 mm

Data collection

XtaLAB Synergy, Dualflex, HyPix3000 diffractometer
Radiation source: micro-focus sealed X-ray tube, PhotonJet (Cu) X-ray Source
Mirror monochromator
Detector resolution: 10.0000 pixels mm^{-1}
ω scans
Absorption correction: gaussian *CrysAlis PRO* 1.171.43.144a (Rigaku Oxford Diffraction, 2024) Numerical absorption correction based on gaussian integration over a multifaceted crystal model Empirical absorption correction using spherical harmonics, implemented in SCALE3 ABSPACK scaling algorithm.
T_{min} = 0.448, T_{max} = 1.000
52314 measured reflections
9876 independent reflections
9261 reflections with $I > 2\sigma(I)$
R_{int} = 0.028
θ_{max} = 68.4°, θ_{min} = 2.7°
h = −13→13
k = −17→17
l = −17→21

Refinement

Refinement on F^2
Least-squares matrix: full
$R[F^2 > 2\sigma(F^2)]$ = 0.039
$wR(F^2)$ = 0.105
S = 1.03
9876 reflections
847 parameters
0 restraints
Primary atom site location: dual
Hydrogen site location: inferred from neighbouring sites
H-atom parameters constrained
$w = 1/[\sigma^2(F_o^2) + (0.0584P)^2 + 1.5907P]$ where $P = (F_o^2 + 2F_c^2)/3$
$(\Delta/\sigma)_{max}$ = 0.001
$\Delta\rho_{max}$ = 0.42 e Å^{-3}
$\Delta\rho_{min}$ = −0.24 e Å^{-3}

Special details

Geometry. All esds (except the esd in the dihedral angle between two l.s. planes) are estimated using the full covariance matrix. The cell esds are taken into account individually in the estimation of esds in distances, angles and torsion angles; correlations between esds in cell parameters are only used when they are defined by crystal symmetry. An approximate (isotropic) treatment of cell esds is used for estimating esds involving l.s. planes.

Fractional atomic coordinates and isotropic or equivalent isotropic displacement parameters (Å^2) for ***6***

	x	y	z	U_{iso}*/U_{eq}
F1	0.00211 (8)	0.11848 (7)	0.66266 (5)	0.0247 (2)
F16	0.14059 (8)	0.19936 (7)	0.53479 (5)	0.0247 (2)
F8	0.51597 (8)	0.19960 (7)	0.03353 (6)	0.0263 (2)
F2	0.01344 (9)	−0.08004 (7)	0.72593 (6)	0.0287 (2)
F5	0.16605 (8)	0.12127 (7)	0.17291 (6)	0.0271 (2)
F12	0.68361 (9)	0.10413 (8)	1.16883 (6)	0.0290 (2)
F13	0.53069 (9)	0.11655 (7)	0.67370 (6)	0.0297 (2)
F15	0.15796 (9)	0.00048 (8)	0.57422 (6)	0.0306 (2)
F9	1.00658 (9)	0.16697 (8)	0.99967 (6)	0.0304 (2)
F6	0.26472 (9)	−0.07000 (7)	0.20848 (6)	0.0312 (2)
F4	−0.34011 (9)	0.14721 (8)	0.49714 (6)	0.0339 (2)
F11	0.80033 (10)	−0.08943 (8)	1.23136 (6)	0.0345 (2)
F14	0.52343 (10)	−0.07458 (8)	0.71131 (6)	0.0355 (2)
F10	1.11095 (9)	−0.02806 (8)	1.07191 (6)	0.0354 (3)
F7	0.59137 (9)	0.00082 (8)	0.06719 (7)	0.0374 (3)
F3	−0.31502 (11)	−0.05349 (9)	0.56963 (6)	0.0389 (3)
O3	0.78182 (11)	0.24044 (8)	1.04698 (6)	0.0238 (2)
O1	−0.17430 (11)	0.24076 (8)	0.54238 (6)	0.0231 (2)
O2	0.30869 (10)	0.27026 (8)	0.08326 (7)	0.0228 (2)
O4	0.31568 (10)	0.26815 (8)	0.58177 (7)	0.0244 (2)
N1	−0.15151 (13)	−0.06622 (10)	0.64791 (8)	0.0249 (3)
N3	0.95593 (13)	−0.05863 (10)	1.15219 (8)	0.0260 (3)
N4	0.34048 (13)	−0.03741 (11)	0.64306 (8)	0.0269 (3)
N2	0.42760 (13)	−0.03533 (11)	0.13738 (9)	0.0284 (3)
C43	0.69550 (13)	0.59216 (11)	0.63851 (8)	0.0161 (3)
C42	0.80065 (13)	0.51887 (11)	0.63236 (8)	0.0170 (3)
C20	−0.00284 (13)	0.38639 (11)	0.17474 (9)	0.0167 (3)
C13	−0.13134 (13)	0.54408 (11)	0.17947 (8)	0.0162 (3)
C15	−0.18430 (13)	0.43209 (11)	0.34226 (9)	0.0178 (3)
H15	−0.215072	0.502231	0.316300	0.021*
C48	0.60853 (13)	0.54139 (11)	0.68119 (8)	0.0161 (3)
C7	−0.31004 (13)	0.51972 (11)	0.13086 (9)	0.0179 (3)
C37	0.77803 (13)	0.42244 (11)	0.67165 (8)	0.0166 (3)
C8	−0.24421 (13)	0.59394 (11)	0.13720 (9)	0.0171 (3)
C56	0.46459 (13)	0.35336 (11)	0.71768 (9)	0.0186 (3)
H56	0.453556	0.358812	0.768209	0.022*
C55	0.56384 (13)	0.38363 (11)	0.67572 (9)	0.0168 (3)
C50	0.71609 (13)	0.42976 (11)	0.84404 (9)	0.0178 (3)
H50	0.714651	0.499315	0.817508	0.021*
C2	−0.23694 (13)	0.42378 (11)	0.16992 (8)	0.0168 (3)
C49	0.68230 (12)	0.37656 (11)	0.80036 (9)	0.0154 (3)
C54	0.68663 (13)	0.27429 (11)	0.84105 (9)	0.0184 (3)

H54	0.665996	0.236144	0.812402	0.022*
C14	−0.12223 (13)	0.37907 (11)	0.29857 (9)	0.0157 (3)
C22	0.21477 (14)	0.31735 (11)	0.18762 (9)	0.0200 (3)
H22	0.291811	0.296294	0.217218	0.024*
C57	0.38132 (14)	0.31522 (11)	0.68666 (9)	0.0205 (3)
H57	0.313847	0.294533	0.715460	0.025*
C52	0.75305 (13)	0.28251 (11)	0.96329 (9)	0.0187 (3)
C19	−0.07811 (14)	0.27646 (11)	0.33869 (9)	0.0192 (3)
H19	−0.036061	0.238628	0.310230	0.023*
C17	−0.15706 (14)	0.28308 (11)	0.46009 (9)	0.0181 (3)
C36	0.65455 (13)	0.42748 (11)	0.70885 (9)	0.0159 (3)
C21	0.11061 (14)	0.35549 (11)	0.21780 (9)	0.0188 (3)
H21	0.116900	0.360570	0.268544	0.023*
C61	0.83945 (14)	0.14269 (11)	1.07987 (9)	0.0193 (3)
C47	0.49817 (14)	0.59323 (11)	0.69368 (9)	0.0185 (3)
H47	0.438935	0.558314	0.722247	0.022*
C32	0.27488 (14)	0.09787 (12)	0.14620 (9)	0.0207 (3)
C27	−0.07992 (14)	0.07976 (12)	0.63708 (9)	0.0187 (3)
C70	0.23900 (14)	0.13338 (12)	0.57866 (9)	0.0208 (3)
C3	−0.27748 (14)	0.33869 (12)	0.17460 (9)	0.0192 (3)
H3	−0.228033	0.273760	0.201457	0.023*
C26	−0.16815 (14)	0.14031 (11)	0.57581 (9)	0.0190 (3)
C28	−0.07575 (14)	−0.02161 (12)	0.66952 (9)	0.0208 (3)
C65	0.78998 (14)	0.07494 (12)	1.14176 (9)	0.0214 (3)
C1	−0.11741 (13)	0.43009 (11)	0.20725 (9)	0.0162 (3)
C23	0.20355 (14)	0.31084 (11)	0.11387 (9)	0.0196 (3)
C44	0.67398 (14)	0.69681 (11)	0.60980 (9)	0.0192 (3)
H44	0.733750	0.731748	0.582055	0.023*
C66	0.33242 (14)	0.16818 (12)	0.60378 (9)	0.0201 (3)
C12	−0.04956 (14)	0.59721 (11)	0.19240 (9)	0.0191 (3)
H12	0.027584	0.563193	0.220718	0.023*
C16	−0.20207 (14)	0.38499 (11)	0.42238 (9)	0.0193 (3)
H16	−0.244683	0.422268	0.451072	0.023*
C62	0.95125 (14)	0.10619 (12)	1.05727 (9)	0.0214 (3)
C53	0.72037 (14)	0.22723 (11)	0.92236 (9)	0.0200 (3)
H53	0.720994	0.157911	0.949540	0.024*
C38	0.86289 (14)	0.33804 (12)	0.67586 (9)	0.0192 (3)
H38	0.847499	0.272790	0.703030	0.023*
C41	0.90961 (14)	0.53158 (12)	0.59624 (9)	0.0203 (3)
H41	0.925487	0.596747	0.569505	0.024*
C31	0.33902 (14)	0.17048 (12)	0.10275 (9)	0.0194 (3)
C64	0.85137 (15)	−0.02371 (12)	1.17425 (9)	0.0237 (3)
C45	0.56359 (14)	0.74863 (11)	0.62262 (9)	0.0211 (3)
H45	0.547657	0.819810	0.603287	0.025*
C9	−0.27848 (14)	0.69831 (12)	0.10922 (9)	0.0205 (3)
H9	−0.356111	0.732317	0.081621	0.025*
C40	0.99459 (14)	0.44678 (12)	0.60019 (9)	0.0214 (3)
H40	1.069371	0.454208	0.575922	0.026*
C51	0.75162 (14)	0.38348 (11)	0.92511 (9)	0.0197 (3)
H51	0.774616	0.420626	0.953941	0.024*
C18	−0.09403 (14)	0.22804 (11)	0.41924 (9)	0.0204 (3)

H18	−0.062116	0.158173	0.445883	0.025*
C63	1.00388 (15)	0.00624 (13)	1.09526 (10)	0.0244 (3)
C6	−0.42495 (14)	0.53113 (12)	0.09540 (9)	0.0210 (3)
H6	−0.474731	0.595927	0.068597	0.025*
C35	0.44873 (14)	0.13476 (12)	0.07607 (9)	0.0218 (3)
C25	−0.00877 (14)	0.38225 (12)	0.09889 (9)	0.0217 (3)
H25	−0.084880	0.405340	0.068030	0.026*
C58	0.39917 (14)	0.30825 (12)	0.61331 (10)	0.0211 (3)
C11	−0.08284 (15)	0.70162 (12)	0.16298 (9)	0.0222 (3)
H11	−0.027267	0.739182	0.170554	0.027*
C4	−0.39272 (14)	0.35070 (12)	0.13891 (9)	0.0220 (3)
H4	−0.421886	0.293248	0.141209	0.026*
C30	−0.25060 (15)	0.09362 (13)	0.55409 (9)	0.0235 (3)
C46	0.47571 (14)	0.69782 (12)	0.66345 (9)	0.0207 (3)
H46	0.399967	0.734644	0.670755	0.025*
C39	0.97164 (14)	0.35112 (12)	0.63919 (9)	0.0214 (3)
H39	1.030620	0.294154	0.640874	0.026*
C69	0.24878 (15)	0.03224 (13)	0.59960 (10)	0.0234 (3)
C67	0.43184 (14)	0.09450 (12)	0.64807 (9)	0.0224 (3)
C5	−0.46525 (14)	0.44550 (13)	0.10009 (9)	0.0228 (3)
H5	−0.543583	0.452039	0.076367	0.027*
C10	−0.19673 (15)	0.75139 (12)	0.12259 (9)	0.0224 (3)
H10	−0.218712	0.822471	0.103971	0.027*
C24	0.09426 (14)	0.34507 (12)	0.06756 (9)	0.0229 (3)
H24	0.089660	0.343237	0.015590	0.028*
C33	0.32522 (15)	−0.00172 (12)	0.16270 (10)	0.0240 (3)
C34	0.48633 (15)	0.03364 (13)	0.09421 (10)	0.0264 (4)
C68	0.42863 (15)	−0.00490 (13)	0.66624 (10)	0.0258 (3)
C60	0.57514 (15)	0.37888 (12)	0.60007 (9)	0.0225 (3)
H60	0.640690	0.401514	0.569955	0.027*
C29	−0.23698 (16)	−0.00851 (13)	0.59193 (10)	0.0254 (3)
C59	0.49252 (15)	0.34174 (13)	0.56795 (10)	0.0249 (3)
H59	0.500126	0.339494	0.516170	0.030*

Atomic displacement parameters (Å^2) for ***6***

	U^{11}	U^{22}	U^{33}	U^{12}	U^{13}	U^{23}
F1	0.0257 (5)	0.0271 (5)	0.0231 (5)	−0.0112 (4)	0.0001 (4)	−0.0086 (4)
F16	0.0196 (4)	0.0301 (5)	0.0236 (5)	−0.0080 (4)	−0.0018 (4)	−0.0079 (4)
F8	0.0176 (4)	0.0335 (5)	0.0242 (5)	−0.0033 (4)	0.0047 (4)	−0.0080 (4)
F2	0.0288 (5)	0.0233 (5)	0.0255 (5)	0.0018 (4)	−0.0043 (4)	−0.0034 (4)
F5	0.0237 (5)	0.0265 (5)	0.0315 (5)	−0.0078 (4)	0.0098 (4)	−0.0103 (4)
F12	0.0214 (5)	0.0392 (6)	0.0212 (5)	−0.0030 (4)	0.0037 (4)	−0.0074 (4)
F13	0.0222 (5)	0.0325 (5)	0.0347 (5)	−0.0040 (4)	−0.0080 (4)	−0.0137 (4)
F15	0.0307 (5)	0.0363 (5)	0.0355 (6)	−0.0185 (4)	0.0062 (4)	−0.0198 (5)
F9	0.0274 (5)	0.0352 (5)	0.0245 (5)	−0.0076 (4)	0.0080 (4)	−0.0062 (4)
F6	0.0375 (6)	0.0225 (5)	0.0325 (5)	−0.0112 (4)	0.0008 (4)	−0.0069 (4)
F4	0.0308 (5)	0.0385 (6)	0.0260 (5)	−0.0074 (4)	−0.0099 (4)	−0.0047 (4)
F11	0.0452 (6)	0.0299 (5)	0.0242 (5)	−0.0175 (5)	0.0015 (4)	−0.0008 (4)
F14	0.0367 (6)	0.0284 (5)	0.0360 (6)	0.0054 (4)	−0.0083 (5)	−0.0120 (5)
F10	0.0268 (5)	0.0442 (6)	0.0300 (5)	0.0111 (4)	−0.0025 (4)	−0.0176 (5)

F7	0.0267 (5)	0.0400 (6)	0.0431 (6)	0.0077 (4)	0.0049 (5)	−0.0205 (5)
F3	0.0498 (7)	0.0455 (6)	0.0301 (5)	−0.0333 (5)	0.0017 (5)	−0.0127 (5)
O3	0.0320 (6)	0.0195 (5)	0.0145 (5)	0.0042 (5)	−0.0017 (4)	−0.0052 (4)
O1	0.0371 (6)	0.0165 (5)	0.0148 (5)	−0.0067 (5)	0.0044 (5)	−0.0044 (4)
O2	0.0199 (5)	0.0199 (5)	0.0271 (6)	−0.0025 (4)	0.0088 (4)	−0.0081 (5)
O4	0.0233 (6)	0.0229 (6)	0.0279 (6)	−0.0081 (5)	−0.0063 (5)	−0.0090 (5)
N1	0.0334 (8)	0.0226 (7)	0.0202 (7)	−0.0106 (6)	0.0059 (6)	−0.0074 (6)
N3	0.0345 (8)	0.0221 (7)	0.0193 (7)	−0.0009 (6)	−0.0067 (6)	−0.0077 (6)
N4	0.0326 (8)	0.0260 (7)	0.0257 (7)	−0.0075 (6)	0.0052 (6)	−0.0131 (6)
N2	0.0294 (8)	0.0257 (7)	0.0290 (8)	0.0008 (6)	−0.0036 (6)	−0.0122 (6)
C43	0.0159 (7)	0.0197 (7)	0.0129 (7)	−0.0038 (6)	−0.0015 (5)	−0.0062 (6)
C42	0.0176 (7)	0.0201 (7)	0.0135 (7)	−0.0045 (6)	−0.0012 (5)	−0.0064 (6)
C20	0.0166 (7)	0.0147 (7)	0.0176 (7)	−0.0035 (5)	0.0021 (6)	−0.0046 (6)
C13	0.0179 (7)	0.0173 (7)	0.0121 (7)	−0.0030 (6)	0.0041 (5)	−0.0044 (6)
C15	0.0167 (7)	0.0158 (7)	0.0200 (7)	−0.0021 (6)	0.0008 (6)	−0.0064 (6)
C48	0.0175 (7)	0.0173 (7)	0.0130 (7)	−0.0042 (6)	−0.0016 (5)	−0.0049 (6)
C7	0.0174 (7)	0.0220 (7)	0.0139 (7)	−0.0033 (6)	0.0034 (5)	−0.0067 (6)
C37	0.0169 (7)	0.0218 (7)	0.0135 (7)	−0.0065 (6)	0.0008 (5)	−0.0081 (6)
C8	0.0176 (7)	0.0204 (7)	0.0132 (7)	−0.0045 (6)	0.0042 (5)	−0.0062 (6)
C56	0.0180 (7)	0.0207 (7)	0.0175 (7)	−0.0045 (6)	0.0024 (6)	−0.0076 (6)
C55	0.0169 (7)	0.0147 (7)	0.0180 (7)	−0.0031 (5)	0.0002 (5)	−0.0054 (6)
C50	0.0176 (7)	0.0159 (7)	0.0201 (7)	−0.0044 (6)	0.0016 (6)	−0.0065 (6)
C2	0.0150 (7)	0.0225 (7)	0.0134 (7)	−0.0035 (6)	0.0023 (5)	−0.0078 (6)
C49	0.0119 (6)	0.0170 (7)	0.0166 (7)	−0.0021 (5)	0.0022 (5)	−0.0059 (6)
C54	0.0188 (7)	0.0167 (7)	0.0208 (7)	−0.0026 (6)	0.0018 (6)	−0.0088 (6)
C14	0.0135 (7)	0.0174 (7)	0.0161 (7)	−0.0043 (5)	0.0001 (5)	−0.0056 (6)
C22	0.0161 (7)	0.0207 (7)	0.0213 (8)	−0.0024 (6)	−0.0010 (6)	−0.0064 (6)
C57	0.0179 (7)	0.0217 (8)	0.0221 (8)	−0.0070 (6)	0.0031 (6)	−0.0071 (6)
C52	0.0173 (7)	0.0211 (7)	0.0137 (7)	0.0012 (6)	0.0004 (5)	−0.0049 (6)
C19	0.0205 (7)	0.0180 (7)	0.0204 (8)	−0.0028 (6)	0.0020 (6)	−0.0095 (6)
C17	0.0199 (7)	0.0200 (7)	0.0139 (7)	−0.0068 (6)	0.0010 (6)	−0.0044 (6)
C36	0.0158 (7)	0.0163 (7)	0.0162 (7)	−0.0046 (6)	0.0023 (5)	−0.0062 (6)
C21	0.0186 (7)	0.0200 (7)	0.0178 (7)	−0.0033 (6)	0.0004 (6)	−0.0074 (6)
C61	0.0217 (7)	0.0189 (7)	0.0152 (7)	−0.0006 (6)	−0.0042 (6)	−0.0060 (6)
C47	0.0178 (7)	0.0207 (7)	0.0161 (7)	−0.0049 (6)	0.0012 (6)	−0.0053 (6)
C32	0.0190 (7)	0.0250 (8)	0.0185 (7)	−0.0043 (6)	0.0009 (6)	−0.0090 (6)
C27	0.0187 (7)	0.0227 (8)	0.0166 (7)	−0.0070 (6)	0.0043 (6)	−0.0081 (6)
C70	0.0182 (7)	0.0273 (8)	0.0185 (7)	−0.0076 (6)	0.0034 (6)	−0.0090 (6)
C3	0.0185 (7)	0.0217 (8)	0.0196 (7)	−0.0026 (6)	0.0022 (6)	−0.0110 (6)
C26	0.0225 (7)	0.0183 (7)	0.0163 (7)	−0.0051 (6)	0.0063 (6)	−0.0062 (6)
C28	0.0226 (8)	0.0214 (8)	0.0157 (7)	−0.0028 (6)	0.0021 (6)	−0.0049 (6)
C65	0.0180 (7)	0.0288 (8)	0.0166 (7)	−0.0040 (6)	−0.0012 (6)	−0.0080 (6)
C1	0.0149 (7)	0.0161 (7)	0.0169 (7)	−0.0025 (5)	0.0008 (5)	−0.0060 (6)
C23	0.0174 (7)	0.0181 (7)	0.0232 (8)	−0.0036 (6)	0.0070 (6)	−0.0078 (6)
C44	0.0205 (7)	0.0201 (7)	0.0157 (7)	−0.0075 (6)	−0.0003 (6)	−0.0039 (6)
C66	0.0212 (8)	0.0247 (8)	0.0167 (7)	−0.0085 (6)	0.0038 (6)	−0.0086 (6)
C12	0.0190 (7)	0.0202 (7)	0.0163 (7)	−0.0039 (6)	0.0012 (6)	−0.0049 (6)
C16	0.0202 (7)	0.0183 (7)	0.0205 (8)	−0.0032 (6)	0.0036 (6)	−0.0091 (6)
C62	0.0214 (8)	0.0250 (8)	0.0159 (7)	−0.0044 (6)	0.0001 (6)	−0.0061 (6)
C53	0.0215 (7)	0.0150 (7)	0.0209 (8)	−0.0016 (6)	0.0034 (6)	−0.0049 (6)
C38	0.0198 (7)	0.0207 (7)	0.0207 (7)	−0.0063 (6)	0.0028 (6)	−0.0110 (6)

C41	0.0202 (7)	0.0241 (8)	0.0174 (7)	−0.0080 (6)	0.0020 (6)	−0.0071 (6)
C31	0.0181 (7)	0.0219 (8)	0.0171 (7)	−0.0013 (6)	−0.0017 (6)	−0.0077 (6)
C64	0.0301 (9)	0.0235 (8)	0.0158 (7)	−0.0085 (7)	−0.0032 (6)	−0.0041 (6)
C45	0.0238 (8)	0.0165 (7)	0.0217 (8)	−0.0036 (6)	−0.0022 (6)	−0.0061 (6)
C9	0.0198 (7)	0.0202 (7)	0.0160 (7)	0.0002 (6)	0.0018 (6)	−0.0029 (6)
C40	0.0179 (7)	0.0306 (8)	0.0192 (8)	−0.0086 (6)	0.0059 (6)	−0.0116 (7)
C51	0.0201 (7)	0.0197 (7)	0.0204 (8)	−0.0027 (6)	−0.0009 (6)	−0.0097 (6)
C18	0.0234 (8)	0.0136 (7)	0.0219 (8)	−0.0016 (6)	−0.0003 (6)	−0.0051 (6)
C63	0.0214 (8)	0.0305 (9)	0.0201 (8)	0.0031 (7)	−0.0047 (6)	−0.0126 (7)
C6	0.0180 (7)	0.0259 (8)	0.0171 (7)	−0.0019 (6)	0.0000 (6)	−0.0072 (6)
C35	0.0172 (7)	0.0276 (8)	0.0187 (7)	−0.0024 (6)	0.0002 (6)	−0.0080 (6)
C25	0.0179 (7)	0.0270 (8)	0.0183 (7)	−0.0020 (6)	−0.0006 (6)	−0.0076 (6)
C58	0.0199 (7)	0.0210 (8)	0.0243 (8)	−0.0067 (6)	−0.0032 (6)	−0.0095 (6)
C11	0.0259 (8)	0.0206 (8)	0.0202 (8)	−0.0084 (6)	0.0024 (6)	−0.0062 (6)
C4	0.0211 (8)	0.0279 (8)	0.0238 (8)	−0.0087 (6)	0.0046 (6)	−0.0155 (7)
C30	0.0227 (8)	0.0296 (9)	0.0161 (7)	−0.0067 (7)	−0.0001 (6)	−0.0056 (7)
C46	0.0197 (7)	0.0210 (8)	0.0194 (7)	−0.0006 (6)	−0.0002 (6)	−0.0070 (6)
C39	0.0194 (7)	0.0252 (8)	0.0239 (8)	−0.0035 (6)	0.0023 (6)	−0.0146 (7)
C69	0.0247 (8)	0.0302 (9)	0.0223 (8)	−0.0134 (7)	0.0068 (6)	−0.0141 (7)
C67	0.0206 (8)	0.0287 (8)	0.0204 (8)	−0.0059 (6)	0.0004 (6)	−0.0118 (7)
C5	0.0165 (7)	0.0350 (9)	0.0196 (8)	−0.0055 (6)	0.0001 (6)	−0.0134 (7)
C10	0.0280 (8)	0.0156 (7)	0.0208 (8)	−0.0035 (6)	0.0041 (6)	−0.0044 (6)
C24	0.0229 (8)	0.0282 (8)	0.0181 (8)	−0.0039 (6)	0.0028 (6)	−0.0098 (7)
C33	0.0281 (8)	0.0228 (8)	0.0208 (8)	−0.0065 (7)	−0.0023 (6)	−0.0071 (7)
C34	0.0206 (8)	0.0314 (9)	0.0260 (8)	0.0049 (7)	−0.0017 (6)	−0.0146 (7)
C68	0.0267 (8)	0.0277 (9)	0.0221 (8)	−0.0006 (7)	0.0009 (6)	−0.0110 (7)
C60	0.0217 (8)	0.0293 (8)	0.0188 (8)	−0.0103 (7)	0.0047 (6)	−0.0091 (7)
C29	0.0305 (9)	0.0306 (9)	0.0203 (8)	−0.0173 (7)	0.0054 (7)	−0.0100 (7)
C59	0.0264 (8)	0.0330 (9)	0.0190 (8)	−0.0103 (7)	0.0024 (6)	−0.0121 (7)

*Geometric parameters (Å, º) for **6***

F1—C27	1.3363 (17)	C56—C55	1.394 (2)
F16—C70	1.3412 (18)	C56—C57	1.393 (2)
F8—C35	1.3398 (19)	C55—C36	1.5343 (19)
F2—C28	1.3391 (18)	C55—C60	1.397 (2)
F5—C32	1.3382 (18)	C50—C49	1.398 (2)
F12—C65	1.3392 (18)	C50—C51	1.385 (2)
F13—C67	1.3425 (18)	C2—C3	1.385 (2)
F15—C69	1.3461 (18)	C2—C1	1.5360 (19)
F9—C62	1.3346 (18)	C49—C54	1.396 (2)
F6—C33	1.3385 (19)	C49—C36	1.541 (2)
F4—C30	1.3336 (19)	C54—C53	1.386 (2)
F11—C64	1.3380 (19)	C14—C19	1.392 (2)
F14—C68	1.3412 (19)	C14—C1	1.541 (2)
F10—C63	1.3369 (19)	C22—C21	1.392 (2)
F7—C34	1.3427 (19)	C22—C23	1.377 (2)
F3—C29	1.3383 (19)	C57—C58	1.376 (2)
O3—C52	1.4133 (18)	C52—C53	1.380 (2)
O3—C61	1.3550 (18)	C52—C51	1.380 (2)
O1—C17	1.4089 (18)	C19—C18	1.388 (2)

O1—C26	1.3576 (18)	C17—C16	1.381 (2)
O2—C23	1.4185 (18)	C17—C18	1.382 (2)
O2—C31	1.3451 (19)	C61—C65	1.388 (2)
O4—C66	1.3445 (19)	C61—C62	1.385 (2)
O4—C58	1.4200 (18)	C47—C46	1.395 (2)
N1—C28	1.309 (2)	C32—C31	1.393 (2)
N1—C29	1.307 (2)	C32—C33	1.379 (2)
N3—C64	1.306 (2)	C27—C26	1.387 (2)
N3—C63	1.311 (2)	C27—C28	1.373 (2)
N4—C69	1.310 (2)	C70—C66	1.396 (2)
N4—C68	1.310 (2)	C70—C69	1.371 (2)
N2—C33	1.308 (2)	C3—C4	1.395 (2)
N2—C34	1.317 (2)	C26—C30	1.389 (2)
C43—C42	1.466 (2)	C65—C64	1.377 (2)
C43—C48	1.396 (2)	C23—C24	1.380 (2)
C43—C44	1.397 (2)	C44—C45	1.387 (2)
C42—C37	1.401 (2)	C66—C67	1.394 (2)
C42—C41	1.393 (2)	C12—C11	1.394 (2)
C20—C21	1.395 (2)	C62—C63	1.374 (2)
C20—C1	1.535 (2)	C38—C39	1.396 (2)
C20—C25	1.395 (2)	C41—C40	1.390 (2)
C13—C8	1.395 (2)	C31—C35	1.396 (2)
C13—C1	1.534 (2)	C45—C46	1.393 (2)
C13—C12	1.386 (2)	C9—C10	1.388 (2)
C15—C14	1.397 (2)	C40—C39	1.392 (2)
C15—C16	1.384 (2)	C6—C5	1.391 (2)
C48—C36	1.535 (2)	C35—C34	1.373 (2)
C48—C47	1.384 (2)	C25—C24	1.388 (2)
C7—C8	1.468 (2)	C58—C59	1.378 (2)
C7—C2	1.402 (2)	C11—C10	1.393 (2)
C7—C6	1.392 (2)	C4—C5	1.388 (2)
C37—C36	1.5358 (19)	C30—C29	1.374 (2)
C37—C38	1.384 (2)	C67—C68	1.380 (2)
C8—C9	1.395 (2)	C60—C59	1.389 (2)

C61—O3—C52	117.47 (12)	C27—C26—C30	116.77 (14)
C26—O1—C17	117.76 (11)	F2—C28—C27	118.32 (14)
C31—O2—C23	119.05 (12)	N1—C28—F2	117.07 (14)
C66—O4—C58	119.25 (12)	N1—C28—C27	124.59 (15)
C29—N1—C28	116.46 (14)	F12—C65—C61	120.91 (14)
C64—N3—C63	116.45 (14)	F12—C65—C64	120.30 (14)
C69—N4—C68	115.47 (14)	C64—C65—C61	118.78 (15)
C33—N2—C34	115.44 (14)	C20—C1—C2	113.46 (12)
C48—C43—C42	109.12 (13)	C20—C1—C14	113.87 (12)
C48—C43—C44	120.24 (14)	C13—C1—C20	109.60 (11)
C44—C43—C42	130.64 (14)	C13—C1—C2	100.87 (11)
C37—C42—C43	108.52 (13)	C13—C1—C14	112.39 (12)
C41—C42—C43	131.16 (14)	C2—C1—C14	105.92 (11)
C41—C42—C37	120.32 (14)	C22—C23—O2	118.92 (14)
C21—C20—C1	121.17 (13)	C22—C23—C24	122.32 (14)
C21—C20—C25	118.34 (14)	C24—C23—O2	118.73 (14)

C25—C20—C1	120.38 (13)	C45—C44—C43	118.63 (14)
C8—C13—C1	110.79 (13)	O4—C66—C70	116.13 (14)
C12—C13—C8	120.78 (14)	O4—C66—C67	128.28 (14)
C12—C13—C1	128.43 (13)	C67—C66—C70	115.58 (14)
C16—C15—C14	121.64 (14)	C13—C12—C11	118.64 (14)
C43—C48—C36	110.62 (13)	C17—C16—C15	119.13 (14)
C47—C48—C43	121.04 (14)	F9—C62—C61	120.59 (14)
C47—C48—C36	128.34 (13)	F9—C62—C63	120.73 (14)
C2—C7—C8	108.45 (13)	C63—C62—C61	118.68 (15)
C6—C7—C8	131.22 (14)	C52—C53—C54	119.04 (14)
C6—C7—C2	120.33 (14)	C37—C38—C39	118.38 (14)
C42—C37—C36	110.73 (13)	C40—C41—C42	118.53 (14)
C38—C37—C42	121.12 (14)	O2—C31—C32	128.34 (14)
C38—C37—C36	128.11 (13)	O2—C31—C35	116.06 (14)
C13—C8—C7	109.02 (13)	C32—C31—C35	115.60 (14)
C13—C8—C9	120.54 (14)	F11—C64—C65	118.39 (15)
C9—C8—C7	130.44 (14)	N3—C64—F11	117.10 (14)
C57—C56—C55	121.02 (14)	N3—C64—C65	124.50 (15)
C56—C55—C36	121.26 (13)	C44—C45—C46	120.96 (14)
C56—C55—C60	118.38 (14)	C10—C9—C8	118.59 (14)
C60—C55—C36	120.25 (13)	C41—C40—C39	120.94 (14)
C51—C50—C49	121.56 (14)	C52—C51—C50	118.87 (14)
C7—C2—C1	110.76 (13)	C17—C18—C19	118.97 (14)
C3—C2—C7	120.98 (14)	F10—C63—C62	117.90 (15)
C3—C2—C1	128.17 (14)	N3—C63—F10	117.34 (15)
C50—C49—C36	120.45 (13)	N3—C63—C62	124.75 (15)
C54—C49—C50	117.70 (13)	C5—C6—C7	118.52 (15)
C54—C49—C36	121.54 (13)	F8—C35—C31	119.51 (14)
C53—C54—C49	121.40 (14)	F8—C35—C34	121.04 (14)
C15—C14—C1	120.70 (13)	C34—C35—C31	119.45 (15)
C19—C14—C15	117.55 (13)	C24—C25—C20	121.36 (14)
C19—C14—C1	121.31 (13)	C57—C58—O4	118.74 (14)
C23—C22—C21	118.47 (14)	C57—C58—C59	122.61 (14)
C58—C57—C56	118.38 (14)	C59—C58—O4	118.61 (14)
C53—C52—O3	121.83 (14)	C10—C11—C12	120.66 (14)
C51—C52—O3	116.64 (13)	C5—C4—C3	120.82 (15)
C51—C52—C53	121.40 (14)	F4—C30—C26	120.33 (15)
C18—C19—C14	121.65 (14)	F4—C30—C29	121.10 (15)
C16—C17—O1	116.10 (13)	C29—C30—C26	118.57 (15)
C16—C17—C18	121.05 (14)	C45—C46—C47	120.46 (14)
C18—C17—O1	122.79 (13)	C40—C39—C38	120.70 (14)
C48—C36—C37	100.94 (11)	F15—C69—C70	118.49 (15)
C48—C36—C49	112.22 (12)	N4—C69—F15	116.43 (14)
C37—C36—C49	106.67 (11)	N4—C69—C70	125.08 (15)
C55—C36—C48	109.15 (12)	F13—C67—C66	122.49 (14)
C55—C36—C37	113.51 (12)	F13—C67—C68	118.73 (14)
C55—C36—C49	113.67 (12)	C68—C67—C66	118.78 (15)
C22—C21—C20	121.08 (14)	C4—C5—C6	120.97 (14)
O3—C61—C65	120.26 (14)	C9—C10—C11	120.76 (14)
O3—C61—C62	122.87 (14)	C23—C24—C25	118.29 (14)
C62—C61—C65	116.79 (14)	F6—C33—C32	117.57 (15)

C48—C47—C46	118.64 (14)	N2—C33—F6	117.02 (14)
F5—C32—C31	122.32 (14)	N2—C33—C32	125.42 (16)
F5—C32—C33	118.66 (14)	F7—C34—C35	118.59 (16)
C33—C32—C31	119.02 (15)	N2—C34—F7	116.44 (15)
F1—C27—C26	120.87 (13)	N2—C34—C35	124.97 (15)
F1—C27—C28	120.32 (14)	F14—C68—C67	117.70 (15)
C28—C27—C26	118.78 (14)	N4—C68—F14	116.81 (15)
F16—C70—C66	119.50 (14)	N4—C68—C67	125.49 (16)
F16—C70—C69	120.95 (14)	C59—C60—C55	121.35 (14)
C69—C70—C66	119.55 (15)	F3—C29—C30	118.38 (15)
C2—C3—C4	118.37 (14)	N1—C29—F3	116.84 (15)
O1—C26—C27	120.13 (14)	N1—C29—C30	124.77 (15)
O1—C26—C30	123.04 (14)	C58—C59—C60	118.13 (14)

7 (CCDC #: 2491516)

Computing details

Data collection: *CrysAlis PRO* system (CCD 43.144a 64-bit (release 23-01-2025)); cell refinement: *CrysAlis PRO* 1.171.43.144a (Rigaku OD, 2024); data reduction: *CrysAlis PRO* 1.171.43.144a (Rigaku OD, 2024); program(s) used to solve structure: SHELXT 2018/2 (Sheldrick, 2018); program(s) used to refine structure: *SHELXL* 2019/3 (Sheldrick, 2015); molecular graphics: Olex2 1.5 (Dolomanov *et al.*, 2009); software used to prepare material for publication: Olex2 1.5 (Dolomanov *et al.*, 2009).

Crystal data

$C_{22}H_8F_8N_2O_2$
M_r = 484.30
Monoclinic, $P2_1$
a = 9.70341 (5) Å
b = 8.40182 (4) Å
c = 11.37251 (6) Å
β = 95.0792 (5)°
V = 923.52 (1) Å^3
Z = 2
F(000) = 484
D_x = 1.742 Mg m^{-3}
Melting point: 479 K
Cu $K\alpha$ radiation, λ = 1.54184 Å
Cell parameters from 28896 reflections
θ = 3.9–68.7°
μ = 1.50 mm^{-1}
T = 100 K
Rhombohedral, clear colourless
0.32 × 0.21 × 0.17 mm

Data collection

XtaLAB Synergy, Dualflex, HyPix3000 diffractometer
Radiation source: micro-focus sealed X-ray tube, PhotonJet (Cu) X-ray Source
Mirror monochromator
Detector resolution: 10.0000 pixels mm^{-1}
ω scans
Absorption correction: gaussian *CrysAlis PRO* 1.171.43.144a (Rigaku Oxford Diffraction, 2024) Numerical absorption correction based on gaussian integration over a multifaceted crystal model Empirical absorption correction using spherical harmonics, implemented in SCALE3 ABSPACK scaling algorithm.
T_{min} = 0.532, T_{max} = 1.000
31755 measured reflections
3393 independent reflections
3379 reflections with $I > 2\sigma(I)$
R_{int} = 0.029
θ_{max} = 68.8°, θ_{min} = 3.9°
h = −11→11
k = −10→10
l = −13→13

Refinement

Refinement on F^2
Least-squares matrix: full
$R[F^2 > 2\sigma(F^2)]$ = 0.024
$wR(F^2)$ = 0.069
S = 1.05
3393 reflections
308 parameters
1 restraint
Primary atom site location: dual
Hydrogen site location: inferred from neighbouring sites
H-atom parameters constrained
$w = 1/[\sigma^2(F_o^2) + (0.0414P)^2 + 0.2273P]$ where $P = (F_o^2 + 2F_c^2)/3$
$(\Delta/\sigma)_{max}$ < 0.001
$\Delta\rho_{max}$ = 0.22 e Å^{-3}
$\Delta\rho_{min}$ = −0.17 e Å^{-3}
Extinction correction: *SHELXL2019*/2 (Sheldrick 2019), $Fc^* = kFc[1+0.001xFc^2\lambda^3/\sin(2\theta)]^{-1/4}$

Extinction coefficient: 0.0052 (5)

Absolute structure: Flack x determined using 1536 quotients [(I+)-(I-)]/[(I+)+(I-)] (Parsons, Flack and Wagner, Acta Cryst. B69 (2013) 249-259).
Absolute structure parameter: 0.02 (2)

Special details

Geometry. All esds (except the esd in the dihedral angle between two l.s. planes) are estimated using the full covariance matrix. The cell esds are taken into account individually in the estimation of esds in distances, angles and torsion angles; correlations between esds in cell parameters are only used when they are defined by crystal symmetry. An approximate (isotropic) treatment of cell esds is used for estimating esds involving l.s. planes.

Fractional atomic coordinates and isotropic or equivalent isotropic displacement parameters (Å^2) for **7**

	x	y	z	U_{iso}*/U_{eq}
F1	1.12904 (13)	0.76420 (19)	1.35370 (12)	0.0326 (4)
F2	1.00117 (14)	0.89697 (18)	1.15541 (11)	0.0273 (3)
F3	0.62027 (12)	0.56184 (17)	1.20613 (11)	0.0270 (3)
F4	0.76984 (16)	0.43474 (18)	1.39504 (12)	0.0343 (4)
F5	−0.13648 (13)	0.2325 (2)	0.15169 (11)	0.0317 (3)
F6	0.00888 (13)	0.10396 (18)	0.34399 (11)	0.0267 (3)
F7	0.35852 (13)	0.48259 (17)	0.29124 (11)	0.0274 (3)
F8	0.19672 (15)	0.59389 (18)	0.10363 (12)	0.0332 (3)
O1	0.73931 (15)	0.80845 (19)	1.08002 (12)	0.0216 (3)
O2	0.26550 (15)	0.2137 (2)	0.41345 (12)	0.0213 (3)
N1	0.9483 (2)	0.6012 (2)	1.37433 (16)	0.0241 (4)
N2	0.0322 (2)	0.4105 (2)	0.12762 (16)	0.0232 (4)
C1	1.0014 (2)	0.7165 (3)	1.31489 (19)	0.0219 (5)
C2	0.9377 (2)	0.7853 (3)	1.21452 (18)	0.0198 (5)
C3	0.8057 (2)	0.7341 (3)	1.17415 (17)	0.0175 (4)
C4	0.7479 (2)	0.6147 (3)	1.23808 (18)	0.0203 (5)
C5	0.8243 (2)	0.5528 (3)	1.33579 (19)	0.0229 (5)
C11	0.7247 (2)	0.5725 (3)	0.95455 (19)	0.0206 (4)
H11	0.806833	0.529896	0.994076	0.025*
C10	0.6555 (2)	0.4924 (3)	0.86019 (18)	0.0198 (4)
H10	0.690093	0.392654	0.836721	0.024*
C9	0.5363 (2)	0.5546 (3)	0.79870 (18)	0.0169 (4)
C8	0.4882 (2)	0.7010 (3)	0.83648 (18)	0.0220 (4)
H8	0.408109	0.746668	0.795711	0.026*
C7	0.5543 (2)	0.7815 (3)	0.93189 (19)	0.0224 (4)
H7	0.519468	0.880325	0.956951	0.027*
C6	0.6713 (2)	0.7155 (3)	0.98968 (17)	0.0180 (4)
C13	0.3311 (2)	0.5114 (3)	0.64973 (17)	0.0183 (4)
H13	0.284856	0.596145	0.685186	0.022*
C12	0.4647 (2)	0.4694 (3)	0.69568 (18)	0.0166 (4)
C17	0.5291 (2)	0.3446 (3)	0.64087 (19)	0.0246 (5)
H17	0.620363	0.314589	0.669435	0.030*
C16	0.4631 (2)	0.2631 (3)	0.54566 (19)	0.0255 (5)
H16	0.508043	0.177460	0.510112	0.031*
C15	0.3319 (2)	0.3084 (3)	0.50372 (17)	0.0188 (4)
C14	0.2642 (2)	0.4321 (3)	0.55319 (18)	0.0184 (4)
H14	0.173964	0.462829	0.522262	0.022*
C18	−0.0111 (2)	0.2901 (3)	0.18726 (19)	0.0220 (5)

C19	0.0617 (2)	0.2237 (3)	0.28493 (17)	0.0195 (4)
C20	0.1902 (2)	0.2865 (3)	0.32287 (18)	0.0184 (4)
C21	0.2366 (2)	0.4145 (3)	0.26010 (18)	0.0202 (4)
C22	0.1531 (2)	0.4698 (3)	0.16427 (19)	0.0224 (5)

Atomic displacement parameters (Å²) for ***7***

	U^{11}	U^{22}	U^{33}	U^{12}	U^{13}	U^{23}
F1	0.0179 (6)	0.0470 (10)	0.0311 (7)	−0.0012 (6)	−0.0079 (5)	−0.0066 (6)
F2	0.0266 (7)	0.0315 (7)	0.0239 (6)	−0.0113 (6)	0.0022 (5)	−0.0007 (6)
F3	0.0216 (6)	0.0324 (7)	0.0264 (6)	−0.0106 (5)	−0.0002 (5)	−0.0027 (5)
F4	0.0502 (9)	0.0281 (7)	0.0248 (7)	−0.0080 (6)	0.0043 (6)	0.0067 (5)
F5	0.0186 (6)	0.0479 (9)	0.0270 (6)	−0.0053 (6)	−0.0066 (5)	−0.0031 (6)
F6	0.0293 (7)	0.0291 (7)	0.0212 (7)	−0.0118 (6)	−0.0002 (5)	0.0014 (5)
F7	0.0223 (6)	0.0323 (7)	0.0273 (6)	−0.0114 (6)	0.0002 (5)	−0.0031 (6)
F8	0.0453 (8)	0.0290 (7)	0.0256 (7)	−0.0059 (7)	0.0039 (6)	0.0072 (6)
O1	0.0260 (8)	0.0194 (8)	0.0178 (7)	−0.0014 (6)	−0.0076 (6)	−0.0012 (6)
O2	0.0236 (7)	0.0203 (8)	0.0180 (7)	0.0013 (6)	−0.0081 (6)	−0.0031 (6)
N1	0.0267 (10)	0.0282 (11)	0.0167 (8)	0.0054 (9)	−0.0020 (7)	−0.0024 (8)
N2	0.0238 (9)	0.0284 (11)	0.0167 (8)	0.0044 (8)	−0.0011 (7)	−0.0025 (8)
C1	0.0163 (9)	0.0282 (12)	0.0204 (9)	0.0018 (9)	−0.0028 (8)	−0.0064 (10)
C2	0.0192 (10)	0.0217 (11)	0.0187 (9)	−0.0036 (8)	0.0032 (8)	−0.0037 (9)
C3	0.0192 (9)	0.0194 (10)	0.0135 (9)	0.0008 (9)	−0.0014 (7)	−0.0045 (8)
C4	0.0190 (10)	0.0237 (11)	0.0181 (10)	−0.0028 (8)	0.0012 (8)	−0.0060 (9)
C5	0.0293 (11)	0.0213 (11)	0.0186 (10)	−0.0004 (9)	0.0040 (8)	−0.0018 (9)
C11	0.0184 (10)	0.0236 (11)	0.0190 (10)	0.0039 (9)	−0.0034 (8)	0.0012 (9)
C10	0.0201 (10)	0.0200 (11)	0.0188 (9)	0.0032 (8)	−0.0009 (8)	−0.0030 (9)
C9	0.0140 (9)	0.0200 (10)	0.0162 (9)	−0.0008 (8)	−0.0003 (7)	0.0012 (8)
C8	0.0168 (9)	0.0236 (11)	0.0242 (10)	0.0045 (8)	−0.0053 (8)	−0.0024 (9)
C7	0.0218 (10)	0.0207 (10)	0.0239 (10)	0.0026 (9)	−0.0024 (8)	−0.0033 (9)
C6	0.0190 (9)	0.0206 (10)	0.0136 (8)	−0.0032 (8)	−0.0020 (7)	−0.0013 (8)
C13	0.0173 (10)	0.0197 (11)	0.0177 (9)	0.0025 (8)	0.0004 (7)	−0.0003 (8)
C12	0.0157 (9)	0.0190 (11)	0.0151 (9)	0.0000 (8)	0.0002 (7)	0.0012 (8)
C17	0.0175 (10)	0.0312 (13)	0.0239 (10)	0.0070 (9)	−0.0050 (8)	−0.0068 (9)
C16	0.0230 (11)	0.0277 (13)	0.0246 (10)	0.0079 (9)	−0.0039 (8)	−0.0099 (9)
C15	0.0197 (10)	0.0208 (11)	0.0151 (9)	−0.0025 (8)	−0.0026 (8)	−0.0014 (8)
C14	0.0141 (9)	0.0225 (11)	0.0176 (9)	0.0014 (8)	−0.0033 (7)	0.0007 (8)
C18	0.0160 (10)	0.0304 (12)	0.0191 (10)	−0.0014 (9)	−0.0019 (8)	−0.0074 (10)
C19	0.0198 (10)	0.0227 (11)	0.0161 (9)	−0.0024 (9)	0.0015 (8)	−0.0020 (9)
C20	0.0179 (9)	0.0214 (11)	0.0155 (9)	0.0027 (9)	−0.0012 (8)	−0.0039 (9)
C21	0.0178 (10)	0.0219 (11)	0.0209 (10)	−0.0029 (8)	0.0008 (8)	−0.0049 (9)
C22	0.0289 (11)	0.0201 (11)	0.0187 (10)	−0.0002 (9)	0.0053 (8)	0.0001 (8)

Geometric parameters (Å, º) for ***7***

F1—C1	1.339 (2)	C10—H10	0.9500
F2—C2	1.336 (3)	C10—C9	1.399 (3)
F3—C4	1.336 (2)	C9—C8	1.397 (3)
F4—C5	1.334 (3)	C9—C12	1.491 (2)
F5—C18	1.339 (2)	C8—H8	0.9500
F6—C19	1.337 (3)	C8—C7	1.386 (3)

F7—C21	1.334 (2)	C7—H7	0.9500
F8—C22	1.340 (3)	C7—C6	1.377 (3)
O1—C3	1.353 (3)	C13—H13	0.9500
O1—C6	1.408 (2)	C13—C12	1.399 (3)
O2—C15	1.409 (2)	C13—C14	1.394 (3)
O2—C20	1.355 (2)	C12—C17	1.395 (3)
N1—C1	1.312 (3)	C17—H17	0.9500
N1—C5	1.309 (3)	C17—C16	1.388 (3)
N2—C18	1.307 (3)	C16—H16	0.9500
N2—C22	1.308 (3)	C16—C15	1.373 (3)
C1—C2	1.376 (3)	C15—C14	1.377 (3)
C2—C3	1.390 (3)	C14—H14	0.9500
C3—C4	1.386 (3)	C18—C19	1.380 (3)
C4—C5	1.382 (3)	C19—C20	1.387 (3)
C11—H11	0.9500	C20—C21	1.388 (3)
C11—C10	1.388 (3)	C21—C22	1.380 (3)
C11—C6	1.381 (3)		

C3—O1—C6	118.75 (17)	C7—C6—O1	116.0 (2)
C20—O2—C15	118.70 (17)	C7—C6—C11	121.8 (2)
C5—N1—C1	116.63 (19)	C12—C13—H13	119.2
C18—N2—C22	116.69 (19)	C14—C13—H13	119.2
F1—C1—C2	118.8 (2)	C14—C13—C12	121.6 (2)
N1—C1—F1	116.43 (19)	C13—C12—C9	121.59 (17)
N1—C1—C2	124.70 (19)	C17—C12—C9	121.05 (17)
F2—C2—C1	121.27 (18)	C17—C12—C13	117.36 (19)
F2—C2—C3	120.24 (19)	C12—C17—H17	119.2
C1—C2—C3	118.5 (2)	C16—C17—C12	121.7 (2)
O1—C3—C2	118.6 (2)	C16—C17—H17	119.2
O1—C3—C4	124.25 (19)	C17—C16—H16	120.5
C4—C3—C2	117.05 (19)	C15—C16—C17	118.9 (2)
F3—C4—C3	120.45 (19)	C15—C16—H16	120.5
F3—C4—C5	120.8 (2)	C16—C15—O2	116.56 (19)
C5—C4—C3	118.75 (19)	C16—C15—C14	121.92 (19)
F4—C5—C4	118.55 (19)	C14—C15—O2	121.33 (18)
N1—C5—F4	117.10 (19)	C13—C14—H14	120.8
N1—C5—C4	124.3 (2)	C15—C14—C13	118.48 (19)
C10—C11—H11	120.7	C15—C14—H14	120.8
C6—C11—H11	120.7	F5—C18—C19	118.9 (2)
C6—C11—C10	118.51 (19)	N2—C18—F5	116.6 (2)
C11—C10—H10	119.1	N2—C18—C19	124.4 (2)
C11—C10—C9	121.8 (2)	F6—C19—C18	121.12 (18)
C9—C10—H10	119.1	F6—C19—C20	120.27 (18)
C10—C9—C12	121.38 (17)	C18—C19—C20	118.6 (2)
C8—C9—C10	117.32 (18)	O2—C20—C19	118.3 (2)
C8—C9—C12	121.29 (16)	O2—C20—C21	124.29 (19)
C9—C8—H8	119.1	C19—C20—C21	117.27 (19)
C7—C8—C9	121.75 (19)	F7—C21—C20	121.09 (18)
C7—C8—H8	119.1	F7—C21—C22	120.7 (2)
C8—C7—H7	120.6	C22—C21—C20	118.21 (19)
C6—C7—C8	118.8 (2)	F8—C22—C21	118.6 (2)

C6—C7—H7	120.6	N2—C22—F8	116.61 (19)
C11—C6—O1	122.05 (19)	N2—C22—C21	124.8 (2)

F1—C1—C2—F2	0.5 (3)	C10—C11—C6—C7	−1.6 (3)
F1—C1—C2—C3	179.40 (19)	C10—C9—C8—C7	−0.8 (3)
F2—C2—C3—O1	−5.3 (3)	C10—C9—C12—C13	166.1 (2)
F2—C2—C3—C4	178.21 (19)	C10—C9—C12—C17	−14.2 (3)
F3—C4—C5—F4	2.5 (3)	C9—C8—C7—C6	0.8 (3)
F3—C4—C5—N1	−178.16 (19)	C9—C12—C17—C16	179.3 (2)
F5—C18—C19—F6	−0.2 (3)	C8—C9—C12—C13	−15.0 (3)
F5—C18—C19—C20	−178.55 (19)	C8—C9—C12—C17	164.7 (2)
F6—C19—C20—O2	6.3 (3)	C8—C7—C6—O1	175.43 (19)
F6—C19—C20—C21	−177.66 (19)	C8—C7—C6—C11	0.5 (3)
F7—C21—C22—F8	−0.4 (3)	C6—O1—C3—C2	134.7 (2)
F7—C21—C22—N2	−179.90 (19)	C6—O1—C3—C4	−49.1 (3)
O1—C3—C4—F3	2.3 (3)	C6—C11—C10—C9	1.6 (3)
O1—C3—C4—C5	−177.18 (19)	C13—C12—C17—C16	−1.0 (3)
O2—C15—C14—C13	173.91 (19)	C12—C9—C8—C7	−179.77 (19)
O2—C20—C21—F7	−4.7 (3)	C12—C13—C14—C15	0.8 (3)
O2—C20—C21—C22	175.3 (2)	C12—C17—C16—C15	0.9 (4)
N1—C1—C2—F2	−176.7 (2)	C17—C16—C15—O2	−175.0 (2)
N1—C1—C2—C3	2.2 (3)	C17—C16—C15—C14	0.1 (3)
N2—C18—C19—F6	177.7 (2)	C16—C15—C14—C13	−0.9 (3)
N2—C18—C19—C20	−0.7 (3)	C15—O2—C20—C19	−135.9 (2)
C1—N1—C5—F4	179.45 (19)	C15—O2—C20—C21	48.4 (3)
C1—N1—C5—C4	0.1 (3)	C14—C13—C12—C9	179.82 (18)
C1—C2—C3—O1	175.82 (19)	C14—C13—C12—C17	0.1 (3)
C1—C2—C3—C4	−0.7 (3)	C18—N2—C22—F8	−179.47 (19)
C2—C3—C4—F3	178.54 (19)	C18—N2—C22—C21	0.0 (3)
C2—C3—C4—C5	−0.9 (3)	C18—C19—C20—O2	−175.32 (19)
C3—O1—C6—C11	−37.1 (3)	C18—C19—C20—C21	0.7 (3)
C3—O1—C6—C7	147.93 (19)	C19—C20—C21—F7	179.51 (19)
C3—C4—C5—F4	−178.06 (19)	C19—C20—C21—C22	−0.5 (3)
C3—C4—C5—N1	1.3 (3)	C20—O2—C15—C16	−139.4 (2)
C5—N1—C1—F1	−179.15 (19)	C20—O2—C15—C14	45.5 (3)
C5—N1—C1—C2	−1.9 (3)	C20—C21—C22—F8	179.60 (18)
C11—C10—C9—C8	−0.4 (3)	C20—C21—C22—N2	0.1 (3)
C11—C10—C9—C12	178.55 (18)	C22—N2—C18—F5	178.19 (19)
C10—C11—C6—O1	−176.30 (19)	C22—N2—C18—C19	0.3 (3)

8 (CCDC #: 2483195)

Computing details

Data collection: *CrysAlis PRO* system (CCD 43.144a 64-bit (release 23-01-2025)); cell refinement: *CrysAlis PRO* 1.171.43.90 (Rigaku OD, 2023); data reduction: *CrysAlis PRO* 1.171.43.90 (Rigaku OD, 2023); program(s) used to solve structure: SHELXT 2018/2 (Sheldrick, 2018); program(s) used to refine structure: *SHELXL* 2019/3 (Sheldrick, 2015); molecular graphics: Olex2 1.5 (Dolomanov *et al.*, 2009); software used to prepare material for publication: Olex2 1.5 (Dolomanov *et al.*, 2009).

Crystal data

$C_{34}H_{24}F_8N_2O_2$
M_r = 644.55
Monoclinic, $P2_1/c$
a = 15.1049 (3) Å
b = 9.1126 (2) Å
c = 10.2335 (2) Å
β = 97.887 (2)°
V = 1395.26 (5) Å^3
Z = 2
F(000) = 660
D_x = 1.534 Mg m^{-3}
Melting point: 460 K
Cu Kα radiation, λ = 1.54184 Å
Cell parameters from 8854 reflections
θ = 2.9–68.7°
μ = 1.15 mm^{-1}
T = 100 K
Plate, clear colourless
0.58 × 0.27 × 0.05 mm

Data collection

XtaLAB Synergy, Dualflex, HyPix3000 diffractometer
Radiation source: micro-focus sealed X-ray tube, PhotonJet (Cu) X-ray Source
Mirror monochromator
Detector resolution: 10.0000 pixels mm^{-1}
ω scans
Absorption correction: gaussian *CrysAlis PRO* 1.171.43.90 (Rigaku Oxford Diffraction, 2023) Numerical absorption correction based on gaussian integration over a multifaceted crystal model Empirical absorption correction using spherical harmonics, implemented in SCALE3 ABSPACK scaling algorithm.
T_{min} = 0.428, T_{max} = 1.000
13832 measured reflections
2569 independent reflections
2212 reflections with $I > 2\sigma(I)$
R_{int} = 0.038
θ_{max} = 68.7°, θ_{min} = 3.0°
h = −18→18
k = −10→8
l = −12→12

Refinement

Refinement on F^2
Least-squares matrix: full
$R[F^2 > 2\sigma(F^2)]$ = 0.038
$wR(F^2)$ = 0.105
S = 1.04
2569 reflections
210 parameters
0 restraints
Primary atom site location: dual
Hydrogen site location: inferred from neighbouring sites
H-atom parameters constrained
$w = 1/[\sigma^2(F_o^2) + (0.0539P)^2 + 0.6336P]$ where $P = (F_o^2 + 2F_c^2)/3$
$(\Delta/\sigma)_{max}$ = 0.001
$\Delta\rho_{max}$ = 0.28 e Å^{-3}
$\Delta\rho_{min}$ = −0.21 e Å^{-3}

Special details

Geometry. All esds (except the esd in the dihedral angle between two l.s. planes) are estimated using the full covariance matrix. The cell esds are taken into account individually in the estimation of esds in distances, angles and torsion angles; correlations between esds in cell parameters are only used when they are defined by crystal symmetry. An approximate (isotropic) treatment of cell esds is used for estimating esds involving l.s. planes.

*Fractional atomic coordinates and isotropic or equivalent isotropic displacement parameters (Å²) for **8***

	x	*y*	*z*	U_{iso}*/U_{eq}
C1	0.93304 (10)	1.09885 (16)	0.45500 (15)	0.0215 (3)
H1	0.887434	1.167852	0.425835	0.026*
F1	0.65625 (7)	0.22059 (11)	0.53341 (10)	0.0370 (3)
N1	0.44488 (9)	0.37357 (15)	0.39716 (14)	0.0300 (3)
O1	0.71177 (7)	0.39220 (12)	0.34314 (11)	0.0267 (3)
C2	0.94188 (10)	0.97275 (16)	0.38090 (14)	0.0201 (3)
F2	0.48042 (7)	0.21379 (12)	0.56293 (10)	0.0400 (3)
C3	1.00977 (10)	0.87516 (16)	0.42878 (15)	0.0216 (3)
H3	1.017202	0.788056	0.380442	0.026*
F3	0.40770 (7)	0.53325 (11)	0.23057 (11)	0.0390 (3)
C4	0.81130 (11)	1.05686 (17)	0.21225 (16)	0.0255 (3)
H4A	0.774176	1.068292	0.282963	0.038*
H4B	0.773574	1.028337	0.130563	0.038*
H4C	0.841104	1.150053	0.198645	0.038*
F4	0.57744 (6)	0.55174 (10)	0.18078 (10)	0.0330 (3)
C5	0.88199 (10)	0.93746 (16)	0.25081 (15)	0.0214 (3)
C6	0.94074 (11)	0.92901 (18)	0.14000 (15)	0.0259 (3)
H6A	0.969666	1.024096	0.131248	0.039*
H6B	0.903567	0.904284	0.056758	0.039*
H6C	0.986508	0.853255	0.161224	0.039*
C7	0.83474 (10)	0.79114 (16)	0.27019 (14)	0.0204 (3)
C8	0.85113 (10)	0.66300 (17)	0.20342 (15)	0.0238 (3)
H8	0.892034	0.665076	0.140724	0.029*
C9	0.80862 (10)	0.53163 (17)	0.22688 (16)	0.0244 (3)
H9	0.820254	0.444900	0.180619	0.029*
C10	0.74984 (10)	0.52951 (16)	0.31761 (15)	0.0234 (3)
C11	0.73177 (10)	0.65372 (17)	0.38724 (15)	0.0234 (3)
H11	0.691261	0.650219	0.450454	0.028*
C12	0.77443 (10)	0.78338 (16)	0.36212 (15)	0.0226 (3)
H12	0.762318	0.869563	0.408794	0.027*
C13	0.62428 (10)	0.39028 (17)	0.35856 (16)	0.0245 (3)
C14	0.59665 (11)	0.30033 (17)	0.45489 (16)	0.0278 (4)
C15	0.50723 (12)	0.29728 (18)	0.46859 (16)	0.0297 (4)
C16	0.47111 (11)	0.45587 (18)	0.30498 (17)	0.0289 (4)
C17	0.55796 (11)	0.46834 (17)	0.28007 (16)	0.0263 (4)

*Atomic displacement parameters (Å²) for **8***

	U^{11}	U^{22}	U^{33}	U^{12}	U^{13}	U^{23}
C1	0.0213 (7)	0.0151 (7)	0.0285 (8)	0.0008 (6)	0.0052 (6)	0.0013 (6)
F1	0.0394 (6)	0.0311 (6)	0.0383 (6)	−0.0035 (4)	−0.0028 (4)	0.0117 (4)
N1	0.0304 (7)	0.0247 (7)	0.0352 (8)	−0.0056 (6)	0.0059 (6)	−0.0035 (6)

O1	0.0258 (6)	0.0170 (5)	0.0376 (6)	−0.0023 (4)	0.0053 (5)	0.0031 (4)
C2	0.0199 (7)	0.0172 (7)	0.0235 (7)	−0.0040 (6)	0.0046 (6)	0.0009 (6)
F2	0.0458 (6)	0.0389 (6)	0.0369 (6)	−0.0104 (5)	0.0119 (5)	0.0080 (4)
C3	0.0234 (7)	0.0155 (7)	0.0263 (8)	−0.0014 (6)	0.0056 (6)	−0.0023 (6)
F3	0.0290 (5)	0.0344 (6)	0.0521 (7)	0.0011 (4)	0.0000 (5)	0.0082 (5)
C4	0.0287 (8)	0.0186 (8)	0.0279 (8)	0.0000 (6)	−0.0009 (6)	0.0005 (6)
F4	0.0329 (5)	0.0292 (5)	0.0365 (5)	−0.0034 (4)	0.0032 (4)	0.0107 (4)
C5	0.0233 (7)	0.0163 (7)	0.0243 (7)	−0.0014 (6)	0.0019 (6)	0.0002 (6)
C6	0.0306 (8)	0.0207 (7)	0.0267 (8)	−0.0042 (6)	0.0052 (6)	−0.0007 (6)
C7	0.0197 (7)	0.0179 (7)	0.0224 (7)	−0.0009 (6)	−0.0013 (6)	0.0008 (6)
C8	0.0241 (8)	0.0206 (8)	0.0268 (8)	−0.0002 (6)	0.0036 (6)	−0.0008 (6)
C9	0.0253 (8)	0.0177 (8)	0.0299 (8)	0.0003 (6)	0.0029 (6)	−0.0030 (6)
C10	0.0225 (7)	0.0177 (8)	0.0291 (8)	−0.0026 (6)	−0.0001 (6)	0.0022 (6)
C11	0.0221 (7)	0.0242 (8)	0.0242 (7)	−0.0013 (6)	0.0035 (6)	0.0002 (6)
C12	0.0238 (7)	0.0187 (8)	0.0247 (7)	0.0001 (6)	0.0008 (6)	−0.0026 (6)
C13	0.0269 (8)	0.0176 (7)	0.0289 (8)	−0.0043 (6)	0.0035 (6)	−0.0026 (6)
C14	0.0330 (9)	0.0205 (8)	0.0286 (8)	−0.0044 (7)	−0.0003 (7)	0.0006 (6)
C15	0.0373 (9)	0.0236 (8)	0.0289 (8)	−0.0093 (7)	0.0074 (7)	−0.0007 (6)
C16	0.0287 (8)	0.0230 (8)	0.0340 (9)	−0.0020 (7)	0.0004 (7)	−0.0011 (7)
C17	0.0310 (8)	0.0185 (8)	0.0290 (8)	−0.0043 (6)	0.0028 (7)	0.0011 (6)

*Geometric parameters (Å, º) for **8***

C1—H1	0.9500	C5—C7	1.538 (2)
C1—C2	1.393 (2)	C6—H6A	0.9800
C1—C3^{i}	1.391 (2)	C6—H6B	0.9800
F1—C14	1.3363 (19)	C6—H6C	0.9800
N1—C15	1.310 (2)	C7—C8	1.392 (2)
N1—C16	1.308 (2)	C7—C12	1.398 (2)
O1—C10	1.4163 (18)	C8—H8	0.9500
O1—C13	1.3523 (19)	C8—C9	1.395 (2)
C2—C3	1.394 (2)	C9—H9	0.9500
C2—C5	1.537 (2)	C9—C10	1.370 (2)
F2—C15	1.3349 (19)	C10—C11	1.384 (2)
C3—H3	0.9500	C11—H11	0.9500
F3—C16	1.3396 (19)	C11—C12	1.387 (2)
C4—H4A	0.9800	C12—H12	0.9500
C4—H4B	0.9800	C13—C14	1.390 (2)
C4—H4C	0.9800	C13—C17	1.391 (2)
C4—C5	1.538 (2)	C14—C15	1.377 (2)
F4—C17	1.3337 (18)	C16—C17	1.375 (2)
C5—C6	1.535 (2)		
C2—C1—H1	119.6	C12—C7—C5	119.17 (13)
C3^{i}—C1—H1	119.6	C7—C8—H8	119.4
C3^{i}—C1—C2	120.74 (14)	C7—C8—C9	121.22 (14)
C16—N1—C15	116.17 (15)	C9—C8—H8	119.4
C13—O1—C10	117.45 (12)	C8—C9—H9	120.5
C1—C2—C3	117.10 (14)	C10—C9—C8	119.03 (14)
C1—C2—C5	123.70 (13)	C10—C9—H9	120.5
C3—C2—C5	119.20 (13)	C9—C10—O1	117.06 (13)

C1^{i}—C3—C2	122.16 (14)	C9—C10—C11	121.95 (14)
C1^{i}—C3—H3	118.9	C11—C10—O1	120.90 (14)
C2—C3—H3	118.9	C10—C11—H11	120.9
H4A—C4—H4B	109.5	C10—C11—C12	118.14 (14)
H4A—C4—H4C	109.5	C12—C11—H11	120.9
H4B—C4—H4C	109.5	C7—C12—H12	119.0
C5—C4—H4A	109.5	C11—C12—C7	122.01 (14)
C5—C4—H4B	109.5	C11—C12—H12	119.0
C5—C4—H4C	109.5	O1—C13—C14	118.94 (14)
C2—C5—C4	112.31 (13)	O1—C13—C17	124.33 (14)
C2—C5—C7	107.34 (12)	C14—C13—C17	116.67 (15)
C4—C5—C7	109.17 (12)	F1—C14—C13	120.28 (15)
C6—C5—C2	108.61 (12)	F1—C14—C15	121.13 (14)
C6—C5—C4	107.30 (12)	C15—C14—C13	118.58 (15)
C6—C5—C7	112.17 (12)	N1—C15—F2	116.10 (15)
C5—C6—H6A	109.5	N1—C15—C14	124.93 (15)
C5—C6—H6B	109.5	F2—C15—C14	118.95 (15)
C5—C6—H6C	109.5	N1—C16—F3	116.61 (15)
H6A—C6—H6B	109.5	N1—C16—C17	124.89 (15)
H6A—C6—H6C	109.5	F3—C16—C17	118.49 (15)
H6B—C6—H6C	109.5	F4—C17—C13	120.98 (14)
C8—C7—C5	123.16 (13)	F4—C17—C16	120.28 (15)
C8—C7—C12	117.64 (14)	C16—C17—C13	118.73 (15)

C1—C2—C3—C1^{i}	−0.2 (2)	C5—C7—C8—C9	178.13 (14)
C1—C2—C5—C4	−0.1 (2)	C5—C7—C12—C11	−177.95 (13)
C1—C2—C5—C6	118.39 (15)	C6—C5—C7—C8	6.1 (2)
C1—C2—C5—C7	−120.13 (15)	C6—C5—C7—C12	−176.13 (13)
F1—C14—C15—N1	178.94 (15)	C7—C8—C9—C10	−0.1 (2)
F1—C14—C15—F2	0.6 (2)	C8—C7—C12—C11	0.0 (2)
N1—C16—C17—F4	177.82 (14)	C8—C9—C10—O1	−176.84 (13)
N1—C16—C17—C13	−0.9 (3)	C8—C9—C10—C11	−0.4 (2)
O1—C10—C11—C12	176.96 (13)	C9—C10—C11—C12	0.6 (2)
O1—C13—C14—F1	2.0 (2)	C10—O1—C13—C14	−140.36 (14)
O1—C13—C14—C15	−179.00 (14)	C10—O1—C13—C17	42.5 (2)
O1—C13—C17—F4	0.6 (2)	C10—C11—C12—C7	−0.4 (2)
O1—C13—C17—C16	179.30 (14)	C12—C7—C8—C9	0.3 (2)
C2—C5—C7—C8	−113.16 (16)	C13—O1—C10—C9	−139.85 (15)
C2—C5—C7—C12	64.64 (17)	C13—O1—C10—C11	43.6 (2)
C3^{i}—C1—C2—C3	0.2 (2)	C13—C14—C15—N1	−0.1 (3)
C3^{i}—C1—C2—C5	−179.25 (13)	C13—C14—C15—F2	−178.45 (14)
C3—C2—C5—C4	−179.61 (13)	C14—C13—C17—F4	−176.61 (14)
C3—C2—C5—C6	−61.10 (17)	C14—C13—C17—C16	2.1 (2)
C3—C2—C5—C7	60.39 (17)	C15—N1—C16—F3	179.38 (14)
F3—C16—C17—F4	−2.4 (2)	C15—N1—C16—C17	−0.8 (2)
F3—C16—C17—C13	178.86 (14)	C16—N1—C15—F2	179.76 (14)
C4—C5—C7—C8	124.86 (15)	C16—N1—C15—C14	1.3 (2)
C4—C5—C7—C12	−57.33 (18)	C17—C13—C14—F1	179.31 (14)
C5—C2—C3—C1^{i}	179.27 (13)	C17—C13—C14—C15	−1.7 (2)

9 (CCDC: #2504217)

Computing details

Data collection: *CrysAlis PRO* system (CCD 43.144a 64-bit (release 23-01-2025)); cell refinement: *CrysAlis PRO* 1.171.43.90 (Rigaku OD, 2023); data reduction: *CrysAlis PRO* 1.171.43.90 (Rigaku OD, 2023); program(s) used to solve structure: SHELXT 2018/2 (Sheldrick, 2018); program(s) used to refine structure: *SHELXL* 2019/3 (Sheldrick, 2015); molecular graphics: Olex2 1.5 (Dolomanov *et al.*, 2009); software used to prepare material for publication: Olex2 1.5 (Dolomanov *et al.*, 2009).

Crystal data

$C_{35}H_{18}F_8N_2O_2$
M_r = 650.51
Tetragonal, $I4_1/a$
a = 39.1978 (6) Å
c = 7.4423 (2) Å
V = 11434.9 (5) Å^3
Z = 16
F(000) = 5280
D_x = 1.511 Mg m^{-3}
Cu $K\alpha$ radiation, λ = 1.54184 Å
Cell parameters from 5749 reflections
θ = 3.2–67.7°
μ = 1.13 mm^{-1}
T = 100 K
Needle, clear colourless
0.53 × 0.05 × 0.05 mm

Data collection

XtaLAB Synergy, Dualflex, HyPix3000 diffractometer
Radiation source: micro-focus sealed X-ray tube, PhotonJet (Cu) X-ray Source
Mirror monochromator
Detector resolution: 10.0000 pixels mm^{-1}
ω scans
Absorption correction: gaussian *CrysAlis PRO* 1.171.43.90 (Rigaku Oxford Diffraction, 2023) Numerical absorption correction based on gaussian integration over a multifaceted crystal model Empirical absorption correction using spherical harmonics, implemented in SCALE3 ABSPACK scaling algorithm.
T_{min} = 0.320, T_{max} = 1.000
28359 measured reflections
5213 independent reflections
3299 reflections with $I > 2\sigma(I)$
R_{int} = 0.057
θ_{max} = 68.8°, θ_{min} = 3.2°
$h = -47 \rightarrow 44$
$k = -22 \rightarrow 47$
$l = -8 \rightarrow 8$

Refinement

Refinement on F^2
Least-squares matrix: full
$R[F^2 > 2\sigma(F^2)]$ = 0.050
$wR(F^2)$ = 0.147
S = 1.03
5213 reflections
430 parameters
7 restraints
Primary atom site location: dual
Hydrogen site location: mixed
H atoms treated by a mixture of independent and constrained refinement
$w = 1/[\sigma^2(F_o^2) + (0.0709P)^2 + 1.9674P]$ where $P = (F_o^2 + 2F_c^2)/3$
$(\Delta/\sigma)_{max}$ = 0.001
$\Delta\rho_{max}$ = 0.18 e Å^{-3}
$\Delta\rho_{min}$ = −0.23 e Å^{-3}

Special details

Geometry. All esds (except the esd in the dihedral angle between two l.s. planes) are estimated using the full covariance matrix. The cell esds are taken into account individually in the estimation of esds in distances, angles and torsion angles; correlations between esds in cell parameters are only used when they are defined by crystal symmetry. An approximate (isotropic) treatment of cell esds is used for estimating esds involving l.s. planes.
Refinement. SQUEEZE

Fractional atomic coordinates and isotropic or equivalent isotropic displacement parameters (Å²) for **9**

	x	y	z	U_{iso}*/U_{eq}
F1	0.24396 (4)	0.68423 (4)	0.59263 (18)	0.0477 (4)
O1	0.27661 (4)	0.65444 (4)	0.8928 (2)	0.0375 (4)
N1	0.21037 (5)	0.73597 (5)	0.9467 (3)	0.0461 (6)
C1	0.38419 (6)	0.65492 (6)	0.3723 (3)	0.0332 (5)
F2	0.20091 (4)	0.73648 (4)	0.6477 (2)	0.0621 (5)
O2	0.38252 (4)	0.54621 (4)	−0.1425 (2)	0.0404 (4)
C2	0.41649 (6)	0.65523 (6)	0.4934 (3)	0.0332 (6)
F3	0.21876 (4)	0.73511 (4)	1.2462 (2)	0.0573 (4)
C3	0.42077 (6)	0.62868 (6)	0.6176 (3)	0.0383 (6)
H3	0.404460	0.610774	0.622671	0.046*
F4	0.26414 (4)	0.68313 (4)	1.21789 (19)	0.0510 (4)
C4	0.44857 (6)	0.62825 (7)	0.7333 (3)	0.0421 (6)
H4	0.451517	0.609752	0.814361	0.051*
F5	0.35469 (4)	0.51146 (4)	0.1529 (2)	0.0640 (5)
C5	0.47201 (6)	0.65470 (7)	0.7310 (4)	0.0422 (6)
H5	0.490965	0.654506	0.810587	0.051*
F6	0.30410 (5)	0.46608 (5)	0.0945 (3)	0.0888 (7)
C6	0.46761 (6)	0.68113 (6)	0.6129 (4)	0.0421 (6)
H6	0.483404	0.699517	0.611946	0.051*
F7	0.30207 (4)	0.48910 (5)	−0.4996 (3)	0.0759 (6)
C7	0.44002 (6)	0.68124 (6)	0.4938 (3)	0.0367 (6)
H7	0.437488	0.699618	0.411663	0.044*
F8	0.35297 (4)	0.53562 (4)	−0.4677 (2)	0.0589 (4)
C8	0.38282 (6)	0.68649 (6)	0.2462 (3)	0.0335 (5)
C9	0.40701 (7)	0.68866 (6)	0.1090 (3)	0.0431 (6)
H9	0.423918	0.671410	0.098491	0.052*
C10	0.40683 (7)	0.71551 (7)	−0.0119 (4)	0.0487 (7)
H10	0.423665	0.716581	−0.103558	0.058*
C11	0.38241 (6)	0.74065 (6)	−0.0002 (3)	0.0402 (6)
H11	0.382439	0.759264	−0.081895	0.048*
C12	0.35795 (6)	0.73836 (6)	0.1320 (3)	0.0351 (6)
H12	0.340785	0.755398	0.140183	0.042*
C13	0.35804 (6)	0.71134 (6)	0.2540 (3)	0.0327 (5)
H13	0.340815	0.710083	0.343538	0.039*
C14	0.35394 (6)	0.65410 (6)	0.5055 (3)	0.0324 (5)
C15	0.35190 (6)	0.68029 (6)	0.6332 (3)	0.0330 (5)
H15	0.368449	0.697997	0.632565	0.040*
C16	0.32615 (6)	0.68075 (6)	0.7600 (3)	0.0328 (5)
H16	0.325000	0.698503	0.846709	0.039*
C17	0.30238 (6)	0.65525 (6)	0.7589 (3)	0.0321 (5)
C18	0.30368 (6)	0.62889 (6)	0.6389 (3)	0.0352 (6)

H18	0.2872 (6)	0.6102 (6)	0.637 (3)	0.042*
C19	0.32996 (6)	0.62835 (6)	0.5121 (3)	0.0353 (6)
H19	0.331344	0.609973	0.429194	0.042*
C20	0.38386 (6)	0.62376 (6)	0.2447 (3)	0.0329 (5)
C21	0.35649 (6)	0.62057 (6)	0.1244 (3)	0.0353 (6)
H21	0.338415	0.636681	0.129098	0.042*
C22	0.35518 (6)	0.59457 (6)	−0.0007 (3)	0.0366 (6)
H22	0.336361	0.592584	−0.080493	0.044*
C23	0.38174 (6)	0.57157 (6)	−0.0074 (3)	0.0350 (6)
C24	0.40911 (6)	0.57387 (6)	0.1067 (3)	0.0363 (6)
H24	0.4279 (6)	0.5576 (6)	0.098 (3)	0.044*
C25	0.41003 (6)	0.60007 (6)	0.2323 (3)	0.0354 (6)
H25	0.428975	0.601791	0.311442	0.043*
C26	0.25593 (6)	0.68220 (6)	0.9043 (3)	0.0327 (5)
C27	0.23929 (6)	0.69656 (6)	0.7590 (3)	0.0363 (6)
C28	0.21723 (6)	0.72293 (7)	0.7888 (4)	0.0438 (6)
C29	0.22598 (6)	0.72242 (7)	1.0827 (4)	0.0421 (7)
C30	0.24913 (6)	0.69601 (6)	1.0714 (3)	0.0371 (6)
C31	0.35491 (6)	0.52526 (6)	−0.1562 (4)	0.0390 (6)
C35	0.34084 (7)	0.51931 (7)	−0.3235 (4)	0.0466 (6)
C34	0.31504 (7)	0.49557 (8)	−0.3372 (5)	0.0557 (8)
N2	0.30303 (6)	0.47809 (6)	−0.2016 (5)	0.0629 (7)
C33	0.31629 (8)	0.48430 (7)	−0.0438 (5)	0.0596 (8)
C32	0.34171 (7)	0.50712 (7)	−0.0132 (4)	0.0482 (7)

*Atomic displacement parameters (Å^2) for **9***

	U^{11}	U^{22}	U^{33}	U^{12}	U^{13}	U^{23}
F1	0.0527 (9)	0.0591 (9)	0.0312 (8)	0.0058 (8)	−0.0061 (7)	−0.0037 (7)
O1	0.0398 (9)	0.0397 (9)	0.0329 (9)	0.0037 (8)	0.0073 (7)	0.0053 (7)
N1	0.0346 (12)	0.0443 (13)	0.0594 (16)	−0.0023 (10)	0.0096 (11)	−0.0042 (11)
C1	0.0338 (13)	0.0353 (13)	0.0307 (13)	0.0055 (11)	0.0011 (10)	0.0008 (10)
F2	0.0555 (10)	0.0645 (10)	0.0663 (11)	0.0156 (8)	−0.0116 (8)	0.0097 (9)
O2	0.0435 (10)	0.0415 (10)	0.0361 (10)	0.0036 (8)	0.0063 (8)	−0.0078 (7)
C2	0.0349 (13)	0.0367 (13)	0.0281 (13)	0.0091 (11)	0.0027 (10)	−0.0022 (10)
F3	0.0563 (9)	0.0607 (10)	0.0549 (10)	−0.0090 (8)	0.0250 (8)	−0.0191 (8)
C3	0.0385 (14)	0.0419 (14)	0.0345 (14)	0.0019 (12)	0.0025 (11)	0.0019 (11)
F4	0.0589 (9)	0.0662 (10)	0.0279 (8)	−0.0019 (8)	0.0046 (7)	0.0014 (7)
C4	0.0440 (15)	0.0479 (15)	0.0344 (14)	0.0092 (13)	−0.0017 (11)	0.0033 (12)
F5	0.0873 (12)	0.0607 (10)	0.0441 (10)	−0.0040 (9)	0.0070 (9)	0.0089 (8)
C5	0.0377 (14)	0.0505 (16)	0.0384 (15)	0.0067 (13)	−0.0024 (11)	−0.0070 (12)
F6	0.0988 (15)	0.0656 (12)	0.1019 (17)	−0.0228 (11)	0.0303 (12)	0.0144 (11)
C6	0.0369 (14)	0.0410 (15)	0.0485 (16)	0.0022 (12)	−0.0010 (12)	−0.0079 (13)
F7	0.0675 (11)	0.0711 (12)	0.0891 (15)	−0.0005 (9)	−0.0232 (10)	−0.0263 (10)
C7	0.0369 (13)	0.0336 (13)	0.0395 (14)	0.0058 (11)	0.0030 (11)	−0.0020 (11)
F8	0.0728 (11)	0.0642 (10)	0.0397 (9)	−0.0022 (9)	−0.0043 (8)	−0.0032 (7)
C8	0.0356 (13)	0.0341 (13)	0.0308 (13)	0.0041 (11)	−0.0006 (10)	0.0000 (10)
C9	0.0456 (15)	0.0437 (15)	0.0401 (15)	0.0130 (13)	0.0091 (12)	0.0062 (12)
C10	0.0510 (16)	0.0532 (17)	0.0418 (16)	0.0127 (14)	0.0145 (13)	0.0117 (13)
C11	0.0435 (15)	0.0389 (14)	0.0383 (15)	0.0037 (12)	0.0005 (12)	0.0078 (11)
C12	0.0367 (13)	0.0333 (13)	0.0353 (13)	0.0057 (11)	−0.0058 (11)	0.0000 (10)

C13	0.0338 (13)	0.0356 (13)	0.0288 (13)	0.0032 (11)	0.0001 (10)	−0.0023 (10)
C14	0.0351 (13)	0.0343 (13)	0.0277 (13)	0.0075 (11)	−0.0006 (10)	0.0018 (10)
C15	0.0349 (13)	0.0333 (13)	0.0308 (13)	0.0010 (11)	0.0011 (10)	−0.0001 (10)
C16	0.0390 (14)	0.0326 (13)	0.0269 (13)	0.0043 (11)	−0.0014 (10)	−0.0019 (10)
C17	0.0334 (13)	0.0372 (13)	0.0256 (12)	0.0027 (11)	0.0006 (10)	0.0035 (10)
C18	0.0389 (14)	0.0334 (13)	0.0332 (13)	−0.0019 (11)	0.0002 (11)	0.0014 (11)
C19	0.0400 (14)	0.0337 (13)	0.0321 (13)	0.0047 (12)	−0.0009 (11)	0.0006 (10)
C20	0.0378 (13)	0.0332 (13)	0.0278 (13)	0.0054 (11)	0.0032 (10)	0.0028 (10)
C21	0.0382 (13)	0.0377 (13)	0.0301 (13)	0.0081 (11)	0.0025 (10)	0.0004 (11)
C22	0.0396 (14)	0.0406 (14)	0.0298 (13)	0.0053 (12)	−0.0010 (11)	−0.0008 (11)
C23	0.0402 (14)	0.0345 (13)	0.0303 (13)	0.0026 (11)	0.0060 (11)	−0.0020 (10)
C24	0.0353 (13)	0.0371 (14)	0.0365 (14)	0.0061 (12)	0.0069 (11)	0.0007 (11)
C25	0.0321 (13)	0.0394 (14)	0.0348 (13)	0.0041 (11)	0.0008 (10)	0.0004 (11)
C26	0.0284 (12)	0.0331 (13)	0.0365 (14)	−0.0043 (11)	0.0050 (10)	−0.0010 (10)
C27	0.0361 (14)	0.0395 (14)	0.0333 (14)	−0.0035 (12)	0.0006 (11)	−0.0012 (11)
C28	0.0361 (14)	0.0444 (15)	0.0507 (17)	−0.0019 (13)	−0.0039 (13)	0.0042 (13)
C29	0.0365 (14)	0.0443 (15)	0.0455 (17)	−0.0087 (13)	0.0164 (12)	−0.0109 (13)
C30	0.0377 (14)	0.0450 (15)	0.0286 (13)	−0.0080 (12)	0.0044 (11)	0.0002 (11)
C31	0.0386 (14)	0.0342 (13)	0.0444 (16)	0.0055 (12)	0.0065 (12)	−0.0061 (11)
C35	0.0501 (16)	0.0425 (15)	0.0472 (15)	0.0037 (13)	0.0006 (12)	−0.0056 (11)
C34	0.0484 (17)	0.0502 (18)	0.068 (2)	0.0071 (15)	−0.0100 (16)	−0.0230 (16)
N2	0.0515 (16)	0.0417 (14)	0.096 (2)	−0.0033 (12)	0.0068 (15)	−0.0085 (15)
C33	0.062 (2)	0.0412 (17)	0.076 (2)	−0.0032 (16)	0.0179 (18)	−0.0003 (16)
C32	0.0543 (17)	0.0382 (15)	0.0520 (18)	0.0019 (14)	0.0074 (14)	−0.0013 (13)

Geometric parameters (Å, º) for ***9***

F1—C27	1.342 (3)	C11—H11	0.9500
O1—C17	1.419 (3)	C11—C12	1.376 (3)
O1—C26	1.360 (3)	C12—H12	0.9500
N1—C28	1.309 (3)	C12—C13	1.395 (3)
N1—C29	1.297 (3)	C13—H13	0.9500
C1—C2	1.554 (3)	C14—C15	1.402 (3)
C1—C8	1.554 (3)	C14—C19	1.380 (3)
C1—C14	1.546 (3)	C15—H15	0.9500
C1—C20	1.547 (3)	C15—C16	1.382 (3)
F2—C28	1.339 (3)	C16—H16	0.9500
O2—C23	1.414 (3)	C16—C17	1.367 (3)
O2—C31	1.362 (3)	C17—C18	1.366 (3)
C2—C3	1.402 (3)	C18—H18	0.98 (2)
C2—C7	1.375 (3)	C18—C19	1.397 (3)
F3—C29	1.345 (3)	C19—H19	0.9500
C3—H3	0.9500	C20—C21	1.403 (3)
C3—C4	1.389 (3)	C20—C25	1.387 (3)
F4—C30	1.337 (3)	C21—H21	0.9500
C4—H4	0.9500	C21—C22	1.381 (3)
C4—C5	1.386 (3)	C22—H22	0.9500
F5—C32	1.348 (3)	C22—C23	1.378 (3)
C5—H5	0.9500	C23—C24	1.371 (3)
C5—C6	1.369 (4)	C24—H24	0.98 (2)
F6—C33	1.341 (4)	C24—C25	1.390 (3)

C6—H6	0.9500	C25—H25	0.9500
C6—C7	1.399 (3)	C26—C27	1.382 (3)
F7—C34	1.336 (3)	C26—C30	1.382 (3)
C7—H7	0.9500	C27—C28	1.366 (3)
F8—C35	1.337 (3)	C29—C30	1.379 (3)
C8—C9	1.396 (3)	C31—C35	1.381 (4)
C8—C13	1.377 (3)	C31—C32	1.380 (4)
C9—H9	0.9500	C35—C34	1.378 (4)
C9—C10	1.385 (3)	C34—N2	1.308 (4)
C10—H10	0.9500	N2—C33	1.307 (4)
C10—C11	1.377 (3)	C33—C32	1.358 (4)

C26—O1—C17	116.77 (17)	C18—C17—C16	122.1 (2)
C29—N1—C28	116.4 (2)	C17—C18—H18	123.6 (15)
C8—C1—C2	111.87 (19)	C17—C18—C19	118.7 (2)
C14—C1—C2	104.68 (18)	C19—C18—H18	117.7 (14)
C14—C1—C8	112.17 (18)	C14—C19—C18	121.0 (2)
C14—C1—C20	111.75 (18)	C14—C19—H19	119.5
C20—C1—C2	111.65 (17)	C18—C19—H19	119.5
C20—C1—C8	104.92 (18)	C21—C20—C1	117.95 (19)
C31—O2—C23	117.38 (17)	C25—C20—C1	124.3 (2)
C3—C2—C1	118.3 (2)	C25—C20—C21	117.6 (2)
C7—C2—C1	123.6 (2)	C20—C21—H21	119.2
C7—C2—C3	118.0 (2)	C22—C21—C20	121.6 (2)
C2—C3—H3	119.6	C22—C21—H21	119.2
C4—C3—C2	120.8 (2)	C21—C22—H22	120.7
C4—C3—H3	119.6	C23—C22—C21	118.6 (2)
C3—C4—H4	119.9	C23—C22—H22	120.7
C5—C4—C3	120.2 (2)	C22—C23—O2	120.1 (2)
C5—C4—H4	119.9	C24—C23—O2	118.0 (2)
C4—C5—H5	120.3	C24—C23—C22	121.7 (2)
C6—C5—C4	119.4 (2)	C23—C24—H24	120.5 (14)
C6—C5—H5	120.3	C23—C24—C25	119.0 (2)
C5—C6—H6	119.8	C25—C24—H24	120.4 (14)
C5—C6—C7	120.4 (2)	C20—C25—C24	121.3 (2)
C7—C6—H6	119.8	C20—C25—H25	119.3
C2—C7—C6	121.2 (2)	C24—C25—H25	119.3
C2—C7—H7	119.4	O1—C26—C27	123.9 (2)
C6—C7—H7	119.4	O1—C26—C30	119.0 (2)
C9—C8—C1	117.8 (2)	C30—C26—C27	117.0 (2)
C13—C8—C1	124.2 (2)	F1—C27—C26	120.7 (2)
C13—C8—C9	117.8 (2)	F1—C27—C28	120.6 (2)
C8—C9—H9	119.4	C28—C27—C26	118.7 (2)
C10—C9—C8	121.2 (2)	N1—C28—F2	116.8 (2)
C10—C9—H9	119.4	N1—C28—C27	124.8 (2)
C9—C10—H10	119.8	F2—C28—C27	118.4 (2)
C11—C10—C9	120.4 (2)	N1—C29—F3	117.1 (2)
C11—C10—H10	119.8	N1—C29—C30	124.8 (2)
C10—C11—H11	120.6	F3—C29—C30	118.1 (3)
C12—C11—C10	118.9 (2)	F4—C30—C26	120.1 (2)
C12—C11—H11	120.6	F4—C30—C29	121.5 (2)

C11—C12—H12	119.6	C29—C30—C26	118.4 (2)
C11—C12—C13	120.9 (2)	O2—C31—C35	119.1 (2)
C13—C12—H12	119.6	O2—C31—C32	123.4 (2)
C8—C13—C12	120.8 (2)	C32—C31—C35	117.3 (3)
C8—C13—H13	119.6	F8—C35—C31	120.1 (2)
C12—C13—H13	119.6	F8—C35—C34	121.7 (3)
C15—C14—C1	117.6 (2)	C34—C35—C31	118.3 (3)
C19—C14—C1	124.1 (2)	F7—C34—C35	118.3 (3)
C19—C14—C15	118.2 (2)	N2—C34—F7	117.5 (3)
C14—C15—H15	119.5	N2—C34—C35	124.1 (3)
C16—C15—C14	121.0 (2)	C33—N2—C34	116.9 (3)
C16—C15—H15	119.5	F6—C33—C32	118.9 (3)
C15—C16—H16	120.5	N2—C33—F6	116.7 (3)
C17—C16—C15	119.0 (2)	N2—C33—C32	124.4 (3)
C17—C16—H16	120.5	F5—C32—C31	120.1 (2)
C16—C17—O1	119.8 (2)	F5—C32—C33	120.9 (3)
C18—C17—O1	117.9 (2)	C33—C32—C31	119.0 (3)

F1—C27—C28—N1	178.1 (2)	C14—C1—C2—C7	−118.8 (2)
F1—C27—C28—F2	−1.1 (3)	C14—C1—C8—C9	−174.4 (2)
O1—C17—C18—C19	−176.7 (2)	C14—C1—C8—C13	1.4 (3)
O1—C26—C27—F1	−2.8 (3)	C14—C1—C20—C21	64.5 (3)
O1—C26—C27—C28	175.3 (2)	C14—C1—C20—C25	−120.3 (2)
O1—C26—C30—F4	3.3 (3)	C14—C15—C16—C17	−0.4 (3)
O1—C26—C30—C29	−174.9 (2)	C15—C14—C19—C18	2.0 (3)
N1—C29—C30—F4	−179.6 (2)	C15—C16—C17—O1	177.3 (2)
N1—C29—C30—C26	−1.4 (4)	C15—C16—C17—C18	1.5 (3)
C1—C2—C3—C4	−177.9 (2)	C16—C17—C18—C19	−0.9 (3)
C1—C2—C7—C6	176.3 (2)	C17—O1—C26—C27	50.2 (3)
C1—C8—C9—C10	178.1 (2)	C17—O1—C26—C30	−134.0 (2)
C1—C8—C13—C12	−177.9 (2)	C17—C18—C19—C14	−1.0 (3)
C1—C14—C15—C16	−178.1 (2)	C19—C14—C15—C16	−1.4 (3)
C1—C14—C19—C18	178.5 (2)	C20—C1—C2—C3	−64.3 (3)
C1—C20—C21—C22	176.4 (2)	C20—C1—C2—C7	120.1 (2)
C1—C20—C25—C24	−175.8 (2)	C20—C1—C8—C9	−52.9 (3)
O2—C23—C24—C25	175.5 (2)	C20—C1—C8—C13	122.9 (2)
O2—C31—C35—F8	−4.1 (4)	C20—C1—C14—C15	177.8 (2)
O2—C31—C35—C34	173.9 (2)	C20—C1—C14—C19	1.2 (3)
O2—C31—C32—F5	4.7 (4)	C20—C21—C22—C23	−0.7 (4)
O2—C31—C32—C33	−173.5 (2)	C21—C20—C25—C24	−0.6 (3)
C2—C1—C8—C9	68.3 (3)	C21—C22—C23—O2	−175.2 (2)
C2—C1—C8—C13	−115.9 (2)	C21—C22—C23—C24	0.2 (4)
C2—C1—C14—C15	56.8 (2)	C22—C23—C24—C25	0.1 (4)
C2—C1—C14—C19	−119.8 (2)	C23—O2—C31—C35	132.8 (2)
C2—C1—C20—C21	−178.6 (2)	C23—O2—C31—C32	−52.9 (3)
C2—C1—C20—C25	−3.4 (3)	C23—C24—C25—C20	0.2 (4)
C2—C3—C4—C5	1.9 (4)	C25—C20—C21—C22	0.9 (3)
F3—C29—C30—F4	0.0 (3)	C26—O1—C17—C16	58.6 (3)
F3—C29—C30—C26	178.2 (2)	C26—O1—C17—C18	−125.5 (2)
C3—C2—C7—C6	0.7 (3)	C26—C27—C28—N1	−0.1 (4)
C3—C4—C5—C6	−0.4 (4)	C26—C27—C28—F2	−179.3 (2)

C4—C5—C6—C7	−1.0 (4)	C27—C26—C30—F4	179.4 (2)
C5—C6—C7—C2	0.8 (4)	C27—C26—C30—C29	1.2 (3)
F6—C33—C32—F5	0.2 (4)	C28—N1—C29—F3	−178.8 (2)
F6—C33—C32—C31	178.3 (2)	C28—N1—C29—C30	0.8 (4)
F7—C34—N2—C33	179.0 (2)	C29—N1—C28—F2	179.2 (2)
C7—C2—C3—C4	−2.0 (3)	C29—N1—C28—C27	0.0 (4)
F8—C35—C34—F7	−0.2 (4)	C30—C26—C27—F1	−178.7 (2)
F8—C35—C34—N2	177.6 (3)	C30—C26—C27—C28	−0.6 (3)
C8—C1—C2—C3	178.5 (2)	C31—O2—C23—C22	−55.6 (3)
C8—C1—C2—C7	2.9 (3)	C31—O2—C23—C24	128.9 (2)
C8—C1—C14—C15	−64.7 (3)	C31—C35—C34—F7	−178.2 (2)
C8—C1—C14—C19	118.7 (2)	C31—C35—C34—N2	−0.3 (4)
C8—C1—C20—C21	−57.3 (3)	C35—C31—C32—F5	179.1 (2)
C8—C1—C20—C25	117.9 (2)	C35—C31—C32—C33	1.0 (4)
C8—C9—C10—C11	−0.6 (4)	C35—C34—N2—C33	1.1 (4)
C9—C8—C13—C12	−2.1 (3)	C34—N2—C33—F6	−179.4 (3)
C9—C10—C11—C12	−0.9 (4)	C34—N2—C33—C32	−0.9 (4)
C10—C11—C12—C13	0.9 (4)	N2—C33—C32—F5	−178.2 (3)
C11—C12—C13—C8	0.6 (4)	N2—C33—C32—C31	−0.1 (4)
C13—C8—C9—C10	2.0 (4)	C32—C31—C35—F8	−178.7 (2)
C14—C1—C2—C3	56.8 (3)	C32—C31—C35—C34	−0.8 (4)

Hydrogen-bond geometry (Å, º) for ***9***

D—H···*A*	*D*—H	H···*A*	*D*···*A*	*D*—H···*A*
C18—H18···F5^{i}	0.98 (2)	2.59 (2)	3.241 (3)	124.2 (18)
C24—H24···F3^{ii}	0.98 (2)	2.58 (2)	3.513 (3)	159.2 (19)

Symmetry codes: (i) −*y*+3/4, *x*+1/4, *z*+1/4; (ii) *y*−1/4, −*x*+3/4, *z*−5/4.
